# Electrostatic-induced ion-confined partitioning in graphene nanolaminate membrane for breaking anion–cation co-transport to enhance desalination

**DOI:** 10.1038/s41467-024-48681-8

**Published:** 2024-05-21

**Authors:** Haiguang Zhang, Jiajian Xing, Gaoliang Wei, Xu Wang, Shuo Chen, Xie Quan

**Affiliations:** Key Laboratory of Industrial Ecology and Environmental Engineering (Ministry of Education, China), School of Environmental Science and Technology, Dalian University of Technology, Dalian, 116024 China

**Keywords:** Pollution remediation, Mechanical and structural properties and devices, Environmental, health and safety issues

## Abstract

Constructing nanolaminate membranes made of two-dimensional graphene oxide nanosheets has gained enormous interest in recent decades. However, a key challenge facing current graphene-based membranes is their poor rejection for monovalent salts due to the swelling-induced weak nanoconfinement and the transmembrane co-transport of anions and cations. Herein, we propose a strategy of electrostatic-induced ion-confined partitioning in a reduced graphene oxide membrane for breaking the correlation of anions and cations to suppress anion-cation co-transport, substantially improving the desalination performance. The membrane demonstrates a rejection of 95.5% for NaCl with a water permeance of 48.6 L m^−2^ h^−1^ bar^−1^ in pressure-driven process, and it also exhibits a salt rejection of 99.7% and a water flux of 47.0 L m^−2^ h^−1^ under osmosis-driven condition, outperforming the performance of reported graphene-based membranes. The simulation and calculation results unveil that the strong electrostatic attraction of membrane forces the hydrated Na^+^ to undergo dehydration and be exclusively confined in the nanochannels, strengthening the intra-nanochannel anion/cation partitioning, which refrains from the dynamical anion-cation correlations and thereby prevents anions and cations from co-transporting through the membrane. This study provides guidance for designing advanced desalination membranes and inspires the future development of membrane-based separation technologies.

## Abstract (web summary)

Two-dimensional graphene-based membranes have gained much interest, but they suffer from poor rejection for monovalent salts. Here, the authors develop an electrostatic-induced ion partitioning strategy to suppress anion-cation transmembrane co-transport, improving the desalination performance.

## Introduction

Membrane technologies offer great promise for applications in water purification and various chemical separations. With the discoveries of nanomaterials, a great breakthrough has been made towards developing high-performance membranes. Graphene, as a two-dimensional (2D) atom-thick layered nanomaterial, has been considered as an exciting candidate for the construction of new-generation membranes^1–3^. Typically, graphene-based nanolaminate membranes such as graphene oxide (GO) membranes possess 2D nanochannels with both pristine graphitic regions and oxidized regions, featuring ultrafast water transport and concurrently ultra-selective sieving for molecules and ions^4,5^. Their sub-nanometer interlayer spaces with tailorable fine architecture and surface chemistry are propitious for water desalination, ion separation, and valuable element recovery^2,6,7^. Benefiting from the nanoconfined interlaminar channels and the smooth *sp*^2^ carbon surface, GO membranes can exhibit superior water permeability over conventional commercial membranes^8,9^. However, a serious challenge facing GO-based membranes is their poor rejection for small salt ions (e.g., typically 10–60% for monovalent NaCl)^10–13^, which imposes an impediment to their potential application in the field of desalination. The main reasons is the undesirable swelling of GO membranes in aqueous solution due to the water-affinity nature of GO^14^, weakening the interlayer space nanoconfinement. Consequently, the weak ion barrier ability of the membrane pore allows ions to penetrate through the membrane, resulting in low ion rejection^15,16^. In recent years, great efforts have been taken to restrain the membrane swelling^17–19^, but these manipulations generally narrow the interlayer spacing, causing a significant decrease of water permeation. Thus, constructing advanced high-permeable graphene-based membranes with high monovalent ion rejection ability still remains a major bottleneck.

In-depth understanding of the intrinsic mechanism underlying the membrane-based ion separation process is essential and instructive for designing membranes and improving ion rejection performance, which necessitates exploration of the ion behavior and ion transport in nanoconfined membrane pore channels^20–22^. Recent studies have revealed that strong ion–ion dynamical correlations are existed during ion transport in nanoconfined pore channels^23,24^, strongly affecting the intra-nanochannel ion diffusion. In addition, we have also noted in our recent studies that the free mobile anions and cations could be correlated in the transmembrane transport process^25,26^, and they can co-transport through the membrane nanochannels due to the electroneutrality condition^24,27–30^. This means that the anion–cation correlated co-transport phenomenon leads to low ion rejection. Thus, seeking effective strategy to overcome this ion–ion correlation and suppress the co-transport of anion–cation pairs could be expected to effectively improve the ion rejection performance of the membrane.

The ion transport through nano- or subnano-meter membrane pore has been demonstrated to be governed by the intrapore diffusion of ions^27,31^, which are affected by the ion-pore wall interactions^16,32^, generally electrostatic attractive and repulsive interactions of fixed charges on the membrane pores^33,34^. This suggests the importance of ion-pore interactions and intrapore anion/cation regulations. In nano- or subnano-confined charged membrane channels, the cations and anions would be rearranged by the channel walls, creating overlapped electric double layer (EDL)^35^ and forming anion/cation partitioning^26,36,37^. The unbalanced ion distribution can render the co-ion (ions with the same charges to the charged channels) depletion and the counter-ion (ions with the opposite charge to the charged channels) accumulation^38^, forming a counter-ion-enriched nanochannel^2,17,39,40^. Such a counter-ion channel could exclude co-ions and salt from the confined nanofluid^24,41–43^. Inspired by this, constructing a counter-ion-confined nanochannel (like an exclusive cationic or anionic nanochannel) could be able to dissociate the free mobile anion–cation pairs and thereby abolish their co-transport behavior, which requires strong intra-nanochannel ion partitioning for confining counter-ions in the nanochannels. Therefore, the regulation of anion/cation partitioning in nanoconfined membrane channels could open up a possibility to break the ion–ion dynamical correlations, suppressing the anion–cation co-transport so as to improve salt rejection.

Here, we assemble sodium polystyrenesulfonate (PSSNa) into amine-crosslinked reduced GO (rGO) nanolaminates to construct a cation-confined ArGO-PSSNa membrane and propose an electrostatic-induced ion-confined partitioning strategy to enhance monovalent ion rejection while maintaining high water permeation. By synchronous rGO-interlayer confinement and PSSNa-embedded cation-affinity interaction, the counter-ions (cations) can be confined into the nanolaminates to enhance the intra-nanochannel anion/cation partitioning, leading to the significant improvement of monovalent salt rejection. As a result, the membrane exhibits a water permeance of 48.6 L m^−2^ h^−1^ bar^−1^ and a rejection rate of 95.5% for 5 mM NaCl in pressure-driven process, which is superior to other reported graphene-based nanolaminate membranes. Moreover, the membrane shows 99.7% rejection for 0.5 M NaCl with a water flux of 47.0 L m^−2^ h^−1^ in osmosis-driven condition, outperforming the membranes reported in recent researches. This work demonstrates that the intramembrane ion partitioning regulation is an effective strategy for suppressing anion–cation correlated co-transport to improve salt rejection performance.

## Results

### Membrane fabrication and characterization

**Fig. 1:**
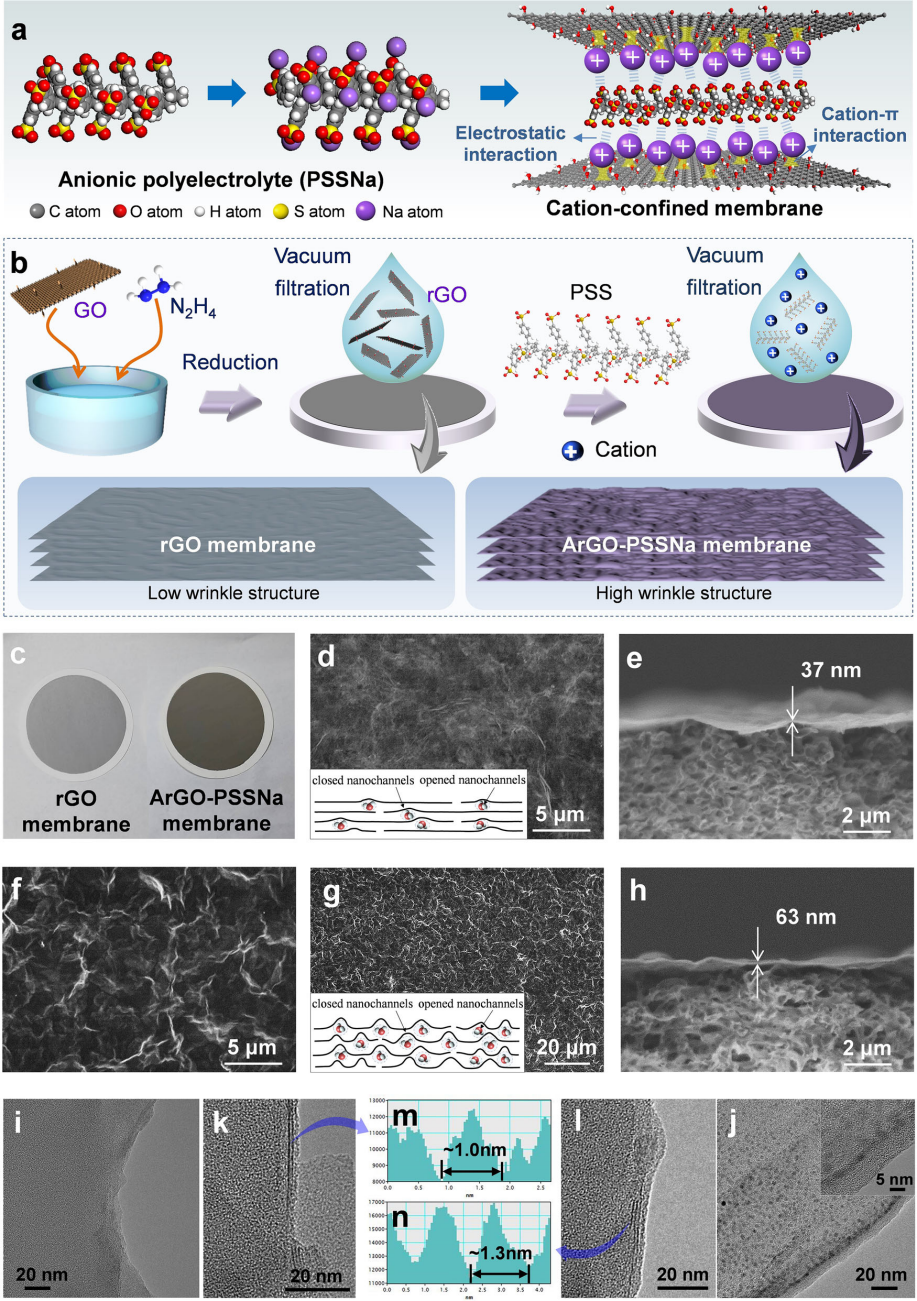
Preparation and structure of ArGO-PSSNa membrane. **a** Structural illustration of ArGO-PSSNa membrane. **b** Schematic diagrams of the preparation procedure and the wrinkle structures of rGO and ArGO-PSSNa membranes. **c** Digital photographs of rGO and ArGO-PSSNa membranes. **d**, **e** Top and cross-section SEM images of rGO membrane. The inset of **d** shows the schematic diagram of the wrinkle structures of the rGO membrane. **f**, **g** Top SEM images of ArGO-PSSNa membrane. The inset of **g** shows the schematic diagram of the wrinkle structures of the ArGO-PSSNa membrane. **h** Cross-section view of ArGO-PSSNa membrane. **i**–**l**, TEM images of rGO membrane (**i**, **k**) and ArGO-PSSNa membrane (**j**, **l**). The inset of **j** shows the high-resolution TEM image of the ArGO-PSSNa membrane. **m**, **n** Interlayer spacing measurements of rGO membrane (**m**) and ArGO-PSSNa membrane (**n**) using the DigitalMicrograph software.

The ArGO-PSSNa membrane was constructed by embedding anionic polyelectrolyte of PSSNa into amine-crosslinked rGO (ArGO) interlayers. Among them, PSSNa with abundant negatively charged sulfonic groups can electrostatically attract cations in water into the membrane nanochannels, and there are also cation−oxygen-group and cation−π-electron interactions between the rGO nanosheets and cations, allowing the membrane to enrich cations and concurrently confine them in the nanochannels (Fig. 1a). The ArGO-PSSNa membrane was fabricated by a vacuum filtration method followed by in-situ polymerization crosslinking (Fig. 1b). The rGO nanosheets obtained by hydrazine reduction of GO were vacuum-filtered on a polyvinylidene difluoride (PVDF) substrate to yield a rGO membrane, and a solution containing PSSNa and m-phenylenediamine is then filtered. The membrane was subsequently soaked in a trimesoyl chloride solution for polymerization crosslinking to form the ArGO-PSSNa membrane. The digital photos depict that the rGO membrane appears light gray color, while the ArGO-PSSNa membrane is darker, meaning a thicker membrane functional layer (Fig. 1c). The scanning electron microscope (SEM) images show that the surface of rGO membrane is flat and has a slightly wrinkle structure (Fig. 1d). The membrane thickness is approximately 37 nm (Fig. 1e). By contrast, the ArGO-PSSNa membrane possesses a prominent and undulating wrinkle surface (Fig. 1f). From the low-magnification SEM image, it can be seen that a large number of distinguishable wrinkles are uniformly distributed on the membrane surface (Fig. 1g and Supplementary Fig. 1). This wrinkle structure may be caused by the rapid polymerization reaction and spontaneous diffusion of PSSNa within rGO nanolaminates during the polymerization and heat treatment processes. For rGO membrane, the flat nanosheet stacking (dense stacking) can lead to narrow rGO-interlayer spacing, making most interlayer free spacings less than the size of water molecules (called water-impermeable closed nanochannels) (inset of Fig. 1d)^44^. Whereas, the PSSNa embeddedness in the ArGO-PSSNa membrane makes lots of wrinkles, allowing more interlayer free spacings to be larger than the size of water molecules (called water-permeable opened nanochannels) (inset of Fig. 1g), which may be conducive to facilitating water permeation. The thickness of ArGO-PSSNa membrane becomes ~63 nm (Fig. 1h), which is consistent with the observed thickness by atomic force microscopy (AFM) (Supplementary Fig. 2). The transmission electron microscope (TEM) images display that the rGO membrane is lamellar structure, and smooth, homogeneous, no impurities (Fig. 1i); whereas the ArGO-PSSNa membrane shows many speckles with the size of ~1 nm (Fig. 1j). Moreover, the membrane interlayer spacing increases from 1 nm for rGO to 1.3 nm for ArGO-PSSNa (Fig. 1k–n). The presence of speckles and the increase of interlayer spacing suggest the embeddedness of PSSNa into rGO nanolaminates.

**Fig. 2:**
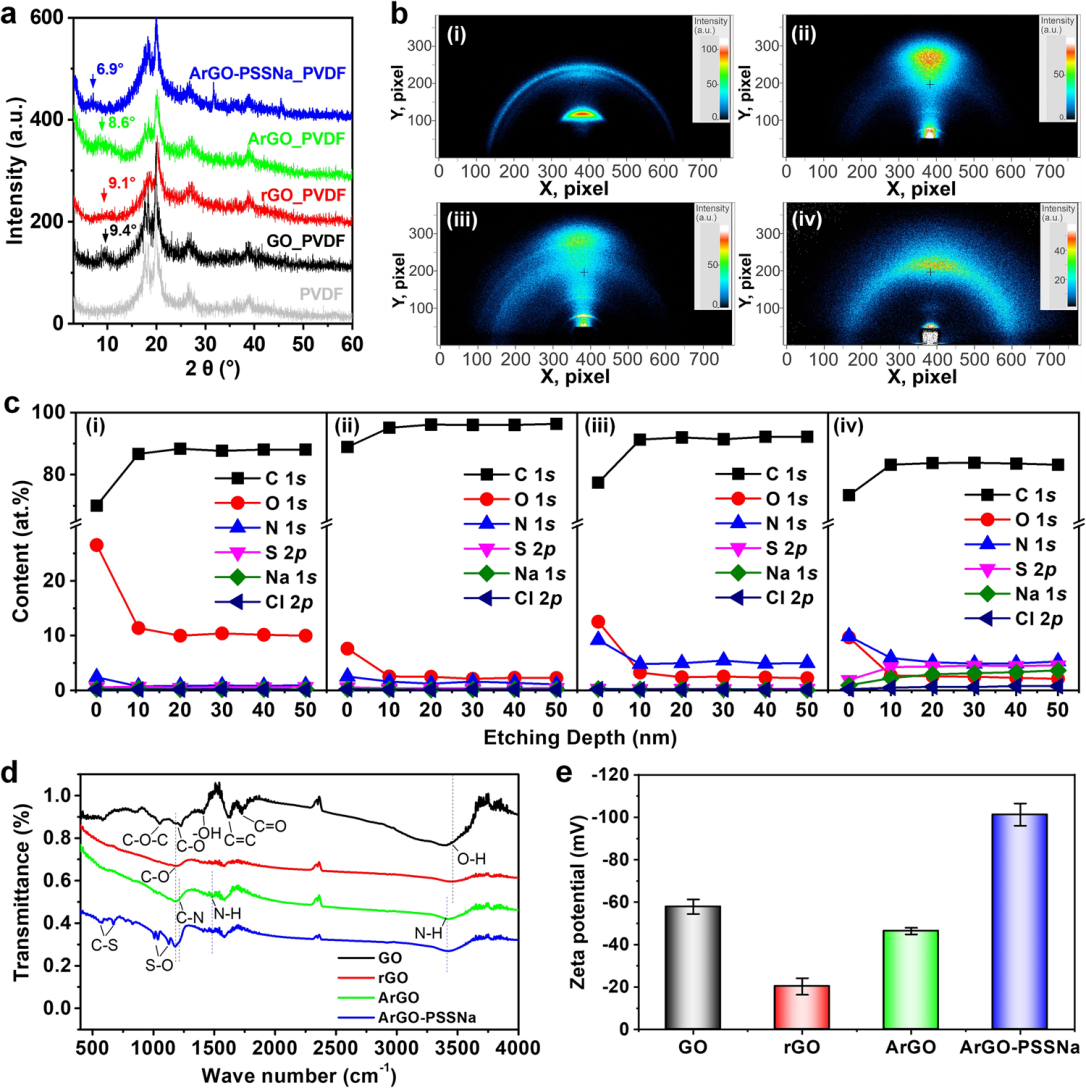
Characterization of ArGO-PSSNa membrane structures and properties. **a** XRD diffraction patterns of PVDF substrate, GO, rGO, ArGO, and ArGO-PSSNa membranes. **b** 2D-WAXD patterns of GO (i), rGO (ii), ArGO (iii), and ArGO-PSSNa (iv) membranes. **c** Variation of element content over XPS etching depth in GO (i), rGO (ii), ArGO (iii), and ArGO-PSSNa (iv) membranes. The lines between symbols are guides to the eye. **d** FTIR spectra of GO, rGO, ArGO, and ArGO-PSSNa membranes. The dashed lines are guide to the eye. **e** Zeta potentials of GO, rGO, ArGO, and ArGO-PSSNa membranes. Error bars represent the standard deviation of three replicate measurements. Source data are provided as a Source Data file.

Four membranes of GO, rGO, ArGO, and ArGO-PSSNa were prepared to contrastively investigate the evolutions of membrane microstructure and chemical properties. These membranes show progressively darker color changing from GO to ArGO-PSSNa, and the membrane thickness first decreases from 86 nm for GO to 37 nm for rGO and then increases to 63 nm for ArGO-PSSNa (Supplementary Fig. 3). The variation in membrane thickness implies the change of membrane interlayer spacing. Further, the interlayer spacings were analyzed by X-ray diffraction (XRD) characterization. Compared with the XRD pattern of PVDF substrate, a peak located at 9.4° appears on the pattern of GO membrane, while the characteristic peak of rGO membrane becomes weak and moves to 9.1° (corresponding to an interlayer spacing of ~9.7 Å) (Fig. 2a). After amine-crosslinking, the characteristic peak assigned to ArGO membrane is shifted left to 8.6°, and the peak further shifts left to 6.9° for ArGO-PSSNa membrane, corresponding to an interlayer spacing of 12.8 Å, which is consistent with the observed interlayer spacing from the TEM images. The 2D wide-angle XRD (2D-WAXD) patterns show that the chemical reduction of GO to rGO renders the diffraction arc for GO to change as a diffraction spot for rGO (Fig. 2b), suggesting that the rGO nanolaminates are stacked more regularly and orderly. On the contrary, the amine-crosslinking and PSSNa embeddedness cause the 2D-WAXD pattern to gradually change as a diffraction arc, demonstrating that the disordered degree of the nanolaminate membrane is increased. In other words, the amine-crosslinking and PSSNa embeddedness lead to more wrinkle structure. This result is consistent with the wrinkle-enriched morphology shown in the SEM images of ArGO-PSSNa membrane.

The chemical compositions of the ArGO-PSSNa membrane were further characterized. By distinguishing the energy-dispersive X-ray spectroscopy mapping images, it is found that the ArGO-PSSNa membrane contains C, O, N, S, and Na elements (Supplementary Fig. 4), agreeing with the results of X-ray photoelectron spectra (XPS) (Supplementary Fig. 5). The XPS depth-profiling spectra further shows that the chemical reduction of GO leads to a significantly lower O content of ~2.3 at.% in rGO membrane compared with that in GO membrane (~10.0 at.%) (Fig. 2c), and the C content of rGO membrane is as high as 96.3 at.%, in which the proportion of *sp*^2^ carbon reaches 80.0% (Supplementary Fig. 6 and Supplementary Table 1). By comparison, a lower C content of 83.2 at.% is exhibited for the ArGO-PSSNa membrane, while the N, S, and Na contents reach 5.3, 4.6, and 3.7 at.%, respectively. Besides, the Fourier transform infrared spectroscopy (FTIR) shows that the C–N, N–H, S–O, and C–S characteristic peaks appear in the spectrum of the ArGO-PSSNa membrane (Fig. 2d), further confirming the amine crosslinking and PSSNa embeddedness in the composite membrane. Benefiting from the embeddedness of PSSNa, the Zeta potential of ArGO-PSSNa membrane reaches −102.4 mV (Fig. 2e and Supplementary Figs. 7−8), which is significantly higher than that of rGO and ArGO membranes (−20.3 and −46.4 mV, respectively), implying that the ArGO-PSSNa membrane possesses excellent negative charge property.

### Membrane filtration performance

**Fig. 3:**
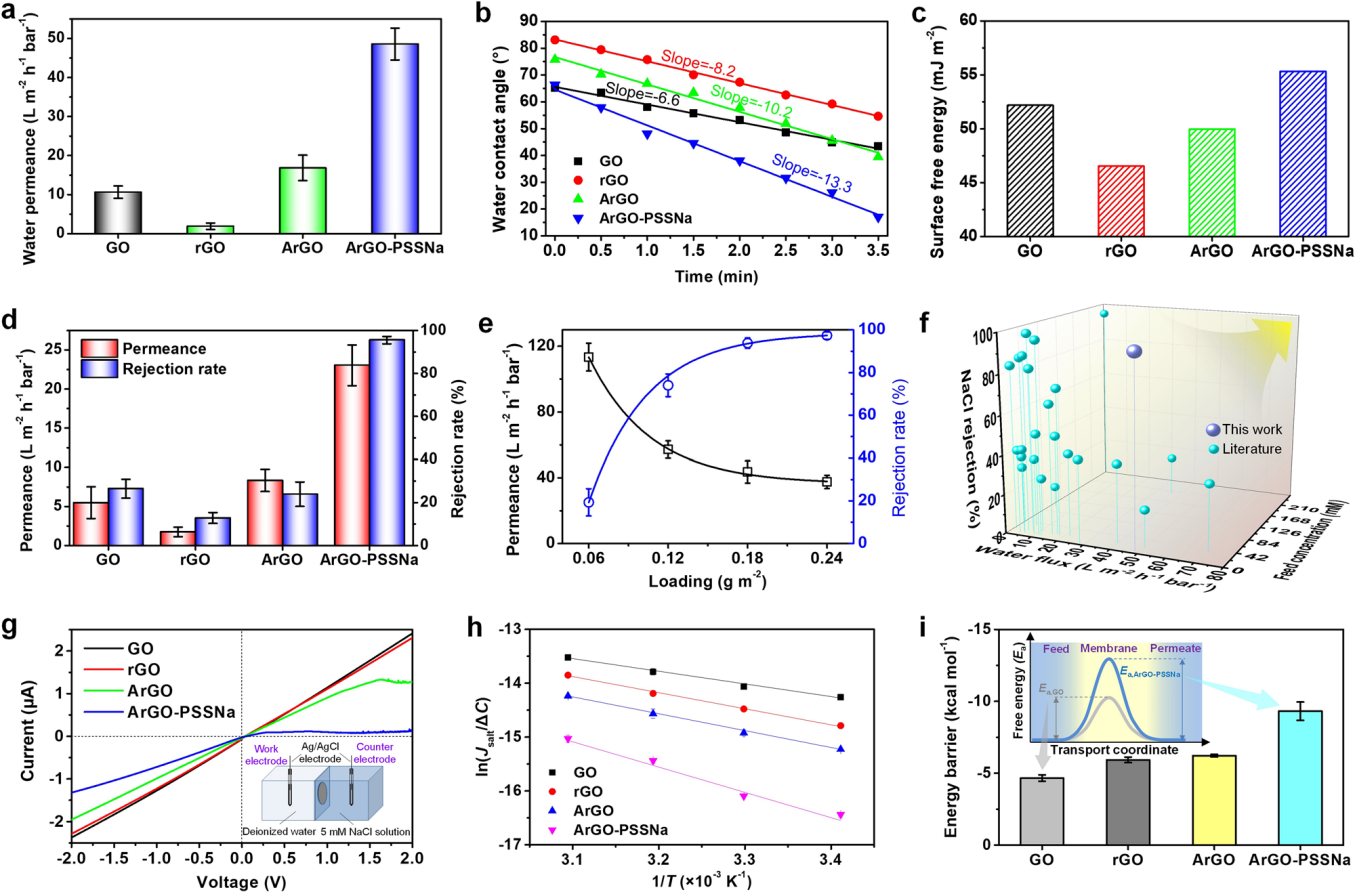
Membrane performance evaluation. **a** Pure water permeances of GO, rGO, ArGO, and ArGO-PSSNa membranes. **b** Time variations of water contact angles for GO, rGO, ArGO, and ArGO-PSSNa membranes. Ultrapure water serves as the test solution. The lines denote the linear fits made with the numerical model. **c** Surface free energies of GO, rGO, ArGO, and ArGO-PSSNa membranes calculated according to the contact angle measurements. **d** Water permeances and salt rejection rates of GO, rGO, ArGO, and ArGO-PSSNa membranes for filtering a 5 mM NaCl solution under a transmembrane pressure of 5 bar. **e** Water permeances and NaCl rejection rates of ArGO-PSSNa membranes with different ArGO-PSSNa loadings. The solid lines are drawn as guides to the eye. **f** Performance comparison of ArGO-PSSNa membrane with the graphene-based membranes reported in previous literatures. The lines are drawn as guides to the eye. The detailed performance parameters of membranes and the references are shown in Supplementary Table 3. **g** *I*−*V* curves of GO, rGO, ArGO, and ArGO-PSSNa membranes between a deionized water and a 5 mM NaCl solution. The inset of **g** shows the diagram of electrochemical two-compartment cell. Two Ag/AgCl electrodes were used for testing the *I*–*V* curves. **h** Arrhenius-type plots for NaCl salt transport through GO, rGO, ArGO, and ArGO-PSSNa membranes from 5 mM NaCl solution to DI water. Natural logarithm of the salt flux divided by salt concentration difference of two cells (*J*_salt_/ΔC) is plotted as a function of 1/*T*. The lines denote the linear fits made with the numerical model. **i** Energy barriers for NaCl salt during the transmembrane process through GO, rGO, ArGO, and ArGO-PSSNa membranes from 5 mM NaCl solution to DI water. The inset of **i** shows the diagram of transmembrane energy barrier profiles for salt transported through GO and ArGO-PSSNa membranes. Error bars represent the standard deviation of three replicate measurements. Source data are provided as a Source Data file.

The ArGO-PSSNa membrane with the embeddedness of PSSNa has wrinkle-enriched structure and increased membrane interlayer spacing, giving high pure water permeance of 48.6 L m^−2^ h^−1^ bar^−1^ (Fig. 3a and Supplementary Figs. 9−10), which is 4.5 times higher than that of the GO membrane (10.7 L m^−2^ h^−1^ bar^−1^) and 25.6 times higher than that of the rGO membrane (1.9 L m^−2^ h^−1^ bar^−1^). One of the reasons for the high water permeance of the ArGO-PSSNa membrane is its larger interlayer spacing (1.28 nm) than that of GO and rGO membranes (0.94–0.97 nm), which can reduce the energy barrier of water transport through the membrane. According to the Hagen-Poiseuille equation (showing that the water mass flow per unit area is proportional to the third power of the interlayer spacing, see Supplementary Equation 1)^1^, the water permeance theoretically increases by only 2.3–2.5 times when the membrane interlayer spacing enlarges from 0.94–0.97 nm to 1.28 nm. Such an increase is lower than the experimental 4.5 and 25.6 times (higher than the GO and rGO membranes respectively), suggesting that the enlargement of the interlayer spacing is not the sole reason for improved water permeance.

Due to the excellent water affinity of PSSNa, it could be inferred that the increase in water permeance may be related to the hydrophilicity of the membrane. Figure 3b and Supplementary Fig. 11 show the dynamic water contact angles of GO, rGO, ArGO, and ArGO-PSSNa membranes. Obviously, the water contact angle of the rGO membrane is larger than that of the GO membrane. Despite worse hydrophilicity, a faster decline rate of 8.16°/min in dynamic water contact angle is presented for the rGO membrane compared with that for the GO membrane (6.57°/min). After amine polymerization and PSSNa embeddedness, the hydrophilicity is improved gradually. Especially, for the ArGO-PSSNa membrane, the water contact angle drastically reduces from 66.3° to 17.0° with a high decline rate of 13.3°/min in dynamic contact angle, suggesting that the embeddedness of PSSNa enhances the water wettability of the membrane, which could reduce water transport resistance in membrane nanochannels. According to the Owens–Wendt–Rabel–Kaelble (OWRK) formula (Supplementary Fig. 12 and Supplementary Table 2)^45^, the surface free energies were calculated based on the water and diiodomethane contact angles of the membranes. The rGO membrane shows a surface free energy of 46.5 mJ m^−2^ (Fig. 3c), lower than that of the GO membrane (52.2 mJ m^−2^). Particularly, the amine crosslinking and PSSNa embeddedness can increase the surface free energy until it reaches 55.3 mJ m^−2^ for the ArGO-PSSNa membrane. It is worth noting that the surface free energies show a similar variation trend to the pure water permeances for four membranes, indicating that high surface free energy is conducive to water permeation through the membrane. The improvement of surface free energy can lead to enhanced dipole moment interactions between water molecules and the membrane, which makes it easier for water molecules to be adsorbed to enter and penetrate through the membrane (Supplementary Fig. 13). Thus, the high water permeability of ArGO-PSSNa membrane is ascribed to the synergistic effects of enlarging interlayer spacing and improving surface free energy for decreasing water transport resistance. Benefitting from the superior hydrophilic nature and negatively charged property, the ArGO-PSSNa membrane possesses good fouling-resistant ability, showing only 15% decrease in flux when filtering 0.2 g L^−1^ bovine serum albumin (BSA) solution (Supplementary Fig. 14), which is significantly lower than that of GO and rGO membranes (flux decline ratio: 55%).

The salt rejection performances of the membranes were further evaluated, as shown in Fig. 3d. After the chemical reduction, the permeation flux and NaCl rejection rate are respectively decreased from 5.5 L m^−2^ h^−1^ bar^−1^ and 26.5% for GO membrane to 1.7 L m^−2^ h^−1^ bar^−1^ and 12.8% for rGO membrane when filtering 5 mM NaCl solution. In contrast, the amine crosslinking and PSSNa embeddedness can substantially improve the permeation flux and NaCl rejection rate. Especially, the ArGO-PSSNa membrane shows a permeation flux of 23.0 L m^−2^ h^−1^ bar^−1^, which is 13.5 times higher than that of the rGO membrane. Meanwhile, the NaCl rejection rate reaches 95.5%, which is 7.5 times as high as that of the rGO membrane. During a 40-h filtration, the membrane can maintain the water permeance of 19–23 L m^−2^ h^−1^ bar^−1^ and the NaCl rejection rate of 90–95% (Supplementary Fig. 15). With the ArGO-PSSNa loading increasing from 0.06 to 0.24 g m^−2^, a water permeation−NaCl rejection trade-off is displayed (Fig. 3e). The ArGO-PSSNa membrane can exhibit the NaCl rejection rates of 80–97% for salt concentrations of 2.5~100 mM (Supplementary Fig. 16). The salt concentration up to 100 mM is usually in the typical concentration range of most brackish waters, meaning that the ArGO-PSSNa membrane is promising for brackish water desalination. When both permeation and rejection are taken into account, the monovalent salt rejection performance of the ArGO-PSSNa membrane is significantly superior to that of reported GO-based membranes (typically 10–60% for NaCl) (Fig. 3f and Supplementary Table 3)^46^. Besides, the ArGO-PSSNa membrane also shows >96% rejection for Na_2_SO_4_ and MgSO_4_, and the rejection rates of KCl and MgCl_2_ are maintained above 90% (Supplementary Fig. 17), demonstrating that the ArGO-PSSNa membrane can efficiently reject other salts and the salt with higher ion valence ratio of anion to cation leads to higher rejection rate.

In order to investigate the ion transmembrane behavior for understanding the ion rejection mechanism, the ion transports through the GO, rGO, ArGO, and ArGO-PSSNa membranes were evaluated using a two-compartment cell filled with a 5 mM NaCl solution and deionized water (the inset in Fig. 3g and Supplementary Fig. 18). The electrochemical current-voltage (*I*–*V*) curves show that, when negative voltages from 0 to −2.0 V are applied (driving Na^+^ transport), the measured electric currents are linearly increased from 0 to −2.4 μA for GO membrane and −1.3 μA for ArGO-PSSNa membrane, indicating that Na^+^ can pass through the membranes and the ArGO-PSSNa membrane shows the largest Na^+^ transport resistance. On the contrary, when positive voltages from 0 to 2.0 V are applied (driving Cl^−^ transport), the current linearly increases from 0 to ~2.4 μA for both GO and rGO membranes, whereas the current exhibits a nonlinearly increase at a slower rate for ArGO membrane, suggesting that the transport of Cl^−^ is hampered. More dramatically, for the ArGO-PSSNa membrane, there is no significant increase in current as the voltage increases, which remains at a low value of 0.1 μA (Supplementary Fig. 19), implying that Cl^−^ ions are difficult to traverse through the ArGO-PSSNa membrane. This result demonstrates the ionic selectivity of the ArGO-PSSNa membrane and suggests that this membrane is a cation-confined membrane, which makes it difficult for anions to permeate through the membrane. Besides, the different transport behavior of Na^+^ and Cl^−^ driven by the electric field also means that the ArGO-PSSNa membrane could be able to suppress the anion–cation co-transport phenomenon so as to increase the transmembrane resistance of NaCl salt. Essentially, the transmembrane transport of salt needs to overcome all the resistances caused by membrane nanochannels such as steric, electrostatic, and dehydration effects, which can be explained by analyzing the transmembrane energy barrier of salt transport. We performed temperature-dependent NaCl salt flux measurements to experimentally determine the energy barriers of GO, rGO, ArGO, and ArGO-PSSNa membranes using the Arrhenius-type equation (Supplementary Equation 6 and Supplementary Figs. 20, 21). All of four Arrhenius-type plots show good linear fitting (Fig. 3h). The calculated energy barrier of ArGO-PSSNa membrane reaches −9.3 kcal mol^−1^ (Fig. 3i), markedly higher than that of GO, rGO, and ArGO membranes (−4.7, −5.9 and −6.2 kcal mol^−1^, respectively), indicating that the NaCl salt is more difficult to traverse through ArGO-PSSNa membrane. The high energy barrier of the ArGO-PSSNa membrane could be related to the cation-confined nanochannel structure of the membrane.

### Investigation of salt rejection mechanism

**Fig. 4:**
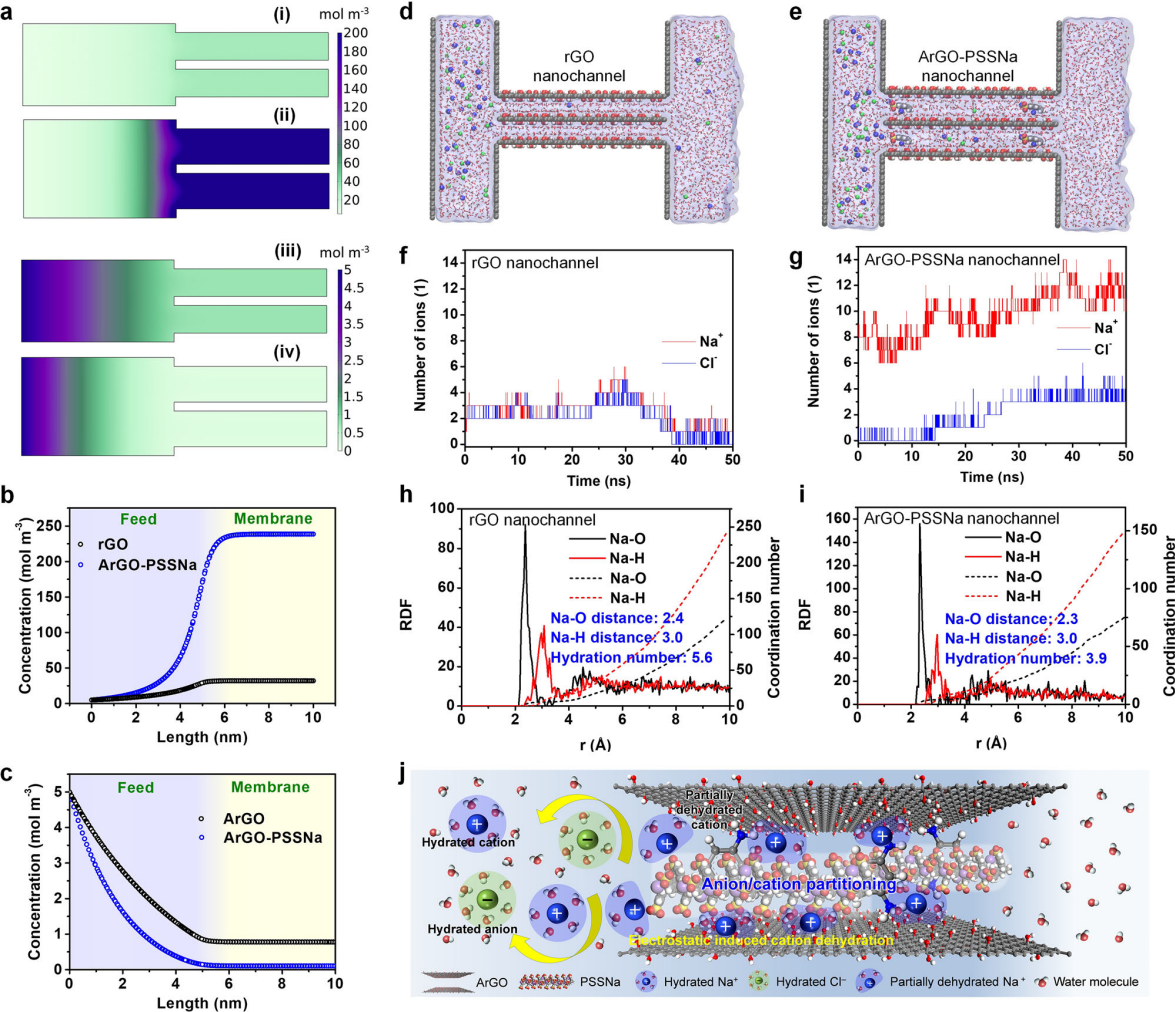
Simulations of ion distribution and transport behavior in rGO and ArGO-PSSNa nanochannels. **a** Simulated ion concentration profiles in the feed and membrane nanochannels. (i) Cation (Na^+^) concentration in rGO membrane nanochannels; (ii) Cation (Na^+^) concentration in ArGO-PSSNa membrane nanochannels; (iii) Anion (Cl^–^) concentration in rGO membrane nanochannels; (iv) Anion (Cl^–^) concentration in ArGO-PSSNa membrane nanochannels. **b** Cation (Na^+^) concentration variations from the feed side to the rGO and ArGO-PSSNa membrane nanochannels. **c** Anion (Cl^–^) concentration variations from the feed side to the rGO and ArGO-PSSNa membrane nanochannels. **d** Snapshots of the MD simulations for NaCl solution transporting through the rGO nanochannels. **e** Snapshots of the MD simulations for NaCl solution transporting through the ArGO-PSSNa nanochannels. The C, O, H, S, Na, and Cl atoms are shown in gray, red, white, yellow, blue, and green spheres, respectively. **f** Time evolution of the number of Na^+^ and Cl^–^ in the rGO nanochannels. **g** Time evolution of the number of Na^+^ and Cl^–^ in the ArGO-PSSNa nanochannels. **h**, **i** MD-calculated RDF and coordination numbers for oxygen and hydrogen of water molecules around the Na^+^ ions in the feed side (**h**) and ArGO-PSSNa nanochannels (**i**). The solid lines represent the RDF, and the dashed lines denote the coordination numbers. **j** Schematic diagram of ion distribution behavior in the feed side and ArGO-PSSNa nanochannels. Source data are provided as a Source Data file.

The spatial distributions of cations and anions in the nanochannels of rGO and ArGO-PSSNa membranes were further comparatively investigated by COMSOL Multiphysics (Supplementary Figs. 22–25). The simulated cation (Na^+^) concentration in the rGO nanochannel is 32 mol m^–3^ (Fig. 4a(i), b), and the anion (Cl^–^) concentration is 0.78 mol m^–3^ (Fig. 4a(iii), c), indicating that the negatively charged surface induces ion rearrangement, causing unbalanced distributions of cations and anions in the nanochannels. By comparison, the ArGO-PSSNa nanochannel possesses a much higher Na^+^ concentration of 240 mol m^–3^ and a lower Cl^–^ concentration of only 0.09 mol m^–3^ (Fig. 4a(ii, iv)–c), corresponding to a cation/anion ratio of 2667, which is approximately 65 times higher than that of the rGO nanochannel (the cation/anion ratio is 41). This result suggests that the ArGO-PSSNa nanochannels can induce stronger partitioning of cations and anions, demonstrating the intra-nanochannel cation-confinement architecture of the ArGO-PSSNa membrane. The ion rearrangement and partitioning were further investigated by in-situ monitoring of electrochemical current change. When the NaCl solution comes into contact with the membrane, the current change for the ArGO-PSSNa membrane reaches 0.82 μA (Supplementary Fig. 26), significantly higher than that of the rGO membrane (approximately 0.16 μA). Such a larger current change for the ArGO-PSSNa membrane indicates that more ions undergo rearrangement in the membrane, leading to stronger ion partitioning.

In order to explore the intrinsic mechanism of the ion partitioning effect to enhance ion rejection, molecular dynamics (MD) simulations^9,47–50^ were conducted based on the nanochannel structures of rGO and ArGO-PSSNa membranes to investigate the desalination process and ion transport behavior (Supplementary Figs. 27–31 and Supplementary Tables 4, 5). After the simulations, we found that almost the same number of Na^+^ and Cl^–^ transporting through the rGO nanochannels, seeming to be co-transported in the anion–cation-pair-like formation. As shown in Fig. 4d, the MD simulation snapshot shows that 4 Na^+^ and 4 Cl^–^ traverse through the rGO nanochannels when filtering 1380 of water molecules, corresponding to a salt rejection of 68.1%; while no ions transport through the ArGO-PSSNa nanochannels, corresponding to a 100% salt rejection (Fig. 4e and Supplementary Fig. 28). The mean square displacements (MSD) indicate that the transport rate of Na^+^ through ArGO-PSSNa nanochannels is significantly suppressed due to the existence of PSSNa in the nanochannels (Supplementary Fig. 29). Notably, the number of Na^+^ and Cl^–^ in the ArGO nanochannels is equivalent, averaging approximately 2 Na^+^ and 2 Cl^–^ in the entire simulation period (Fig. 4f), which demonstrates no ion partitioning. As for the ArGO-PSSNa nanochannels, 10 Na^+^ are existed, much higher than the number of Cl^–^ (approximately 2 Cl^–^) (Fig. 4g). Correspondingly, the ArGO-PSSNa nanochannels exhibit higher number density of Na^+^ (0.0015 atoms nm^−3^) and lower number density of Cl^−^ (0.0002 atoms nm^−3^) compared with the rGO nanochannels (0.0003 atoms nm^−3^ for both Na^+^ and Cl^−^) (Supplementary Fig. 30), demonstrating that the ArGO-PSSNa nanochannels can strengthen intra-nanochannel ion partitioning. The above results suggest that the high salt rejection of the ArGO-PSSNa membrane is caused by the strong ion partitioning effect that can effectively inhibit the transport of salt through the membrane.

The partitioning effect of ions from the feed side into the narrow membrane nanochannels could be related to the dehydration of ions, because of the strong nanoconfinement and electrostatic attraction interactions between the nanochannel walls and ions. In order to further investigate the ion dehydration, the radial distribution functions (RDF) of Na^+^ ions were calculated from the MD simulation for analyzing the coordination (hydration) of Na^+^ ions. In the first hydrated shell of the hydrated Na^+^, the distance between the Na^+^ and the oxygen of the water molecule is approximately 2.4 Å, and the distance for the hydrogen of the water molecule is 3.0 Å (Fig. 4h, i). By comparing the RDF of hydrated Na^+^ in the feed and ArGO-PSSNa nanochannels, it can be found that there is no change in the distance between the Na^+^ and corresponding coordinated water molecules after the hydrated Na^+^ entering the nanochannels. However, the average coordination number (hydration number) of hydrated Na^+^ ions in the nanochannels is 3.9, which is lower than that in the feed (average coordination number: 5.6), indicating the partial dehydration of hydrated Na^+^ ions in the nanochannels. The ArGO-PSSNa nanochannels can electrostatically attracts Na^+^ ions due to the negatively charged rGO surface and sulfonic acid groups of PSS. These electrostatic attraction interactions can overcome the hydration shell (hydration energy) of hydrated Na^+^, allowing partially dehydrated Na^+^ to be electrostatically confined in the nanochannels, which strengthens the ion partitioning effect (Fig. 4j). This breaks the dynamical correlations between the free mobile anion–cation pairs, preventing anions and cations from co-transporting through the membrane so as to improve the salt rejection performance.

### Osmosis-driven membrane performance and mechanism analysis

**Fig. 5:**
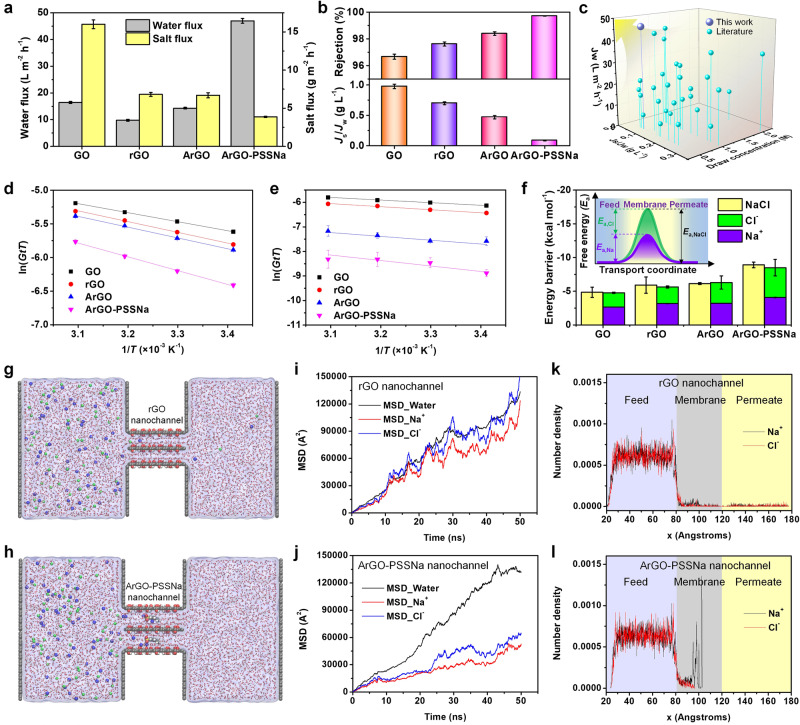
Performance and mechanism of osmosis-driven membrane processes. **a** Water fluxes and NaCl permeation fluxes of GO, rGO, ArGO, and ArGO-PSSNa membranes. The tests were conducted at room temperature under FO (the active layer facing the feed solution) mode using 0.5 M NaCl and DI water as the draw and feed solutions, respectively. **b** Salt rejection rates and *J*_s_/*J*_w_ of GO, rGO, ArGO, and ArGO-PSSNa membranes. **c** Performances of ArGO-PSSNa membrane and the membranes in literatures. The lines are drawn as guides to the eye. The detailed membrane performance parameters and the references are shown in Supplementary Table 6. **d**, **e** Arrhenius-type plots for individual cation (Na^+^) and anion (Cl^–^) transporting through GO, rGO, ArGO, and ArGO-PSSNa membranes from 0.5 M NaCl solution to DI water. The natural logarithm of the lumped parameter (*GtT*) was plotted as a function of 1/*T*. The lines denote the linear fits made with the numerical model. **f** Energy barriers of salt (NaCl), cation (Na^+^), and anion (Cl^–^) during the transmembrane process through GO, rGO, ArGO, and ArGO-PSSNa membranes from 0.5 M NaCl solution to DI water. The inset of **f** shows the diagram of transmembrane energy barrier profiles that the energy barrier of NaCl is equal to the sum of energy barriers of Na^+^ and Cl^–^. **g**, **h** Snapshots of MD simulations for osmosis-driven desalination processes of rGO (**g**) and ArGO-PSSNa (**h**) nanochannels. The C, O, H, S, Na, and Cl atoms are shown in gray, red, white, yellow, blue, and green spheres, respectively. **i**, **j** Time evolutions of MSD of water molecule, Na^+^, and Cl^–^ in the entire simulation period for rGO (**i**) and ArGO-PSSNa (**j**) nanochannels. **k**, **l** Average number density distributions of Na^+^ and Cl^–^ in the feed side, nanochannel, and permeate side during the entire simulation period for rGO (**k**) and ArGO-PSSNa (**l**) nanochannels. Error bars represent the standard deviation of three replicate measurements. Source data are provided as a Source Data file.

In the above exploration, it is noted that the ArGO-PSSNa membrane possesses strong ion partitioning effect and high ion transport energy barrier, suppressing the transmembrane penetration of salt, which may not only improve the pressure-driven salt rejection but also have great potential for water desalination through osmosis-driven processes. Further, the performance of GO, rGO, ArGO, and ArGO-PSSNa membranes was next evaluated through a forward osmosis (FO) mode. The result shows that, among these four membranes, the ArGO-PSSNa membrane exhibits the highest water flux of 47.0 L m^−2^ h^−1^ and the lowest NaCl permeation flux of 3.8 g m^−2^ h^−1^ (Fig. 5a and Supplementary Fig. 32). Based on the water flux and salt flux, the salt rejection rate was calculated following an order of *R*_GO_ < *R*_rGO_ < *R*_ArGO_ < *R*_ArGO-PSSNa_, with a maximum value of 99.7% (Fig. 5b). Correspondingly, the salt-water ratio (*J*_s_/*J*_w_) decreases from 0.97 g L^−1^ for GO membrane to 0.08 g L^−1^ for ArGO-PSSNa membrane, demonstrating significantly improved water-salt selectivity. During a 40-h operation, the water flux is in the range of 42~47 L m^−2^ h^−1^ and the salt rejection maintains at 99.5–99.7% (Supplementary Fig. 33). With the increase of NaCl concentration from 0.05 to 1.0 M, both water and salt fluxes of ArGO-PSSNa membrane are increased, and the rejection rate improves from 97.3% to 99.8% (Supplementary Fig. 34). Figure 5c compares the water flux and salt-water ratio of ArGO-PSSNa membrane with other membranes in recent studies. The water flux of our membrane is higher than other 2D nanolaminate membranes, and it is also approximately 5–10 times as high as the typical value of FO membranes^51–54^. Besides, the desalination performance can outperform the reported membranes in recent research works (Supplementary Table 6).

The ion transmembrane transport behavior in osmosis-driven process is further investigated by analyzing the transmembrane energy barrier using the Arrhenius equation. Good linear fitted Arrhenius-type plots are exhibited for NaCl transporting through GO, rGO, ArGO, and ArGO-PSSNa membranes (Supplementary Figs. 35–37). The calculated energy barrier increases from −4.8 kcal mol^−1^ for the GO membrane to −8.9 kcal mol^−1^ for the ArGO-PSSNa membrane. Considering the possible different transport behavior of cations and anions, we used an electric field to decouple the transmembrane transports of Na^+^ and Cl^–^ ions (Supplementary Figs. 38–41), obtaining the corresponding Arrhenius-type plots (Fig. 5d, e). It is found that the sum of energy barriers of individual Na^+^ and Cl^–^ is equal to the total energy barrier of NaCl (Fig. 5f), and the energy barrier of Na^+^ is slightly higher than that of Cl^–^, which agrees with the result in previous studies^27^. The higher energy barrier of Na^+^ could be caused by the stronger electrostatic attraction interactions between the nanochannel and Na^+^, which leads to energy-favorable binding sites.

The results of MD simulations further demonstrate that the ArGO-PSSNa membrane nanochannels possess higher ion transport energy barrier than the rGO nanochannels. The Na^+^ and Cl^–^ ions can traverse through the rGO nanochannels but cannot penetrate through the ArGO-PSSNa nanochannels (Fig. 5g, h and Supplementary Figs. 42, 43). Besides, the MSD result indicates that the diffusion rates of Na^+^ and Cl^–^ through ArGO-PSSNa nanochannels are significantly decreased, compared with the ion diffusion through rGO nanochannels (Fig. 5i, j). The analysis of ion density distribution demonstrates that Na^+^ is indeed confined in the ArGO-PSSNa nanochannels (Fig. 5k, l and Supplementary Fig. 44), causing significant ion partitioning. Notably, the Na^+^ ions are not uniformly distributed throughout the nanochannels but are located near the specific sites around the PSS in the nanochannels, suggesting strong electrostatic attraction interaction between PSS and Na^+^. More interestingly, the RDF results show that the rGO nanochannels with lower salt rejection have a stronger Na^+^ dehydration (the hydration number is 2.4) (Supplementary Fig. 45), while the ArGO-PSSNa nanochannels with higher salt rejection exhibit a weaker Na^+^ dehydration (the hydration number is 4.4). This indicates that the sole ion dehydration effect cannot lead to improved salt rejection, which further implies that the intra-nanochannel Na^+^ confinement could be critically affected by the electrostatic attraction interactions between Na^+^ and the embedded PSS as well as rGO surface (Supplementary Fig. 46).

### DFT calculation of ion-membrane interactions

**Fig. 6:**
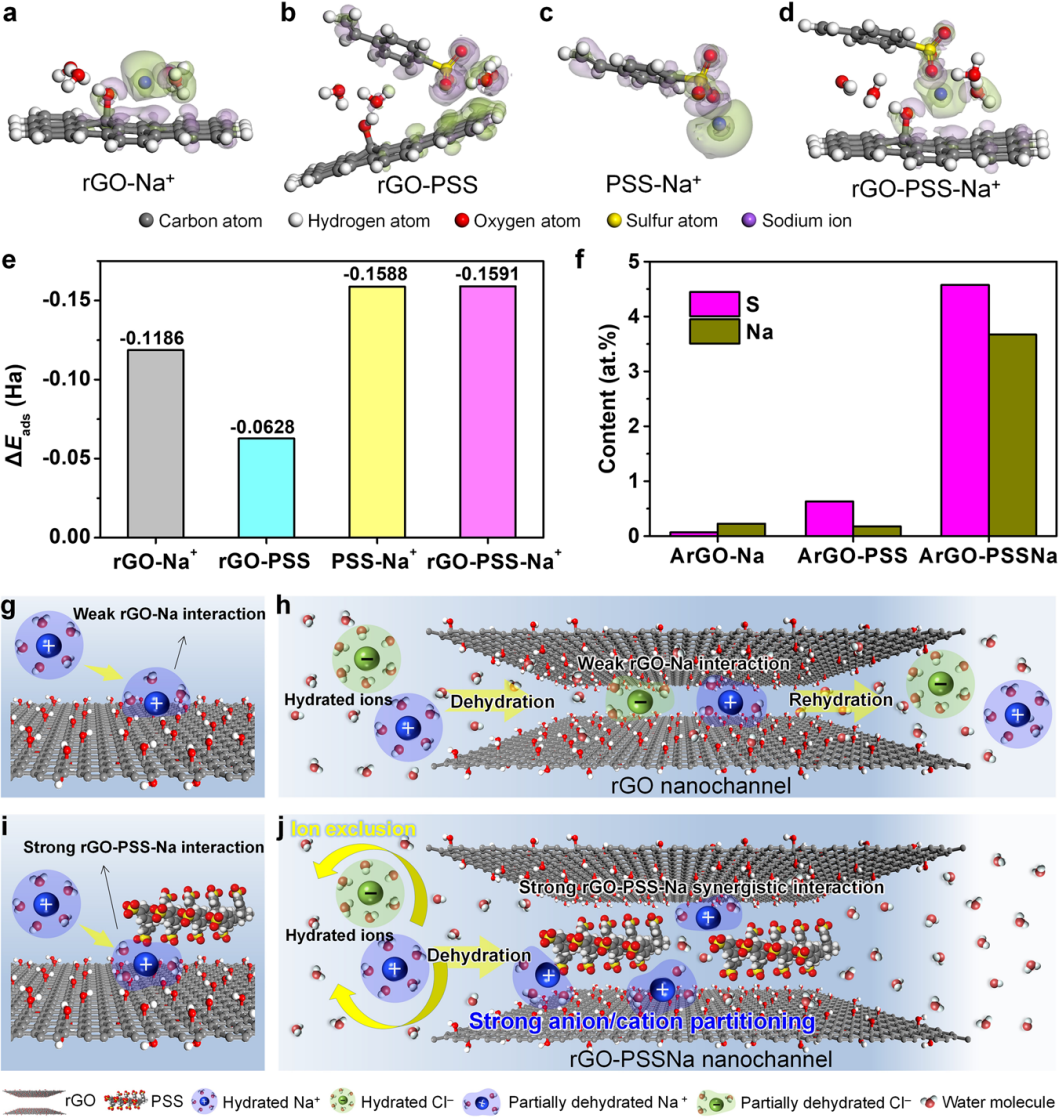
Interactions between ArGO-PSSNa membrane and ions. **a**–**d** DFT-calculated electron density difference distributions between rGO and Na^+^ (**a**), rGO and PSS (**b**), PSS and Na^+^ (**c**), and rGO, PSS and Na^+^ (**d**). The blue and green regions represent the accumulation and depletion of electron density, respectively, and the isosurface is 0.01 e A^−3^. **e** DFT-calculated adsorption energies of rGO-Na^+^, rGO-PSS, PSS-Na^+^, and rGO-PSS-Na^+^. **f** Element contents of S and Na in XPS etching spectra of ArGO-Na, ArGO-PSS, and ArGO-PSSNa membranes. **g**–**j** Schematics of the ion-membrane interactions and ion transport behavior in the ArGO (**g**, **h**) and ArGO-PSSNa (**i**, **j**) membrane nanochannels. Source data are provided as a Source Data file.

The interaction between the membrane and Na^+^ was investigated using the density functional theory (DFT) calculation to determine the intra-nanochannel ion-confined partitioning mechanism. The DFT results show that both rGO and PSS have electrostatically attractive effects on Na^+^, and the adsorption energies are −0.12 and −0.16 Ha, respectively (Fig. 6a, c, e and Supplementary Table 7). By comparison, a weaker interaction is existed between the rGO and PSS, showing an adsorption energy of −0.06 Ha (Fig. 6b, e). Besides, the adsorption energy between Na^+^ and phenylenediamine monomer is calculated as only −0.012 Ha, and the adsorption energy between Na^+^ and the crosslinked polymer with trimesoyl chloride is in a range of −0.046~−0.057 Ha (Supplementary Fig. 47 and Supplementary Table 8), which are significantly lower than the adsorption energy of PSS-Na^+^ (−0.16 Ha). Especially, the adsorption energy between rGO, PSS, and Na^+^ (rGO-PSS-Na^+^) is calculated as −0.16 Ha, which is consistent with the adsorption energy of PSS-Na^+^. Thus, it is inferred that the strong electrostatic attractive PSS-Na^+^ interaction is dominant in the intra-nanochannel ion partitioning, while the rGO-Na^+^ interaction takes second place. Moreover, we fabricated three membranes of ArGO-Na, ArGO-PSS, and ArGO-PSSNa and analyzed their XPS etching spectra to further determine the electrostatic attractive interactions between rGO, PSS, and Na^+^ (Fig. 2c and Supplementary Fig. 48). The Na content in the ArGO-Na membrane is only 0.2 at.%, indicating that it is difficult to individually confine Na^+^ in the ArGO nanolaminates (Fig. 6f). For ArGO-PSS membrane, a low S content of 0.6 at.% suggests that PSS cannot stably exist in the membrane alone because of weak adsorption energy between rGO and PSS. Noteworthily, the S and Na contents in the ArGO-PSSNa membrane are respectively 4.6 and 3.7 at.%, which are much higher than those in the ArGO-Na and ArGO-PSS membranes. This result highlights the combined effects of the electrostatic attraction interactions between rGO, PSS, and Na^+^ in the ArGO-PSSNa membrane. That is, the strong intra-nanochannel Na^+^ confinement is caused by both electrostatic attractive PSS-Na^+^ interaction and rGO-Na^+^ interaction. Thus, it could be concluded that the strong ion partitioning effect is contributed by the synergistic electrostatic attractive interactions between Na^+^ and PSS as well as rGO.

By comprehensive analysis of the electrostatic interactions, ion dehydration, ion partitioning, ion co-transport behavior, and energy barrier, we propose the intrinsic mechanism of salt rejection enhanced by intra-nanochannel ion-confined partitioning regulation. Specifically, for rGO membrane nanochannels, the electrostatic attraction interaction between Na^+^ and rGO can induce the hydrated Na^+^ to enter the GO nanochannels and undergo ionic dehydration (Fig. 6g). However, this relatively weak rGO-Na^+^ attraction interaction force has no ability to confine the dehydrated Na^+^ ions in the nanochannels (Fig. 6h and Supplementary Fig. 49). Consequently, the Na^+^ and accompanying Cl^−^ are co-transported through the nanochannels in the form of free mobile anion–cation pairs (governed by the electrical neutrality principle), achieving ionic rehydration in the permeate side and thereby resulting in low ion rejection. In contrast, for ArGO-PSSNa membrane nanochannels, the synergistic effect of electrostatic attractive PSS-Na^+^ and rGO-Na^+^ interactions can enable hydrated Na^+^ to undergo ionic dehydration after entering the nanochannels (Fig. 6i). Owing to the relatively strong synergistic PSS-Na^+^ and rGO-Na^+^ attraction forces, the dehydrated Na^+^ ions are electrostatically confined in the nanochannels, which results in the accumulation of Na^+^ and corresponding dissipation of Cl^−^ in the nanochannels, forming strong intra-nanochannel anion/cation partitioning (Fig. 6j). This strong ion partitioning can break the free mobile anion–cation dynamical correlations, making it difficult to form co-transferable anion–cation pairs, which synchronously increases the transmembrane energy barriers of anions and cations. This effectively prevents both anions and cations for co-transporting through the membrane nanochannels, resulting in high salt rejection.

## Discussion

We design and construct a cation-confined ArGO-PSSNa nanolaminate membrane that combines the functions of rGO-interlayer confinement and strong cation-affinity of sulfonic groups, by which the intra-nanochannel anion/cation partitioning is significantly reinforced to improve the salt rejection performance. Thanks to the embeddedness of PSSNa, the membrane possesses a wrinkle-enriched microstructure for well-developed pore nanochannels with high surface free energy, endowing the membrane with high electronegativity and hydrophilicity. The water permeance reaches 48.6 L m^−2^ h^−1^ bar^−1^, which is respectively 4.5 and 25.6 times higher than that of the GO and rGO membranes. Meanwhile, by the strong cation confinement of ArGO-PSSNa nanochannels, a significant anion/cation partitioning is exhibited, leading to a rejection rate of 95.5% for NaCl, significantly higher than that of GO-based membranes reported previously. Besides, we also demonstrate that the membrane has a high salt rejection rate of 99.7% with a water flux of 47.0 L m^−2^ h^−1^ when operating under osmosis-driven condition, outperforming the reported membranes in recent researches. Our work presents a synchronous regulation strategy of graphene-induced fast water transport property and electrostatic-induced ion-confined partitioning effect, giving an effective approach to concurrently achieve high water permeability and high desalination performance, which is expected to assist the predictive design of membrane-based selective water or ion nanochannels.

By theoretical MD simulations and DFT calculations, we reveal that the synergistic electrostatic attractive PSS-Na^+^ and rGO-Na^+^ interactions induce hydrated Na^+^ to undergo ionic dehydration, allowing the Na^+^ to be strongly confined in the nanochannels, strengthening the anion/cation partitioning effect. This breaks the dynamical anion–cation correlations, inhibiting the formation of free mobile anion–cation pairs, which prevents the transmembrane co-transport phenomenon of anions and cations so as to improve the salt rejection performance. Here, we comprehensively relate the ion rejection performance with the ion-membrane attraction interaction, ion dehydration, ion partitioning, ion co-transport behavior, and ion energy barrier, which presents a substantial in-depth understanding of the membrane desalination mechanism. Emphatically, it is essential to precisely regulate the membrane nanochannels and ion-membrane interactions for simultaneously satisfying fast water permeation, intra-nanochannel cation confinement, and expulsions of anion and salt. Future work may involve the design of ion-confined membranes and the exploration of inherent relations between membrane channels and various ions. Our findings could provide guidance for developing membrane-based principles and technologies for nanofluid transport and ion separation processes.

## Methods

### Preparation of ArGO-PSSNa nanolaminate membrane

The ArGO-PSSNa membrane was fabricated by a method of vacuum filtration followed by in-situ polymerization crosslinking. GO nanosheets were first synthesized using the modified Hummers’ method with natural flake graphite powder (2000 mesh), and the rGO nanosheets were prepared by the hydrazine-assisted GO reduction method^26^. Briefly, the GO nanosheets were dispersed into ultrapure water with ultrasonication to form a GO dispersion (0.05 mg mL^−1^). Afterwards, 50 mL GO dispersion was put into a 100 mL Teflon-lined stainless-steel autoclave and mixed with 100 μL ammonia solution (25% in water), and then 20 μL hydrazine hydrate solution (80 wt.%) was added. Subsequently, the autoclave was placed in an oven and maintained at 90 °C for 2 h to obtain black rGO nanosheets dispersion.

For preparing the ArGO-PSSNa nanolaminate membrane, 4 mL as-prepared rGO dispersion (0.05 mg mL^−1^) was mixed with 5 mL PSSNa (1.0 wt.% in 1 M NaCl solution) and 5 mL m-phenylenediamine (2.0 wt.%), which was then diluted to a 20 mL mixture with water. After ultrasonication for 10 min, the mixture was filtrated on a PVDF membrane (0.1 μm pore size; 47 mm diameter) substrate with vacuum assistance. After removing the excess solution, the resulting membrane was soaked in 0.1 w/v% trimesoyl chloride solution in n-hexane for 60 s. The excess solution on the membrane was drained off. Finally, the membrane was cured at 80 °C for 10 min in an oven and rinsed using deionized water to obtain the ArGO-PSSNa composite membrane.

### Characterization of membrane structures and properties

The membrane morphologies were observed using a field−emission scanning electron microscope (FESEM, Hitachi S-4800). The lamellar structure of the membrane was investigated by means of a transmission electron microscopy (TEM, JEOL JEM-F200, Japan). The membrane thickness and surface roughness were analyzed by an atomic force microscope (AFM, JPK Nanowizard 4XP, Bruker, Germany). The interlayer spacing of the membrane was characterized by an X-ray diffractometer (XRD, Rigaku SmartLab), and the nanosheet stacking was analyzed by a 2D wide-angle X-ray diffractometer (2D-WAXD, Rigaku HomeLab) with Cu Kα radiation (λ = 1.5405 Å). The chemical composition and structure of the membrane were characterized by a Fourier transform infrared spectrometer (FTIR, Bruker Optics, VERTEX 70). The element contents were analyzed using an energy-dispersive X-ray spectrometer with an Al Ka X-ray source (XPS, Thermo Scientific), and the XPS etching spectra were measured after Ar ion etching treatment. The Zeta potential of the membrane was determined using a SurPASS electrokinetic analyzer (Anton Paar). An optical contact angle and interface tension meter (KINO SL 200 KB) was employed to test the water and diiodomethane contact angles of the membrane. An electrochemical station (Chenhua CHI660D) was used to measure the linear sweep voltammetry and current-time curves for analyzing the ion transmembrane transport, ion conductance, and membrane potential.

### Evaluation of membrane performance

The performance tests of pressure-driven membrane filtration processes were conducted using a cross-flow filtration setup with a home-made membrane module (Supplementary Fig. 50). The effective filtration area was 6.4 cm^2^. A 5 mM NaCl salt solution as the feed was pumped into the membrane module and a transmembrane pressure of 5 bar was controlled by a regulating valve. The performance tests were performed at the room temperature (20 ± 0.4 °C), and the membrane was pressurized for 60 min prior to the test (Supplementary Fig. 51). The water permeance (*P*_w_, L m^−2^ h^−1^ bar^−1^) and the salt rejection rate (*R*, %) were separately calculated by the following equations:

1 $${P}_{{{{{{\rm{w}}}}}}}=\frac{\Delta V}{A{{\times }}\Delta t{{\times }}\Delta P}$$

2 $$R=\left(1-\frac{{C}_{{{{{{\rm{p}}}}}}}}{{C}_{{{{{{\rm{f}}}}}}}}\right){\times }100\%$$

where Δ*V* is the volume of penetrant solution, *A* is the effective membrane area, Δ*t* is the filtration time, Δ*P* is the transmembrane pressure difference, and *C*_p_ and *C*_f_ are the salt concentrations in the permeate and feed, respectively, which were determined by a conductivity meter (WTW Multi 3420).

The osmosis-driven membrane performance tests were carried out using a self-established bench-scale apparatus with a two-compartment module (Supplementary Fig. 52) at room temperature (20 ± 0.4 °C). The membrane with an effective area of 3.14 cm^2^ was mounted in the module to separate two compartments, and the membrane surface faced the feed side. The same volumes (0.5 L) of 0.5 M NaCl solution and DI water were respectively utilized as the draw and feed solution, which are pumped by peristaltic pumps into the different compartments and then flow back into the respective reservoirs. The mass of permeated water was recorded by an electronic balance and the salt concentration was measured via electrical conductivity. The water flux ( *J*_w_, L m^−2^ h^−1^) and salt permeation flux ( *J*_s_, g m^−2^ h^−1^) were respectively calculated by the following equations:

3 $${J}_{{{{{{\rm{w}}}}}}}=\frac{\Delta m}{A{{\times }}\Delta t{{\times }}{\rho }_{{{{{{\rm{w}}}}}}}}$$

4 $${J}_{{{{{{\rm{s}}}}}}}=\frac{{C}_{t}{V}_{t}-{C}_{0}{V}_{0}}{A{{\times }}\Delta t}$$

where Δ*m* is the mass of permeated water, *ρ*_w_ is the density of water, *C*_0_ and *C*_*t*_ are the salt concentrations of the feed side at the initial time and the operating time of *t*, respectively, and *V*_0_ and *V*_*t*_ are the feed solution volumes at the initial time and the operating time of *t*.

The salt-water ratio is defined as *J*_s_/*J*_w_, and the salt rejection rate (*R*, %) was calculated by:

5 $$R=\left({C}_{{{{{{\rm{f}}}}}}}-\frac{{J}_{{{{{{\rm{s}}}}}}}}{{M}_{{{{{{\rm{NaCl}}}}}}}{{\times }}{J}_{{{{{{\rm{w}}}}}}}}\right) \, / \, {C}_{{{{{{\rm{f}}}}}}}{{\times }}100\%$$

where *C*_f_ is the salt concentration of the draw solution (0.5 M), *M*_NaCl_ is the molecular weight of NaCl.

### Calculations and simulations

The transmembrane energy barriers of ion transports were determined by the Arrhenius approach. The spatial distributions of ions in rGO and ArGO-PSSNa membrane nanochannels were modeled using finite element software COMSOL Multiphysics (version 5.4). The MD simulations of water desalination through rGO and ArGO-PSSNa nanochannels were conducted using a large-scale atomic/molecular massive parallel simulator (LAMMPS) software package (version 29 OCT 2020). The DFT calculations of the electrostatic attractive ion-membrane interactions were performed using the DMol3 code in the Materials Studio software package (version 8.0). The detailed descriptions of these calculations and simulations are provided in the supplementary information.

### Supplementary information

Supplementary Information

Peer Review File

### Source data

Source Data

## Data Availability

The data that supports the findings of the study are included in the main text and supplementary information files. Raw data can be obtained from the corresponding author upon request. Source data have been deposited in Figshare^55^, 10.6084/m9.figshare.25658928. Source data are provided with this paper.
