# Supplementary material for: Electrostatic-induced ion-confined partitioning in graphene nanolaminate membrane for breaking anion–cation co-transport to enhance desalination
Source: Nat Commun. 2024 May 21;15:4324. doi: 10.1038/s41467-024-48681-8 (PMC11109394; doi:10.1038/s41467-024-48681-8)
Supplement: Supplementary file 1 — Supplementary Information [file 41467_2024_48681_MOESM1_ESM.pdf]

Supplementary Information

Electrostatic-Induced Ion Confined Partitioning in Graphene Nanolaminate Membrane for Breaking Anion-Cation Co-Transport to Enhance Desalination

Haiguang Zhang[1,2], Jiajian Xing[1,2], Gaoliang Wei[1], Xu Wang[1], Shuo Chen[1], Xie Quan*[1]

[1]Key Laboratory of Industrial Ecology and Environmental Engineering (Ministry of Education, China), School of Environmental Science and Technology, Dalian University of Technology, Dalian 116024, China.

[2]These authors contributed equally: Haiguang Zhang, Jiajian Xing.

*Corresponding Author

School of Environmental Science and Technology, Dalian University of Technology, Dalian, China

Phone: +86-411-84706140. Fax: +86-411-84706263. E-mail: quanxie@dlut.edu.cn.

This Supplementary Information includes:

Pages (including the cover): 1 to 61

Supplementary Figures: 1 to 52

Supplementary Tables: 1 to 8

Supplementary Notes: 1 to 16

Supplementary References

Supplementary Note 1.

Materials and chemicals

Natural graphite powder (2000 mesh), m-phenylenediamine and trimesoyl chloride were purchased from Aladdin Chemistry Co., Ltd. (Shanghai, China). Hydrophilic modified polyvinylidene difluoride (PVDF) membranes with the diameter of 47 mm and average pore size of 0.1 μm were provided by Merck Millipore Co., Ltd. (Shanghai, China). Polystyrenesulfonate (PSS, Mw = 70,000 g mol^{-1}) was supplied by Macklin Biochemical Co. Ltd., (Shanghai, China). Ammonia solution (25% in water) hydrazine hydrate (80 wt.%) and n-hexane were purchased from Tianjin Fuyu Fine Chemical Co., Ltd. (Tianjin, China). Sodium chloride (NaCl, 99.5%), potassium chloride (KCl, 99.5%), sodium sulfate (Na_2SO_4, 99%), magnesium sulfate ($MgSO_4$, 99%) and other chemicals and reagents were purchased from Tianjin Damao Chemical reagents factory (Tianjin, China), which were analytical grade without further purification. Ultrapure water (18 MΩ cm) was purified by the Milli-Q water purification system (Bedford, USA).

Morphologies of ArGO-PSSNa membrane

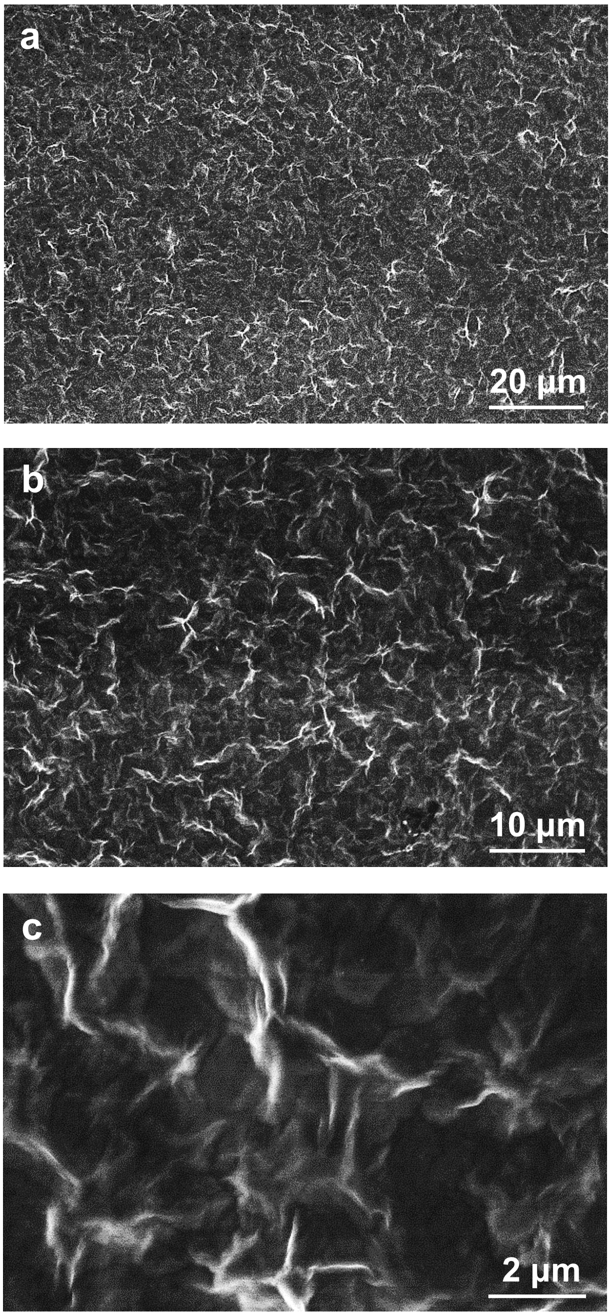

Supplementary Fig. 1. SEM characterization of ArGO-PSSNa membrane. a-c, SEM images of the surfaces of ArGO-PSSNa membrane with different magnifications.

Atomic force microscopy (AFM) characterization

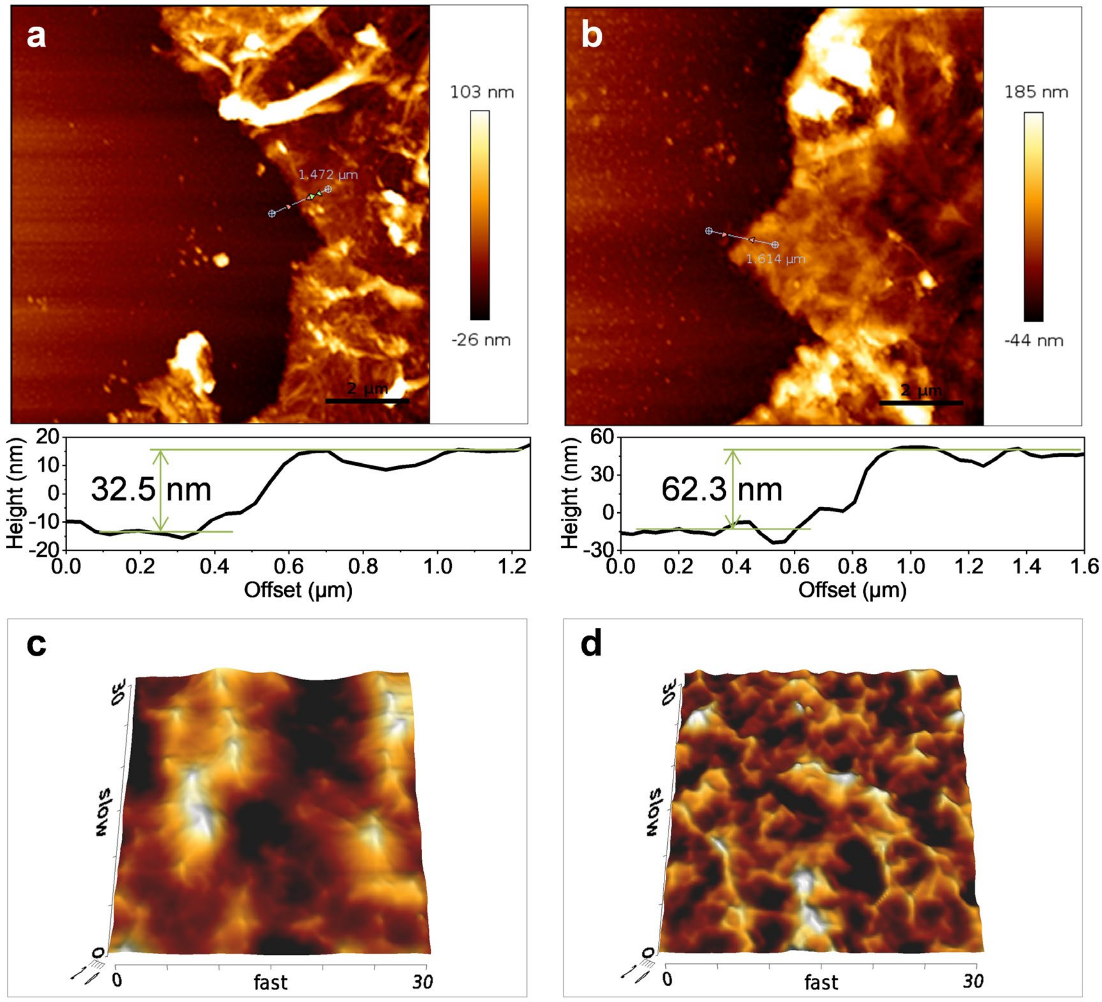

Supplementary Fig. 2. AFM characterization of rGO and ArGO-PSSNa membranes. a,**b**, AFM images and cross-section analyses for rGO and ArGO-PSSNa membranes. **c**,**d**, Three-dimensional views of the surfaces of rGO and ArGO-PSSNa membranes. Image size is 30 × 30 µm^2.

Morphologies of GO and ArGO membranes

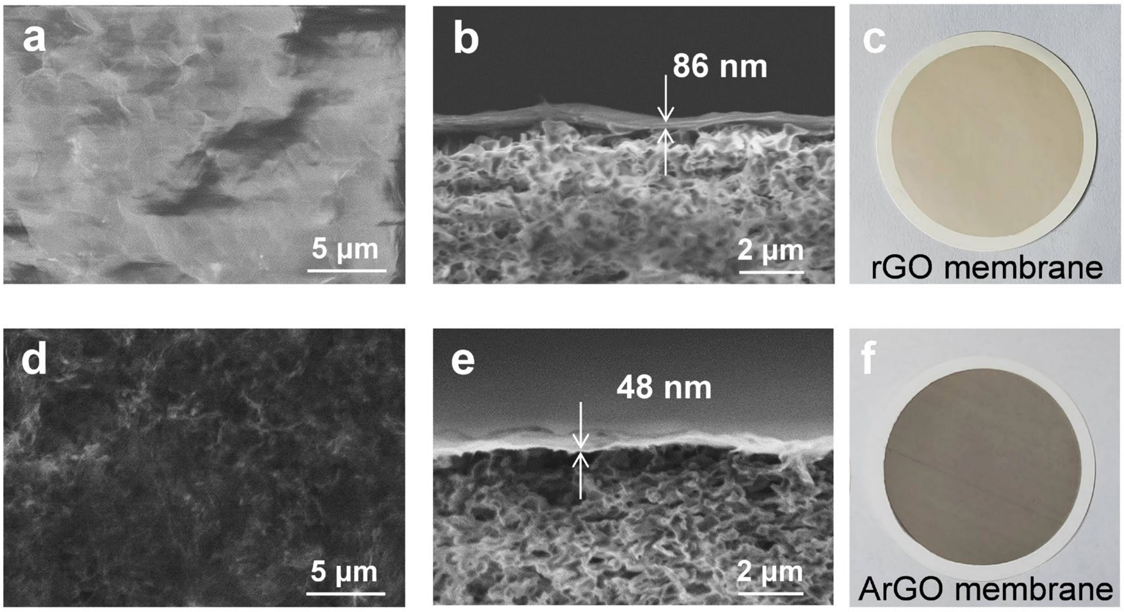

Supplementary Fig. 3. Micro and macro morphologies of GO and ArGO membranes. a,**b**, Top and cross-section views of GO membrane. **c**, Digital photograph of GO membrane. **d**,**e**, Top and cross-section views of ArGO membrane. **f**, Digital photograph of ArGO membrane.

Energy-dispersive x-ray spectroscopy (EDS) characterization

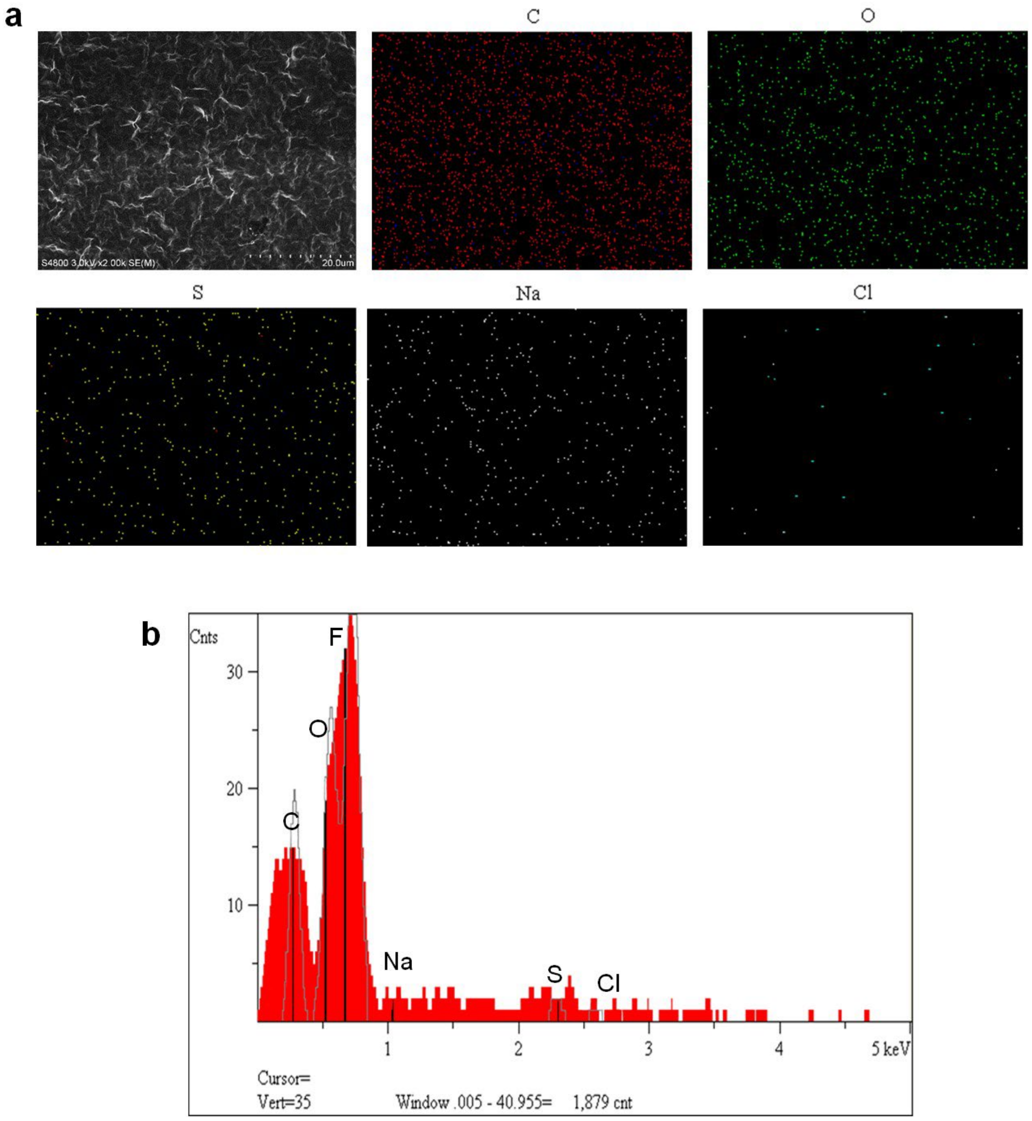

Supplementary Fig. 4. EDS characterization of ArGO-PSSNa membrane. a, EDS mapping images of ArGO-PSSNa membrane. **b**, Element distributions of ArGO-PSSNa membrane.

X-ray photoelectron spectroscopy (XPS) characterization and analysis

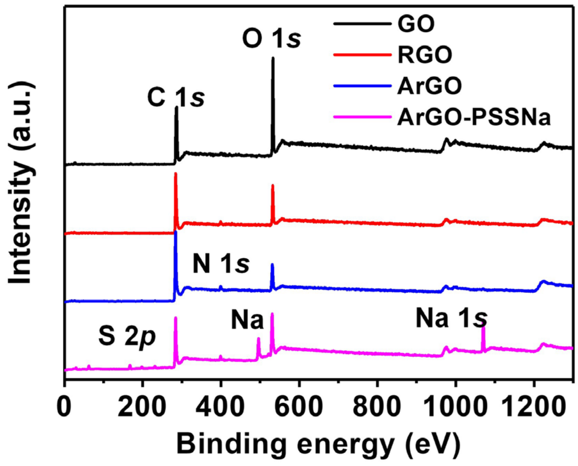

Supplementary Fig. 5. XPS characterization of membranes. XPS spectra of GO, rGO, ArGO and ArGO-PSSNa membranes.

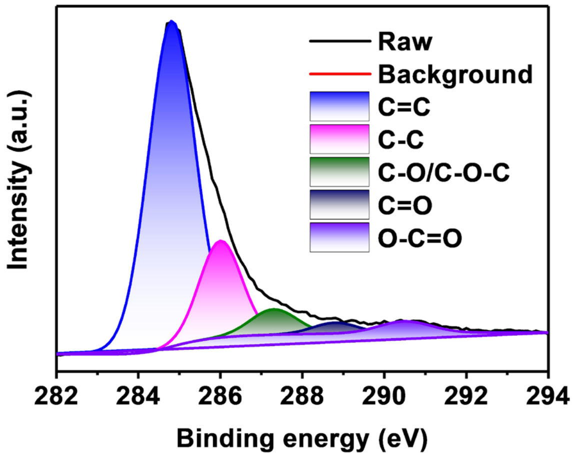

Supplementary Fig. 6. Deconvolution of XPS C 1*s* spectra. Deconvoluted XPS C 1*s* spectra of rGO membrane.

Supplementary Table 1. Functional groups resulting from curve fitting of C 1*s* spectra (%) in Supplementary Fig. 6.

Groups	Content (%)
C=C sp^2	72.1
C−C sp^3	17.5
C−O/C−O−C	5.4
C=O	2.2
O−C=O	2.8

Zeta potential characterization

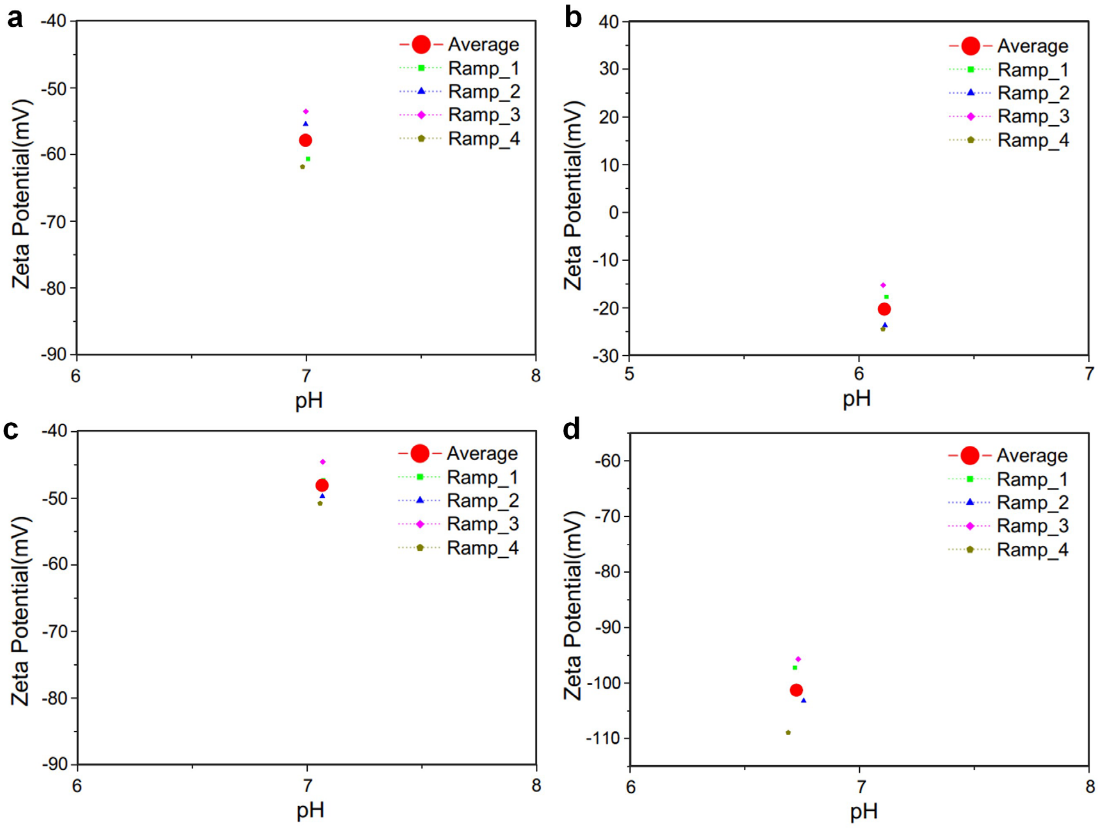

Supplementary Fig. 7 Zeta potential characterization of membranes. Zeta potentials of (**a**) GO, (**b**) rGO, (**c**) ArGO and (**d**) ArGO-PSSNa membranes.

Supplementary Note 2.

The Zeta potentials of ArGO-PSSNa membrane at various pH values from 3 to 11 are shown in Supplementary Fig. 8. The membrane exhibits negative Zeta potentials in the range of pH 3-11, and the Zeta potential gradually becomes more negative as the pH value increases. Notably, there is only a ~10 mV change of Zeta potential in the range of pH 3-11. Generally, the carboxylic groups on the surface of rGO nanosheets can be ionized, making the Zeta potential highly dependent on pH value[1]. Whereas, the PSSNa in ArGO-PSSNa membrane is more likely to form dissociated ionization state, having little influence on Zeta potential in the range of pH 3-11. Thus, it could be inferred that the embeddedness of PSSNa can significantly enhance the negatively charged property of the ArGO-PSSNa membrane.

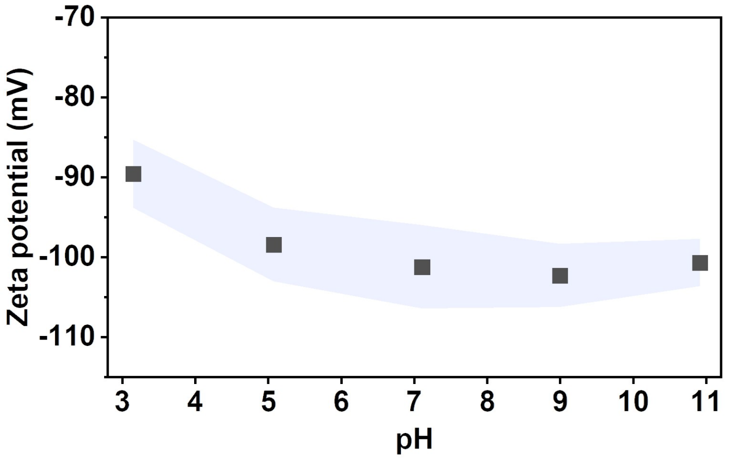

Supplementary Fig. 8. Variation of Zeta potential over pH value. Zeta potentials of the ArGO-PSSNa membrane at various pH values from 3 to 11. The light blue region represents the standard deviation of three replicate measurements.

Membrane stability

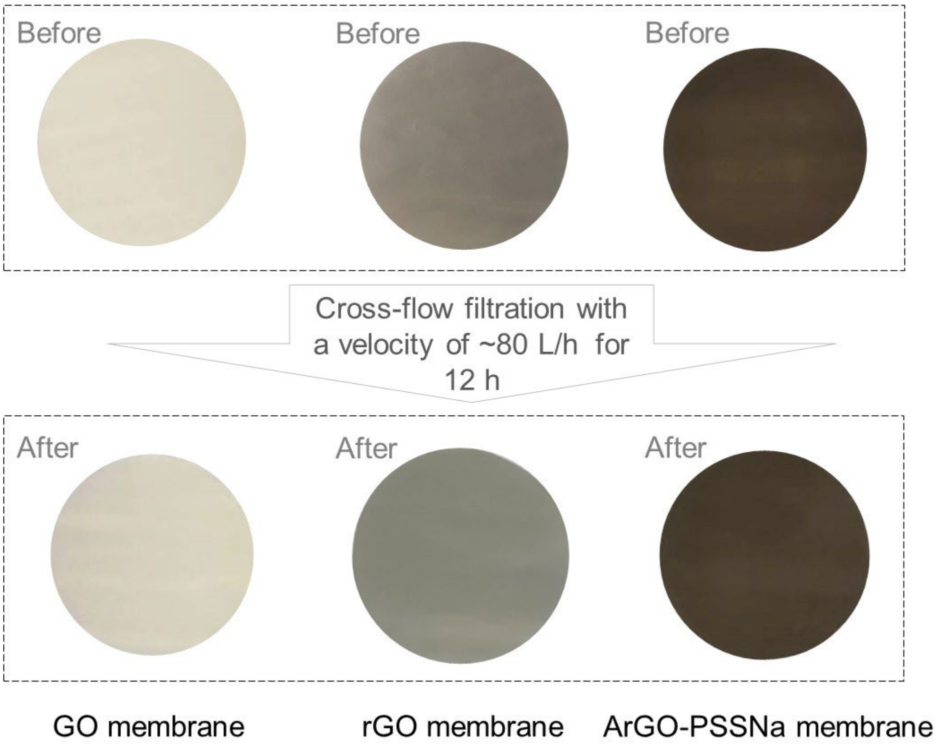

Supplementary Fig. 9. Cross-flow filtration stability of membranes. Digital images of GO, rGO and ArGO-PSSNa membranes before and after filtration under a cross-flow velocity of 80 L h^{-1} (corresponding to a cross-flow velocity of ~37 cm s^{-1}).

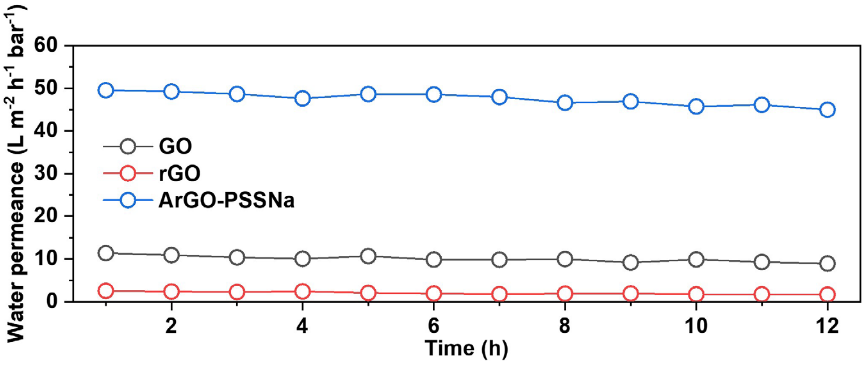

Supplementary Fig. 10. Water permeance during the cross-flow filtration process. Time variations of water permeances of GO, rGO and ArGO-PSSNa membranes. The lines between symbols are guides to the eye.

Supplementary Note 3.

Analysis of water permeation in two-dimensional nanolaminate membrane

Two-dimensional (2D) nanolaminate membranes rely on the interlayer nanochannels formed by nanosheets as the membrane pores for water transport. The 2D nanolaminate membrane with a thickness of h possesses an interlayer distance d, including the thickness a of nanosheet with electronic clouds and the "empty" interlayer space δ (effective nanochannel width). The tortuous path of water permeation through the nanolaminate membrane involves the number of turns N ($N=h/d$), and the transport length between two adjacent turns is L (we assume that the membrane is homogeneous and has the same capillary length L). Therefore, the total length l of water permeation is given by $N\times L$.

According to the Hagen-Poiseuille equation, the mass flow per unit area of water inside the nanochannels can be described as[2]:

$$Q \approx \delta^3 \left(\frac{1}{12\eta}\right)\left(\frac{1}{L}\right)\left(\frac{\Delta P}{l}\right)\rho \quad (1)$$

where Q is mass flow per unit area of water, η is the viscosity of water, ΔP is the transmembrane pressure, and ρ is the density of water. Obviously, the water flux (mass flow per unit area, Q) is proportional to the third power of the interlayer space (effective nanochannel width, δ). In this work, when the membrane interlayer spacing enlarges from 0.94-0.97 nm to 1.28 nm, the water permeance is increased by approximately 2.3-2.5 times based on the Equation (1).

Supplementary Note 4.

<u>Dynamic contact angle measurements and surface free energy calculations</u>

The dynamic water contact angles of the GO, rGO, ArGO and ArGO-PSSNa membranes were tested using an optical contact angle and interface tension meter (SL200KB, KINO Industry CO. Ltd., USA). During the dynamic water contact measurement, a water droplet with the volume of approximately 2 μL was dropped on the membrane surface from a flat-tipped stainless steel needle. When the water droplet touches the surface of the membrane, a high-speed camera was employed to monitor the water contact angle and record the variation of water contact angle over time. Supplementary Fig. 11 shows the digital photographs of the water contact angles at 0 and 3.5 min. Obviously, all of the water contact angles of GO, rGO, ArGO and ArGO-PSSNa membranes are declined after 3.5 min, demonstrating that water molecules can spontaneously enter the membrane nanochannels due to the water wettability and capillarity effects. Especially, the ArGO-PSSNa membrane shows the most significant change in contact angle, and the water droplet almost disappears on the membrane surface, suggesting that the embeddedness of PSSNa enhances the water wettability of the membrane, which could reduce water transport resistance in membrane channels.

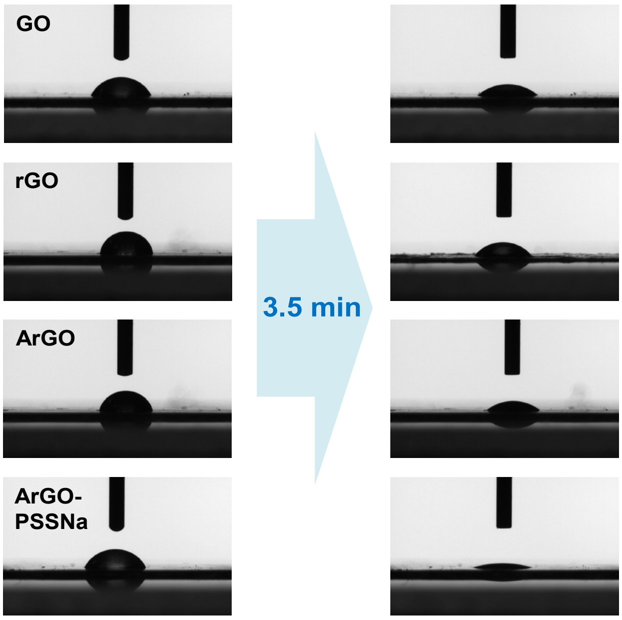

Supplementary Fig. 11. Variation of dynamic water contact angle. Digital photographs of dynamic water contact angles of GO, rGO, ArGO and ArGO-PSSNa membranes before and after 3.5 min.

The surface free energy (SFE) of the membrane was calculated using the Owens–Wendt–Rabel–Kaelble (OWRK) method based on the experimentally measured contact angle values[3-5]. In the OWRK model, the surface free energy can be discriminated in terms of two kinds of forces: the dispersive force (van der Waals interactions) and the polar force (dipole-dipole interactions and hydrogen bonds). The polar and dispersive components of the surface energy are determined by this method, as follows:

$$\gamma_{\mathrm{lv}}(1+\cos\theta)=2\left(\sqrt{\gamma_{\mathrm{sv}}^{\mathrm{d}}\gamma_{\mathrm{lv}}^{\mathrm{d}}}+\sqrt{\gamma_{\mathrm{sv}}^{\mathrm{p}}\gamma_{\mathrm{lv}}^{\mathrm{p}}}\right) \tag{2}$$

where γ_{lv} and γ_{sv} is the free energies (mJ m^{-2}) of the liquid and solid against the saturated vapor of the liquid, respectively, θ is the contact angle (°), $\gamma_{\mathrm{sv}}^{\mathrm{d}}$ and $\gamma_{\mathrm{sv}}^{\mathrm{p}}$ are separately the dispersive and polar components of the free energy of solid (mJ m^{-2}), $\gamma_{\mathrm{lv}}^{\mathrm{d}}$ and $\gamma_{\mathrm{lv}}^{\mathrm{p}}$ represent the dispersive and polar components of the free energy of liquid (mJ m^{-2}), respectively. In the Equation (2), γ_{lv}, $\gamma_{\mathrm{lv}}^{\mathrm{d}}$ and $\gamma_{\mathrm{lv}}^{\mathrm{p}}$ are known parameters for specific liquids, while $\gamma_{\mathrm{sv}}^{\mathrm{d}}$ and $\gamma_{\mathrm{sv}}^{\mathrm{p}}$ are two unknowns. Thus, it is necessary to measure the contact angles of two liquids (different polarity) with the known dispersive and polar components for solve the Equation (2). In this study, ultrapure water and diiodomethane were employed to evaluate the SFE of membrane. The γ_{lv}, $\gamma_{\mathrm{lv}}^{\mathrm{d}}$ and $\gamma_{\mathrm{lv}}^{\mathrm{p}}$ of ultrapure water and diiodomethane are listed in Supplementary Table 2, and the diiodomethane contact angles of GO, rGO, ArGO and ArGO-PSSNa membranes are shown in Supplementary Fig. 12.

Supplementary Table 2. Free energy, dispersive and polar components of water and diiodomethane.

Liquid	γ_{lv} (mJ m^{-2})	$\gamma_{\mathrm{lv}}^{\mathrm{d}}$ (mJ m^{-2})	$\gamma_{\mathrm{lv}}^{\mathrm{p}}$ (mJ m^{-2})
Water	72.8	21.8	51
Diiodomethane	50.8	50.8	0

According to Equation (2), the $\gamma_{\mathrm{sv}}^{\mathrm{d}}$ and $\gamma_{\mathrm{sv}}^{\mathrm{p}}$ can be expressed as:

$$\left(\gamma_{\mathrm{sv}}^{\mathrm{d}}\right)^{0.5}=\frac{\gamma_{\mathrm{d}}(1+\cos\theta_{\mathrm{d}})-\sqrt{\left(\gamma_{\mathrm{d}}^{\mathrm{p}}/\gamma_{\mathrm{w}}^{\mathrm{p}}\right)}\cdot\gamma_{\mathrm{w}}(1+\cos\theta_{\mathrm{w}})}{2\left(\sqrt{\gamma_{\mathrm{d}}^{\mathrm{d}}}-\sqrt{\gamma_{\mathrm{d}}^{\mathrm{p}}\left(\gamma_{\mathrm{w}}^{\mathrm{d}}/\gamma_{\mathrm{w}}^{\mathrm{p}}\right)}\right)} \tag{3}$$

$$\left(\gamma_{\mathrm{sv}}^{\mathrm{p}}\right)^{0.5}=\frac{\gamma_{\mathrm{w}}\left(1+\cos\theta_{\mathrm{w}}\right)-2\sqrt{\left(\gamma_{\mathrm{sv}}^{\mathrm{d}}\gamma_{\mathrm{w}}^{\mathrm{d}}\right)}}{2\sqrt{\gamma_{\mathrm{w}}^{\mathrm{p}}}} \tag{4}$$

The total surface free energy (SFE) of solid is calculated by:

$$SFE=\gamma_{\mathrm{sv}}=\gamma_{\mathrm{sv}}^{\mathrm{d}}+\gamma_{\mathrm{sv}}^{\mathrm{p}} \tag{5}$$

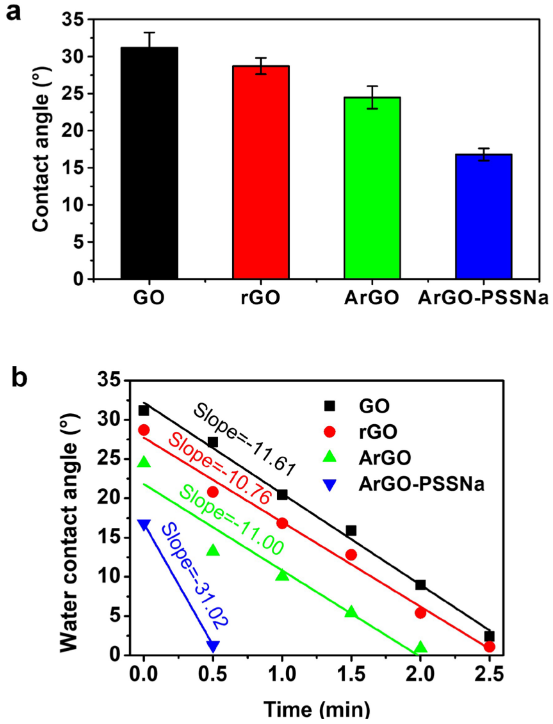

Supplementary Fig. 12. Diiodomethane contact angle measurements of membranes. a, Diiodomethane contact angles of GO, rGO, ArGO and ArGO-PSSNa membranes. **b**, Time variations of diiodomethane contact angles for GO, rGO, ArGO and ArGO-PSSNa membranes. The lines denote the linear fits made with the numerical model. Diiodomethane serves as the test solution. Error bars represent the standard deviation of three replicate measurements.

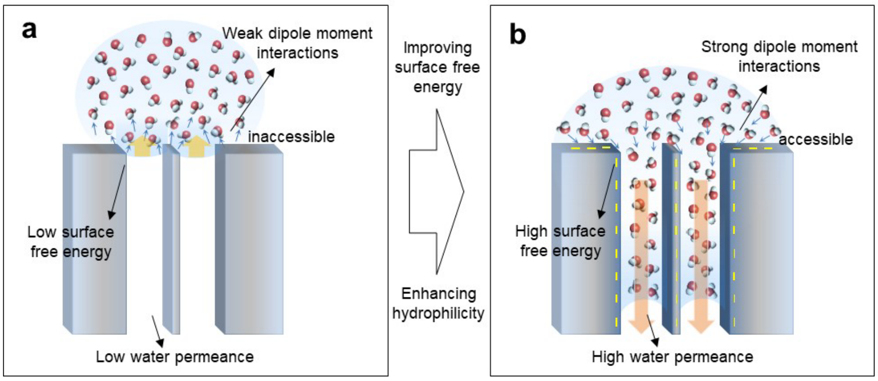

Supplementary Fig. 13. Interactions between water molecules and the membrane nanochannels. a, Schematic diagram of the interactions between water molecules and the membrane nanochannels with low surface free energy. **b**, Schematic diagram of the interactions between water molecules and the membrane nanochannels with high surface free energy.

Fouling propensity of membranes

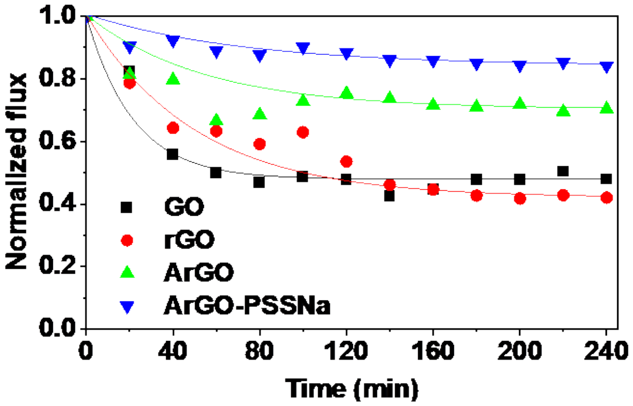

Supplementary Fig. 14. Testing of fouling properties of membranes. Normalized water fluxes of GO, rGO, ArGO and ArGO-PSSNa membranes for filtering 0.2 g L^{-1} BSA feed solution. The lines between symbols are guides to the eye.

Pressure-driven membrane desalination stability

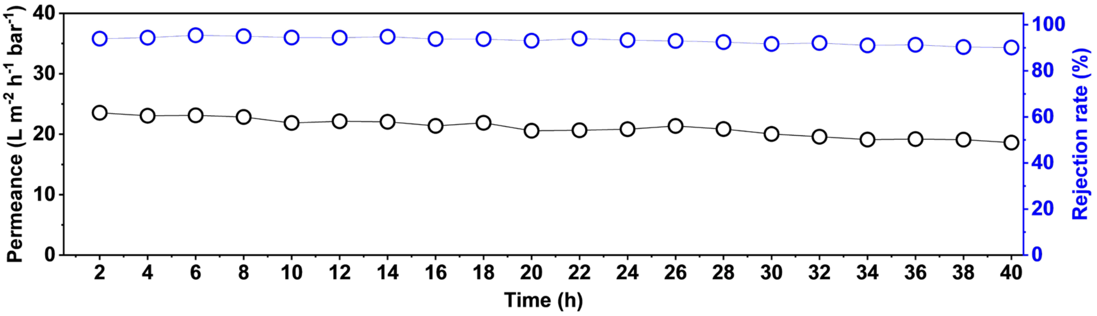

Supplementary Fig. 15. Pressure-driven desalination stability of ArGO-PSSNa membrane. Water permeance and NaCl (5 mM) rejection rate of pressure-driven ArGO-PSSNa membrane under a transmembrane pressure of 5 bar during operation of 40 h. The lines between symbols are guides to the eye.

Effect of salt on membrane performance

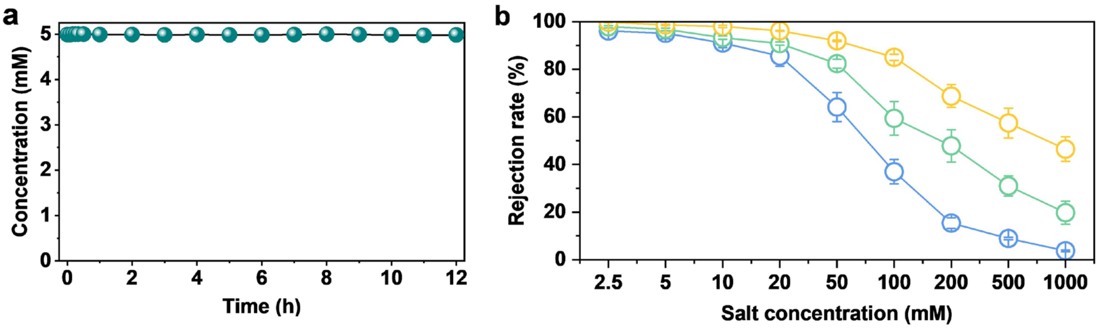

Supplementary Fig. 16. Salt adsorption and salt rejection for different concentrations. a, Time variation of NaCl concentration after putting a ArGO-PSSNa membrane into 1 L 5 mM NaCl feed solution. **b**, NaCl rejection rates of ArGO-PSSNa membranes for different NaCl feed concentrations from 2.5 mM to 1000 mM. The ArGO-PSSNa1 membrane was prepared using 4 mL 0.05 mg mL^{-1} rGO dispersion, 5 mL 1.0 wt.% PSSNa (in 1 M NaCl solution) and 5 mL 2.0 wt.% m-phenylenediamine; the ArGO-PSSNa2 membrane was prepared using 4 mL 0.05 mg mL^{-1} rGO dispersion, 5 mL 2.0 wt.% PSSNa (in 1 M NaCl solution) and 5 mL 3.0 wt.% m-phenylenediamine; the ArGO-PSSNa3 membrane was prepared using 4 mL 0.05 mg mL^{-1} rGO dispersion, 5 mL 3.0 wt.% PSSNa (in 1 M NaCl solution) and 5 mL 4.0 wt.% m-phenylenediamine. Error bars represent the standard deviation of three replicate measurements. The lines between symbols are guides to the eye.

Supplementary Table 3. The performances of the ArGO-PSSNa membrane and some reported graphene based membranes as well as commercial membranes.

Materials	Pressure (bar)	NaCl feed	Permeance (L m^{-2} h^{-1} bar^{-1})	NaCl rejection (%)	Ref.
GO/TMC	3.4	20 mM	~50	19	6
Highly laminated GO	2	1 M	4	25	7
GO/FLG membrane	50	40 mM	0.34 (± 0.1)	85	8
GO	1.5	20 mM	16.94	50.1	9
GO membranes in EPR system	2	0.5 g L^{-1}	12.5	97	10
$Ti_3C_2T_x$-GO membrane	5	0.1 M	2.25	~11	11
Nematic multi-layered GOMs	0.5	2 g L^{-1}	71	30–40	12
MWCNT/rGO membrane	5	10 mM	11.3	51.4	13
cross-linked TA-GO membrane	1	1 g L^{-1}	15.4	66	14
rNPGO membrane	6	20 mM	39.93	40	15
KCl-treated GOMs	–	0.25 M	0.36 ±0.06	94.7	16
(PEI/GO/PEI)/hPAN	5	1 g L^{-1}	4.2	38.1	17
GOLM-100-6/30	8	2 g L^{-1}	~1	~30	18
activated graphene oxide (AGO)	5	0.2 M	36.5	20.45	19
rGO-CNT hybrid nanofiltration	3	5 mM	28	42	20
rGOMs	6	1 g L^{-1}	1.05	83	21
TCPP–rGO-0.7 membrane	10	0.5 g L^{-1}	~1	95	22
GO–GRGDS–AQP membrane	8	1 g L^{-1}	7.83	99.1	23

TU-GOF membrane	5	0.1 g L^{-1}	1.5	95.6	24
GO–TBO membrane	10	10 mM	0.5-1	85	25
rGO/MoS2 Membrane	1	5 mM	10	87.2	26
sCPF membrane	4	10 mM	9.5	83	27
Glc10T60	10	0.5 g L^{-1}	0.1	>90	28
GO-CD-PIP	6	1 g L^{-1}	18.3	74	29
uGNM	5	20 mM	21.8	~42	30
GO membrane	10	2 g L^{-1}	~6	35	31
GO–graphene membrane	6	1 g L^{-1}	6	88.3	32
PrGO6-50	8	0.5 g L^{-1}	4.2	42.4	33
BPEI/GO_TU	4	0.5 g L^{-1}	6	43	34
GO@nylon 6–16	1	20 mM	11.15	27.6	35
		5 mM		95.5	
ArGO-PSSNa membrane	5	10 mM	48.6	91.0	This work
		20 mM		85.6	

Supplementary Note 5.

Rejection performance of other salts and PEG

Supplementary Fig. 17 shows the rejection performance of Na_2SO_4, $MgSO_4$, KCl and $MgCl_2$ salts and neutral polyethylene glycol (PEG) molecules with different molecular weights (200, 400, 600, 1000 and 2000 Da). The rejection rates of ArGO-PSSNa membrane for bivalent Na_2SO_4 and $MgSO_4$ reach more than 96%, and the rejection of KCl and $MgCl_2$ can be maintained above 90%. This result suggests that the membrane shows higher rejection rate for the salt with higher ion valence ratio of anion to cation. For the neutral PEG rejection results, it can be seen that the membrane can completely reject the PEG molecules larger than 1000 Da, and the rejection rate is gradually declined with the decrease of PEG molecular weight. The ArGO-PSSNa membrane shows a rejection of 90% (defined as molecular weight cut-off (MWCO)) at a PEG molecular weight of approximately 600 Da, which is in the category of molecular weight (200-1000 Da) of nanofiltration membrane.

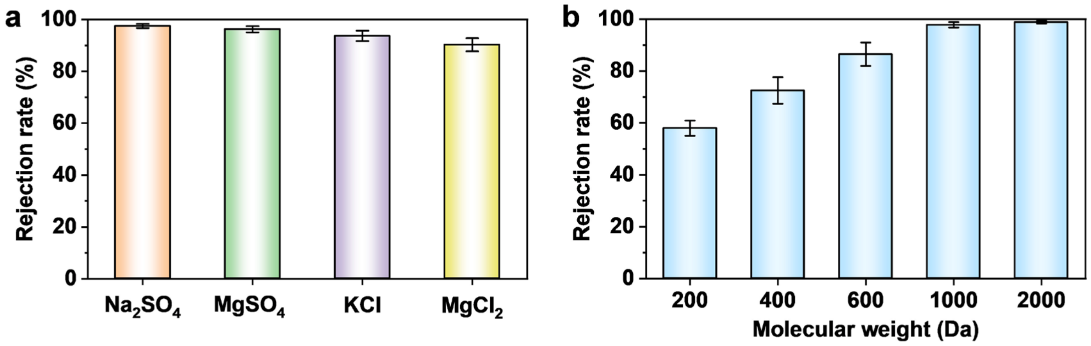

Supplementary Fig. 17. Rejection performance of ArGO-PSSNa membrane for salts and PEG molecules. **a**, Rejection rates of ArGO-PSSNa membrane at 5 bar for different salts (Na_2SO_4, $MgSO_4$, KCl, $MgCl_2$) solutions with the concentration of 5 mM. **b**, Rejection rates of ArGO-PSSNa membrane for 0.5 g L^{-1} neutral PEG molecules with different molecular weight from 200 to 2000 Da. Error bars represent the standard deviation of three replicate measurements.

Supplementary Note 6.

<u>Electrochemical tests of ion transport through the membrane</u>

The ion transmembrane transport was investigated by an electrochemical method. A home-made two-compartment cell was used, and the membrane was placed between the two compartments (Supplementary Fig. 18). A 5 mM NaCl solution and deionized water were added in the two compartments. An Ag/AgCl electrode as the working electrode was put into the deionized water, and the other one as the counter electrode was placed in the 5 mM NaCl solution. The electrochemical current-voltage (*I*−*V*) and current-time (*I*–*t*) curves were measured using an electrochemical workstation for investigating the ion transport behavior.

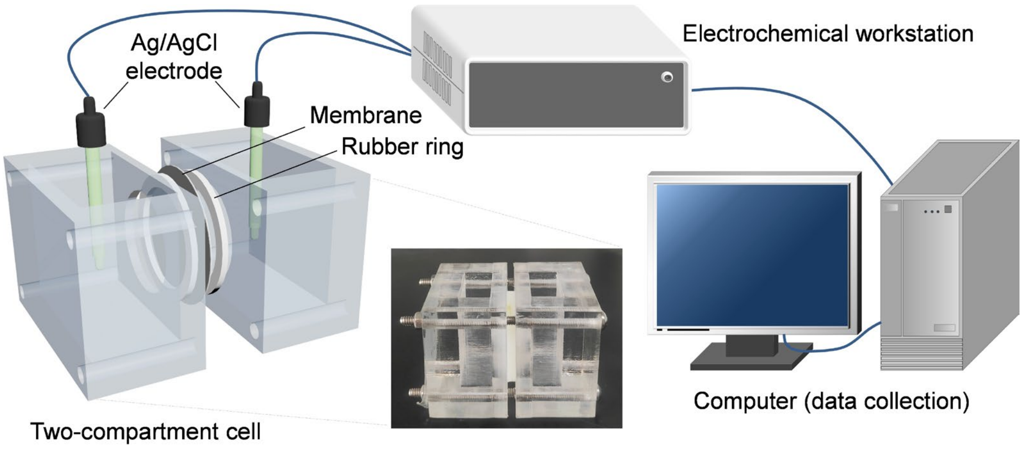

Supplementary Fig. 18. Electrochemical test setup. Schematic diagram of the setup for electrochemical tests of ion transport through the membrane.

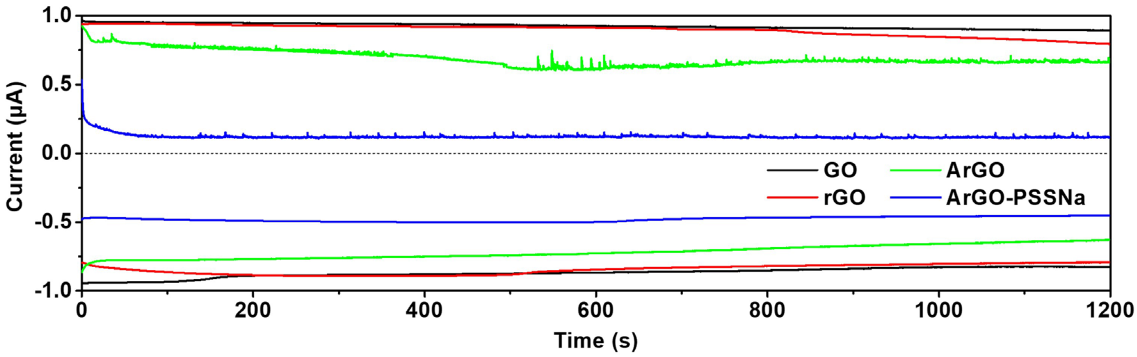

Supplementary Fig. 19. Electrochemical current-time (*I*–*t*) curves of membranes. *I*–*t* curves of GO, rGO, ArGO and ArGO-PSSNa membranes measured in a two-compartment cell filled with a 5 mM NaCl solution and deionized water. The dashed lines are guide to the eye. Two Ag/AgCl electrodes were used for testing the *I*–*t* curves, and the tested voltage is set as 1.0 V.

Supplementary Note 7.

Transmembrane energy barriers for salt permeation

The transmembrane energy barriers of salt permeation through the GO, rGO, ArGO and ArGO-PSSNa membranes were determined by a concentration gradient–driven salt permeation process in a home-made two-compartment diffusion cell. The membrane was placed between the two chambers, and a 5 mM NaCl solution (feed side) and deionized water (permeate side) were added in the two chambers, forming a concentration gradient for driving the salt permeation. Because the PVDF substrate of the composite membrane in this study possesses an average pore size of 0.1 μm that is much larger than the pore size of the membrane function layer, it could be assumed that such a large pore size of the substrate has a negligible effect on the salt transport. By measuring the concentrations of salt permeated through the membrane at different temperatures (293, 303, 313 and 323 K), the temperature-dependent salt permeation fluxes were determined. Based on the salt permeation flux−temperature relationship, the transmembrane energy barrier of salt permeation was calculated according to the Arrhenius-type equation[36,37]:

$$\ln\left(\frac{J_{\mathrm{s}}}{C_{\mathrm{f}}-C_{\mathrm{p}}}\right)=\ln(A)-\frac{E_{\mathrm{salt}}}{R}\cdot\frac{1}{T} \tag{6}$$

where J_s is the salt permeation flux (mol m^{-2} s^{-1}), C_f and C_p are the concentrations of salt in the feed and permeate side (mol L^{-1}), respectively, A is the pre-exponential factor, E_{salt} is the transmembrane energy barrier of salt permeation (kcal mol^{-1}). R is the gas constant (1.985×10^{-3} kcal mol^{-1} K^{-1}), T is the absolute temperature (K).

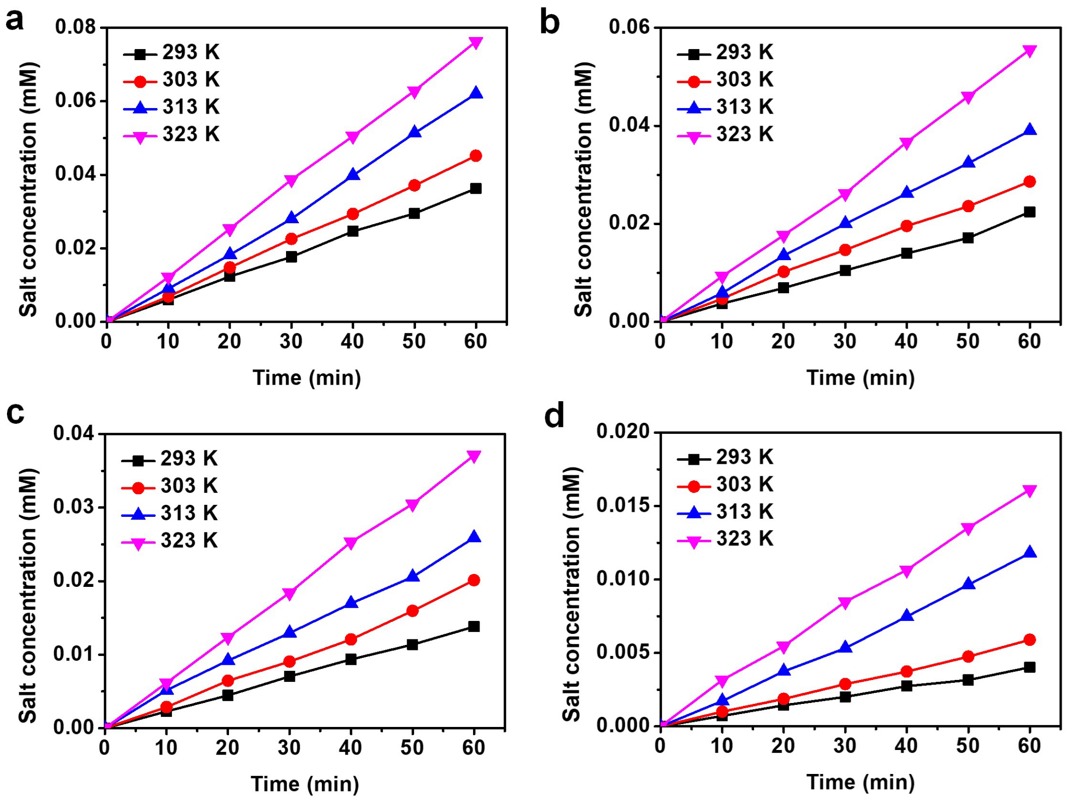

Supplementary Fig. 20. Salt ion permeation through membranes at different temperatures. Time variations of salt concentrations permeated through (**a**) GO, (**b**) rGO, (**c**) ArGO and (**d**) ArGO-PSSNa membranes at different temperatures of 293, 303, 313 and 323 K. The lines between symbols are guides to the eye. The salt permeation tests were conducted in a two-compartment cell filled with a 5 mM NaCl solution and deionized water, and the permeated salt concentration was measured via electrical conductivity.

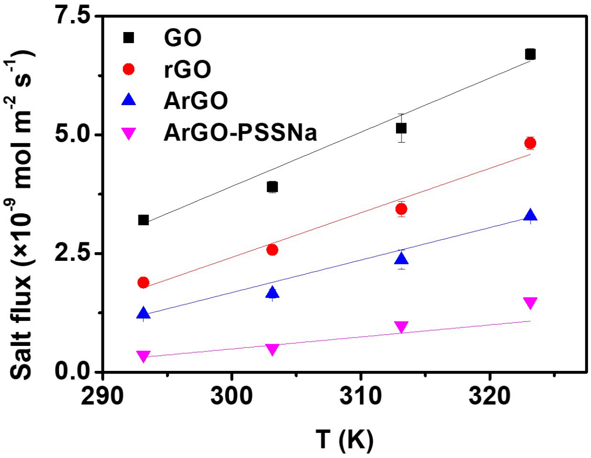

Supplementary Fig. 21. Variation of salt flux of membranes over temperature. Salt fluxes of GO, rGO, ArGO and ArGO-PSSNa membranes for 5 mM NaCl solution as a function of the temperature. Error bars represent the standard deviation of three replicate measurements. The lines denote the linear fits made with the numerical model.

Supplementary Note 8.

COMSOL simulation of ion distribution in the nanochannels

The spatial distributions of cations and anions in the nanochannels of rGO and ArGO-PSSNa membranes were simulated using the finite element software COMSOL Multiphysics (version 5.4)[38,39]. A simplified two-dimensional simulation system including a region of feed and a region of two nanochannels was used for the numerical simulation (Supplementary Fig. 22). According to the interlayer spacings of rGO and ArGO-PSSNa nanochannels in this study, the nanochannel region was set as 1 nm width for modeling the rGO nanochannels (Supplementary Fig. 22a) and 1.3 nm width for modeling the ArGO-PSSNa nanochannels (Supplementary Fig. 22b), and the nanochannel length was 5 nm. The nanochannel surfaces were negatively charged with the surface potential setting as −0.020 V and −0.105 V for rGO and ArGO-PSSNa nanochannels respectively. The feed region was located on the left side of the nanochannels and connected to the nanochannels. and the feed region was set to be 5 nm × 2.76 nm for rGO nanochannel simulation and 5 nm × 3.3 nm for ArGO-PSSNa nanochannel simulation. The concentration of simulated salt solution is 5 mM NaCl. The simulations were performed by coupling two modules of "electrostatics (es)" and "transport of diluted species (tds)", and the stationary solver was used for the calculations.

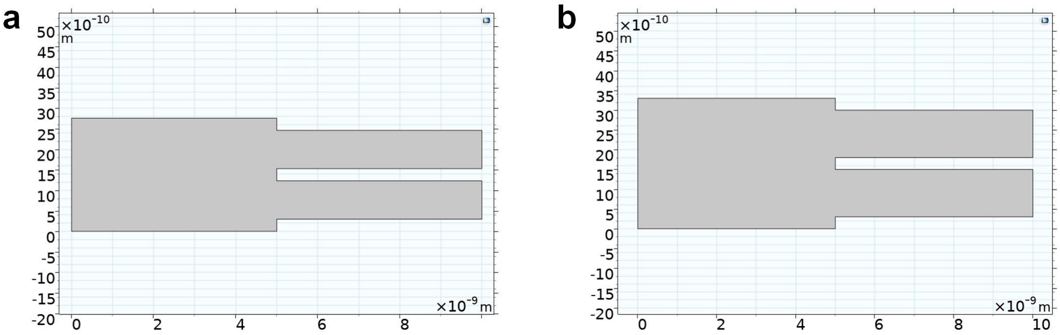

Supplementary Fig. 22. COMSOL simulation configuration. a, COMSOL configurations of rGO nanochannels. For simplicity, the rGO model includes a feed region of 5 nm × 2.76 nm and two rGO nanochannels (formed by three layers of rGO) with the width of 1 nm and length of 5 nm. **b**, COMSOL configurations of ArGO-PSSNa nanochannels. The ArGO-PSSNa model includes a feed region of 5 nm × 3.3 nm and two rGO nanochannels (formed by three layers of ArGO-PSSNa) with the width of 1.3 nm and length of 5 nm. The surfaces of the rGO and ArGO-PSSNa layers are

charged with the surface potentials of −0.020 and −0.105 V, respectively. The simulated salt solution in the feed is set as 5 mM NaCl.

The surfaces of rGO and ArGO-PSSNa nanochannels were negatively charged, which can induce the formation of electric double layer, changing the ion distribution in the nanochannels. Supplementary Fig. 23 shows the calculated electrostatic potential profiles in the feed and the rGO and ArGO-PSSNa nanochannels. Obviously, the electrostatic potential changes more and more negatively from the feed side to the nanochannel, which decreases from 0 to −0.020 V for rGO nanochannels and −0.105 V for ArGO-PSSNa nanochannels. More importantly, the electrostatic potential profiles are almost unchanged in the rGO and ArGO-PSSNa nanochannels, suggesting the overlapped electric double layer in the nanochannels. It can be seen from Supplementary Fig. 23 that the thickness of electric double layer is approximately 2 nm for rGO nanochannels and 4 nm for ArGO-PSSNa nanochannels, which are larger than the width of the nanochannels.

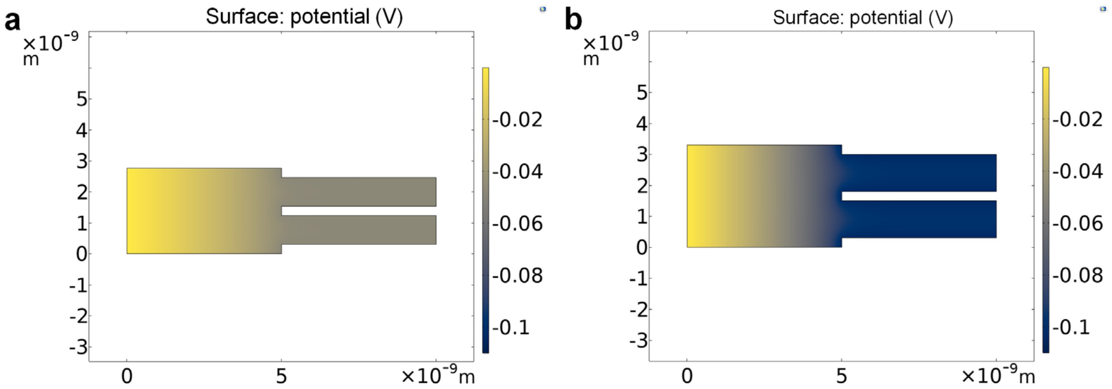

Supplementary Fig. 23. Simulated potential profiles. Variations of simulated potential distribution profiles for (**a**) rGO and (**b**) ArGO-PSSNa nanochannels.

The overlapped electric double layer can lead to the enrichment of cation (Na^+) and the dissipation of anion (Cl^-). Supplementary Figs. 24 and 25 show the cation and anion concentration variations along the direction perpendicular to the rGO and ArGO-PSSNa nanochannels. In the rGO nanochannels, the concentration of Na^+ gradually decreases from 32.5 to 31.9 mol m^{-3} with changing from the nanochannel surface to the center, while the Cl^- concentration gradually increases from 0.76 to 0.78 mol m^{-3} with changing from the surface to the center. Compared with the rGO nanochannels, the ion

concentrations in the ArGO-PSSNa nanochannels show similar variation trend, whereas the ArGO-PSSNa nanochannels possess a higher Na^+ concentration (changing from 300 to 235 mol m^{-3}) and a lower Cl^- concentration (changing from 0.77 to 0.78 mol m^{-3}). Such more significant disequilibrium distributions of cations and anions demonstrate substantially enhanced intra-nanochannel ion partitioning for ArGO-PSSNa nanochannels.

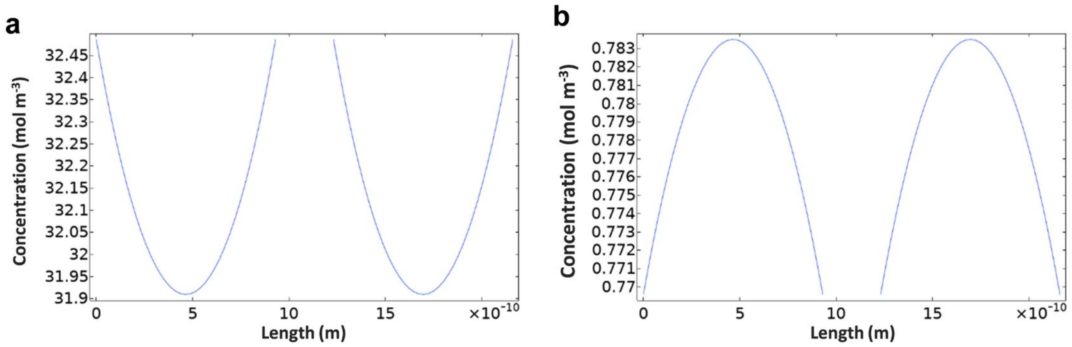

Supplementary Fig. 24. Ion concentration distribution in rGO nanochannels. a, Cation (Na^+) concentration variations along the direction perpendicular to the rGO nanochannels. **b**, Anion (Cl^-) concentration variations along the direction perpendicular to the rGO nanochannels.

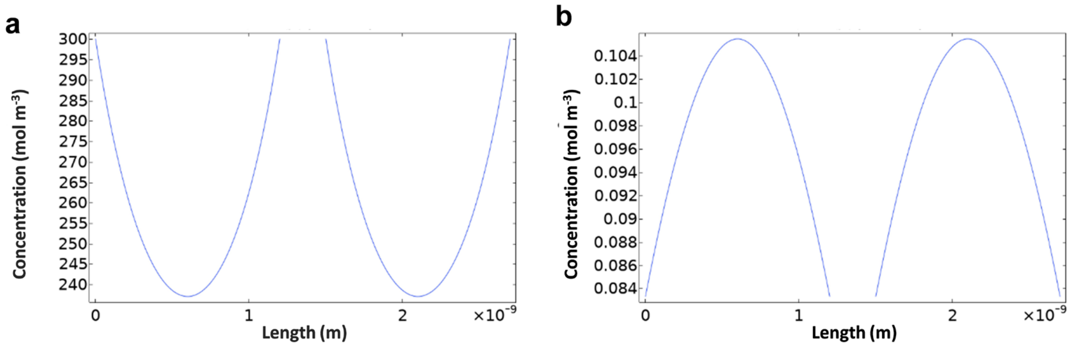

Supplementary Fig. 25. Ion concentration distribution in ArGO-PSSNa nanochannels. a, Cation (Na^+) concentration variations along the direction perpendicular to the ArGO-PSSNa nanochannels. **b**, Anion (Cl^-) concentration variations along the direction perpendicular to the ArGO-PSSNa nanochannels.

Supplementary Note 9.

Electrochemical current changes

The ion rearrangement and partitioning in the membrane were investigated by in-situ monitoring of electrochemical current change. Two platinum electrodes were placed on both sides of the membrane for testing the current. After testing the current for 40 s, salt solution was added for filtration. When the salt solution contacted the membrane, the ions in the membrane can undergo rearrangement, leading to the change of current. The current change for rGO membrane is approximately 0.16 μA (Supplementary Fig. 26), which is lower than that for GO membrane (~0.38 μA). This suggests weak interaction between rGO membrane and ions, implying that fewer ions are rearranged in the membrane so as to result in weak ion partitioning. By comparison, the ArGO-PSSNa membrane shows a current change of 0.82 μA, significantly higher than that of rGO membrane, which demonstrates relatively stronger ion-membrane interaction, implying more ions are rearranged to lead to strong ion partitioning.

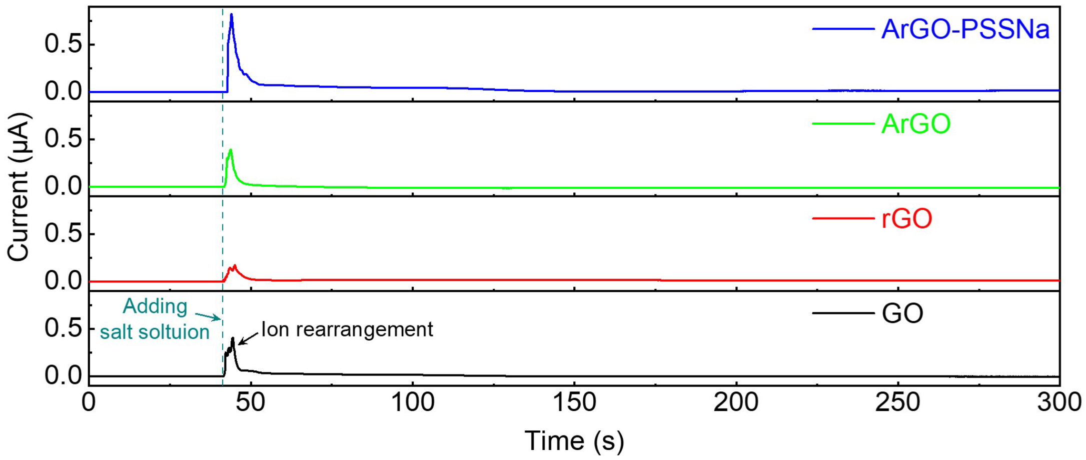

Supplementary Fig. 26. In-situ electrochemical current measurements. Current variations for GO, rGO, ArGO and ArGO-PSSNa membranes before and after adding salt solution for filtration. The dashed lines are guide to the eye.

Supplementary Note 10.

Molecular dynamics (MD) simulations of pressure-driven desalination and ion transport behavior

The desalination performance and the ion transport behavior were investigated by MD simulations. The rGO and ArGO-PSSNa membranes possess nanolamellar structures, and the interlayer nanochannels serve as the membrane pores for water transport and ion separation (Supplementary Fig. 27a,c). Here, two simplified interlayer nanochannels for rGO and ArGO-PSSNa membranes were constructed to simulate the desalination and ion transport. The configurations of rGO and ArGO-PSSNa nanochannels were established using Materials Studio software based on the nanochannel structures of rGO and ArGO-PSSNa membranes. The modeled rGO nanochannel structure included three rGO nanosheets with an area of 71×19 Å^2, and the interlayer spacing (rGO nanochannel width) was set as 10 Å (Supplementary Fig. 27b). Whereas, the ArGO-PSSNa nanochannel structure contains three rGO nanosheets with an area of 71×19 Å^2 to form two nanochannels, and two monomer molecules of sodium polystyrenesulfonate (PSSNa) were placed inside each nanochannels. The interlayer spacing (ArGO-PSSNa nanochannel width) was set as 13 Å (Supplementary Fig. 27d). The feed solution was located at the left of the nanochannels, containing 3277 water molecules, 30 Na^+ ions and 30 Cl^- ions, which corresponded to a NaCl solution of 0.5 M. Such a high concentration of NaCl solution used in this simulation was conducive to inspecting the desalination and ion transport over a relatively short time period. A graphene nanosheet with the size of 86×19 Å^2 served as a piston for providing pressure force to NaCl solution. The MD simulations were performed using the large-scale atomic/molecular massively parallel simulator (LAMMPS)[40-42]. For the LAMMPS simulations, the periodic boundary conditions (PBC) were used on all dimensions. The all-atom optimized potentials for liquid simulations (OPLS-AA) were used for the rGO and ArGO-PSSNa nanochannels, and the extended simple point charge model (SPC/E) was used for water molecules. Both of them included van der Waals and electrostatic terms. The 12−6 Lennard-Jones (L-J) potential

$V(r)=4\varepsilon[(\sigma/r)^{12}-(\sigma/r)^{6}]$ was adopted to calculate the interatomic van der Waals interactions. The characteristic length σ and energy parameter ε were obtained by the common Lorentz-Berthelot combination rule, and the cutoff distance r of the van der Waals interaction was set as 10 Å. The long-range Coulomb interaction was computed by using the particle-particle particle-mesh (PPPM) algorithm. The force field parameters used in the simulation were listed in Supplementary Table 4.

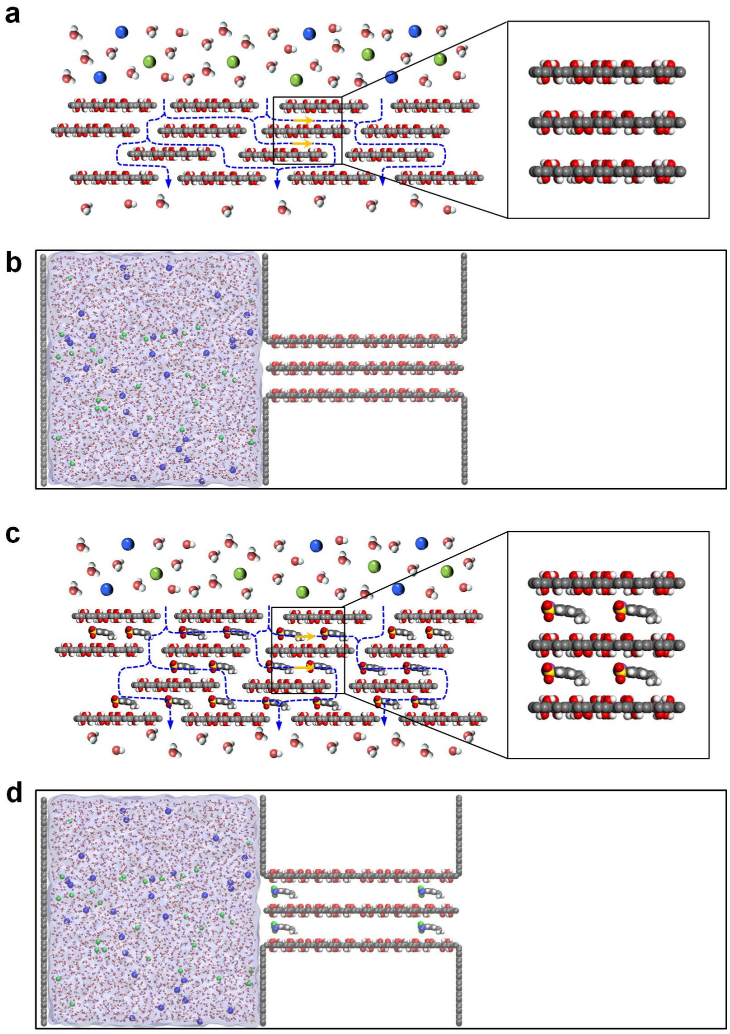

Supplementary Fig. 27. Configurations of rGO and ArGO-PSSNa nanochannels for MD simulation. **a**, Schematic of rGO membrane structure. **b**, Simulation configuration for water desalination through the rGO nanochannels. **c**, Schematic of ArGO-PSSNa membrane structure. **d**, Simulation configuration for water desalination through the ArGO-PSSNa nanochannels. The simulation box is 230×19×86 Å^3. The length of the rGO and ArGO-PSSNa nanochannels is 71 Å, and the widths are 10 Å for rGO nanochannels and 13 Å for ArGO-PSSNa nanochannels. There were 3277 water molecules, 30 Na^+ ions and 30 Cl^- ions in the simulated feed solution. The C, O, H and S atoms are shown in grey, red, white and yellow spheres, separately. Blue and green spheres represent Na^+ and Cl^- ions.

The pressure-driven desalination process was carried out by adding a force to the piston in a nonequilibrium MD simulation. To resolve the dynamics over a relatively short time period, a high pressure of ~100 MPa was applied during the simulation. All of the simulations were conducted in the constant volume and temperature (NVT) ensemble at 300 K controlled by the Nosé-Hoover thermostat method. The time step was set as 1 fs. The simulation system first underwent energy minimization using a steepest descent algorithm, and the equilibrium simulation was then conducted for 100 ps. Subsequently, the simulation was performed for 50 ns for data collection. Such a simulation duration was long enough to obtain the simulation results. The postprocessing was carried out using the Visual Molecular Dynamics (VMD)[40].

Supplementary Table 4. Force field parameters used in the simulation[40,43].

Atom	Mass	ε (kcal/mol)	σ (nm)	q (e)
C (C−C)	12.011	0.105	3.851	0.000
C (C−O)	12.011	0.070	3.550	0.177
O (−OH)	15.999	0.170	3.070	−0.585
H (−OH)	1.008	0.000	0.000	0.408
C (C−H)	12.011	0.066	3.500	−0.299
H (C−H)	1.008	0.015	1.459	0.296
S (S−O)	32.064	0.250	3.550	2.210
O (S−O)	15.999	0.210	2.960	−0.960
O (H_2O)	15.999	0.155	3.166	−0.848
H (H_2O)	1.008	0.000	0.000	0.424
Na^+	22.990	0.130	2.350	1.000
Cl^-	35.453	0.100	4.400	−1.000

Supplementary Fig. 28 shows the number of water molecules, Na^+ and Cl^- ions transported through the rGO and ArGO-PSSNa nanochannels. For both nanochannels, when filtering the same amount of water molecules (1380 water molecules), 4 Na^+ and 4 Cl^- transport through the rGO nanochannels, while there are no ions transporting through the ArGO-PSSNa nanochannels. As seen from the inset of Supplementary Fig.

28a, the Na^+ and Cl^- ions transport through the rGO nanochannels in almost the same amount throughout the MD simulation period, which looks like anion–cation pairs-like formation due to the electroneutrality principle. In contrast, for the ArGO-PSSNa nanochannels, it is difficult for ions to pass through the nanochannels during the whole simulation period. Correspondingly, the mean square displacements (MSD) demonstrate that the diffusion (transport) rate of Na^+ significantly slows down when passing through the ArGO-PSSNa nanochannels (Supplementary Fig. 29). The analyses of the number density distributions of Na^+ and Cl^- ions show that there are low number densities (0.0003-0.0005 atoms nm^{-3}) of Na^+ and Cl^- ions in the rGO nanochannels (Supplementary Fig. 30). By comparison, a lower number density (0.0001-0.0002 atoms nm^{-3}) of Cl^- ions and a much higher number density (0.0010-0.0020 atoms nm^{-3}) of Na^+ ions are presented in the ArGO-PSSNa nanochannels. This suggests that the ArGO-PSSNa nanochannels can lead to the enrichment of Na^+ and the dissipation of Cl^-, strengthening the ion partitioning.

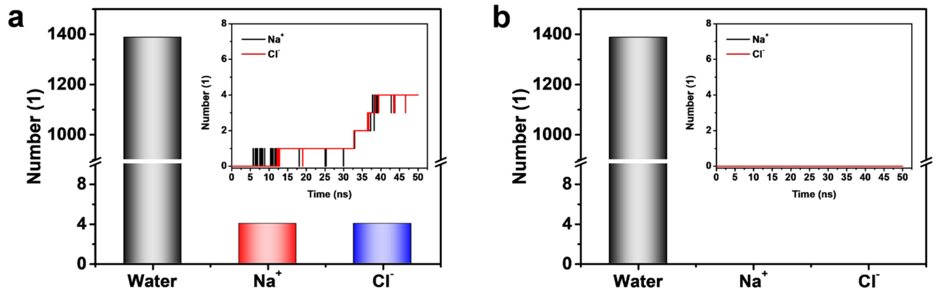

Supplementary Fig. 28. Simulated ion and water molecule numbers through the rGO and ArGO-PSSNa nanochannels. a, Number of water molecules, Na^+ and Cl^- transported through the rGO nanochannels. **b**, Number of water molecules, Na^+ and Cl^- transported through the ArGO-PSSNa nanochannels. The insets show the time variations of the number of Na^+ and Cl^- transported through the (**a**) rGO and (**b**) ArGO-PSSNa nanochannels.

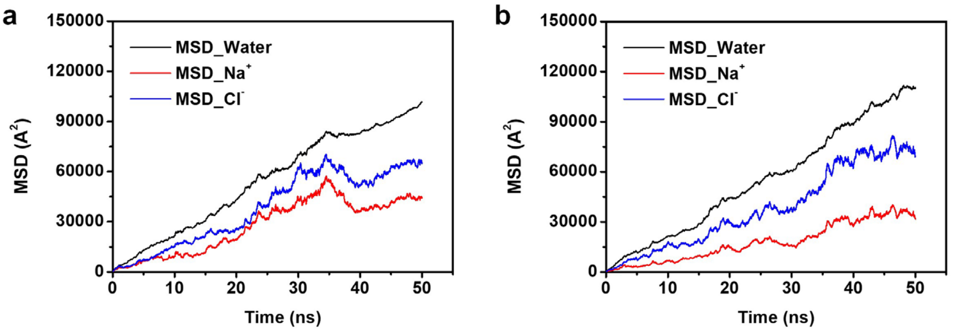

Supplementary Fig. 29. Simulated mean square displacement (MSD). MSD of water molecules, Na^+ and Cl^- through (**a**) rGO and (**b**) ArGO-PSSNa nanochannels as a function of time.

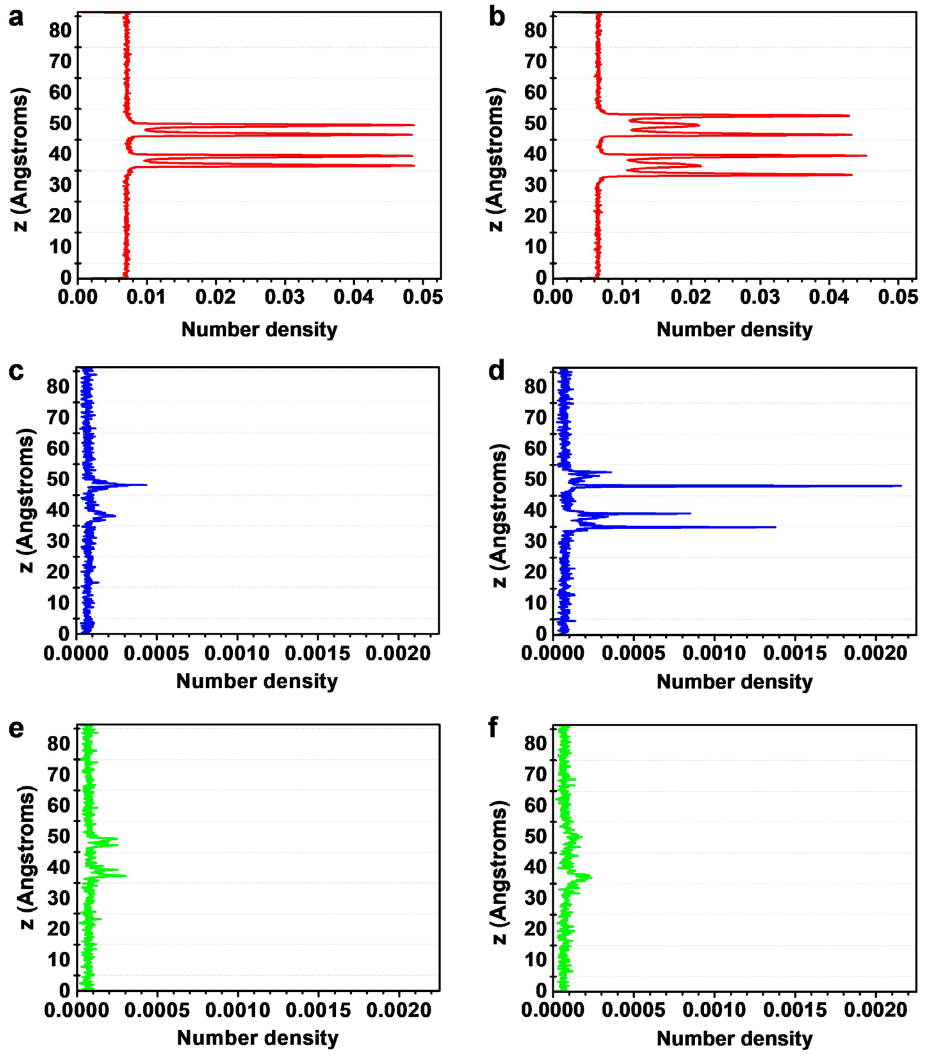

Supplementary Fig. 30 Number density distributions of water molecules, Na^+ and Cl^- ions along the direction (*z*-axis) perpendicular to rGO and ArGO-PSSNa nanochannels. a, Number density distributions of water molecules for rGO nanochannels. **b**, Number density distributions of water molecules for ArGO-PSSNa nanochannels. **c**, Number density distributions of Na^+ ions for rGO nanochannels. **d**, Number density distributions of Na^+ ions for ArGO-PSSNa nanochannels. **e**, Number density distributions of Cl^- ions for rGO nanochannels. **f**, Number density distributions of Cl^- ions for ArGO-PSSNa nanochannels.

Supplementary Note 11.

The LAMMPS MD simulation has been successfully used in describing the water and ion transport processes of many graphene-based membranes[40,41,44-46]. A scheme of "keeping LJ parameters and changing atomic charges" proposed by Vácha et al.[47] was adopted to check the forcefield[48] and further investigate ion transport behavior. The mulliken atomic charges were recalculated through MS software for LAMMPS MD simulations (Supplementary Table 5). Supplementary Fig. 31 shows the re-simulated results of ion permeation through rGO and ArGO-PSSNa nanochannels. The number of Na^+ and Cl^- in ArGO nanochannels is equivalent, demonstrating no ion partitioning. Whereas, the number of Na^+ is much higher than the number of Cl^- in ArGO-PSSNa nanochannels, suggesting that the ArGO-PSSNa nanochannels exhibit significant intra-nanochannel anion/cation partitioning. Moreover, the radial distribution functions (RDF) of Na^+ ions indicate that the Na^+ ions in the ArGO-PSSNa nanochannels undergo ion dehydration. These results are consistent with the simulation results with unmodified atomic charges.

Supplementary Table 5. The original and modified atomic charges used in the MD simulation[40,43].

Atom	q (e)	Modified q (e)
C (C−C)	0.000	0.000
C (C−O)	0.150	0.188
O (−OH)	−0.585	−0.596
H (−OH)	0.435	0.408
C (C−H)	−0.299	−0.290
H (C−H)	0.296	0.289
S (S−O)	2.210	2.270
O (S−O)	−0.960	−0.990
O (H_2O)	−0.848	−0.858
H (H_2O)	0.424	0.429
Na^+	1.000	1.000
Cl^-	−1.000	−1.000

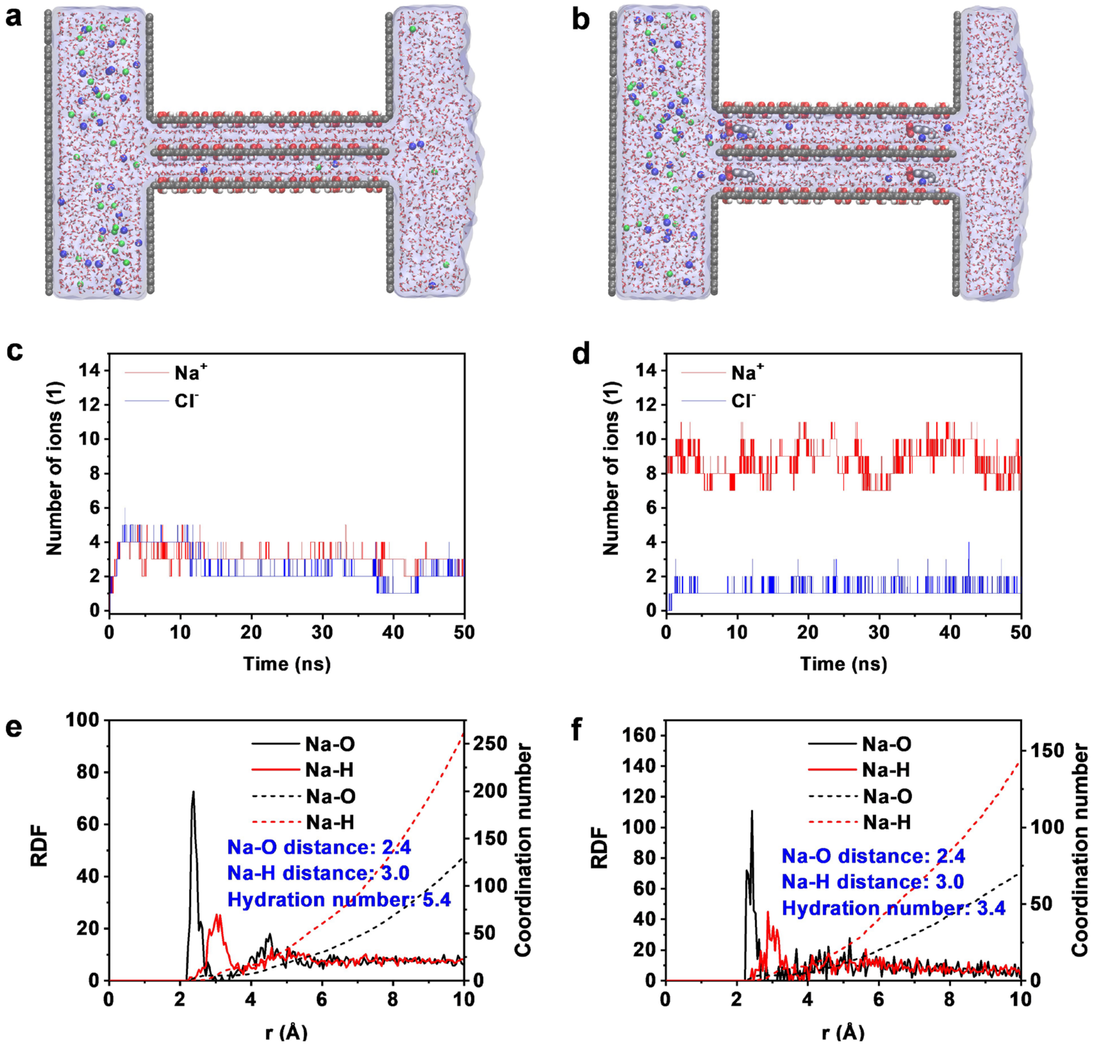

Supplementary Fig. 31. Re-simulated ion permeation through rGO and ArGO-PSSNa nanochannels using mulliken atomic charges recalculated through MS software. a, Snapshots of the MD simulations for NaCl solution transporting through the rGO nanochannels using the modified atomic charges. **b**, Snapshots of the MD simulations for NaCl solution transporting through the ArGO-PSSNa nanochannels using the modified atomic charges. **c**, Time evolution of the number of Na^+ and Cl^- in the rGO nanochannels. **d**, Time evolution of the number of Na^+ and Cl^- in the ArGO-PSSNa nanochannels. **e**, MD-calculated RDF and coordination numbers for oxygen and hydrogen of water molecules around the Na^+ ions in the feed side. **f**, MD-calculated RDF and coordination numbers for oxygen and hydrogen of water molecules around the Na^+ ions in the ArGO-PSSNa nanochannels. The solid line represents the RDF, and the dashed line denotes the coordination number.

Time variations of permeated water and salt

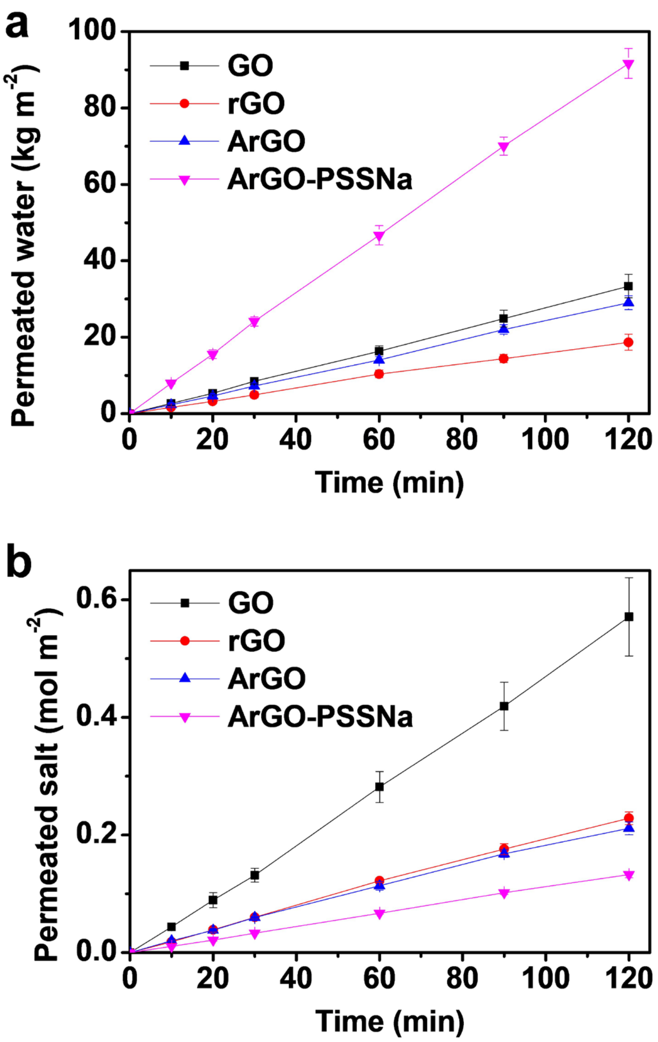

Supplementary Fig. 32. Water and salt permeation through membranes. a, Time variations of permeated water through GO, rGO, ArGO and ArGO-PSSNa membranes. **b**, Time variations of permeated salt through GO, rGO, ArGO and ArGO-PSSNa membranes. The lines between symbols are guides to the eye. The tests were conducted at room temperature under FO (the active layer facing the feed solution) mode using 0.5 M NaCl and DI water as the draw and feed solutions, respectively. Error bars represent the standard deviation of three replicate measurements.

Osmosis-driven membrane desalination stability

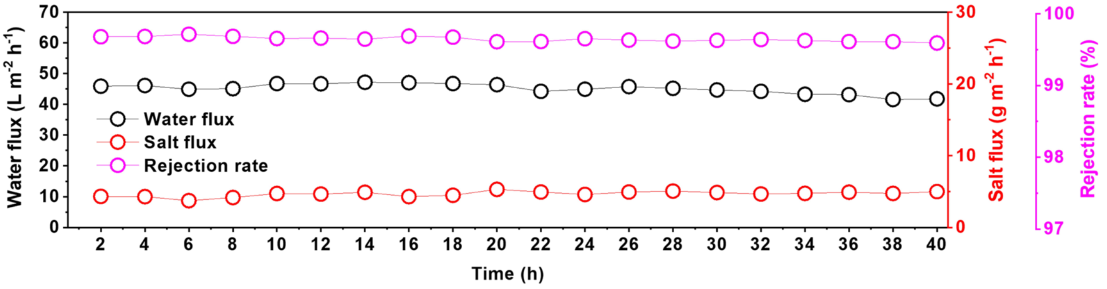

Supplementary Fig. 33. Osmosis-driven desalination stability of ArGO-PSSNa membrane. Water flux, salt flux and salt rejection rate of osmosis-driven ArGO-PSSNa membrane using 0.5 M NaCl as draw solution and DI water as feed solution during operation of 40 h. The lines between symbols are guides to the eye.

Osmosis-driven desalination performance for different salt concentrations

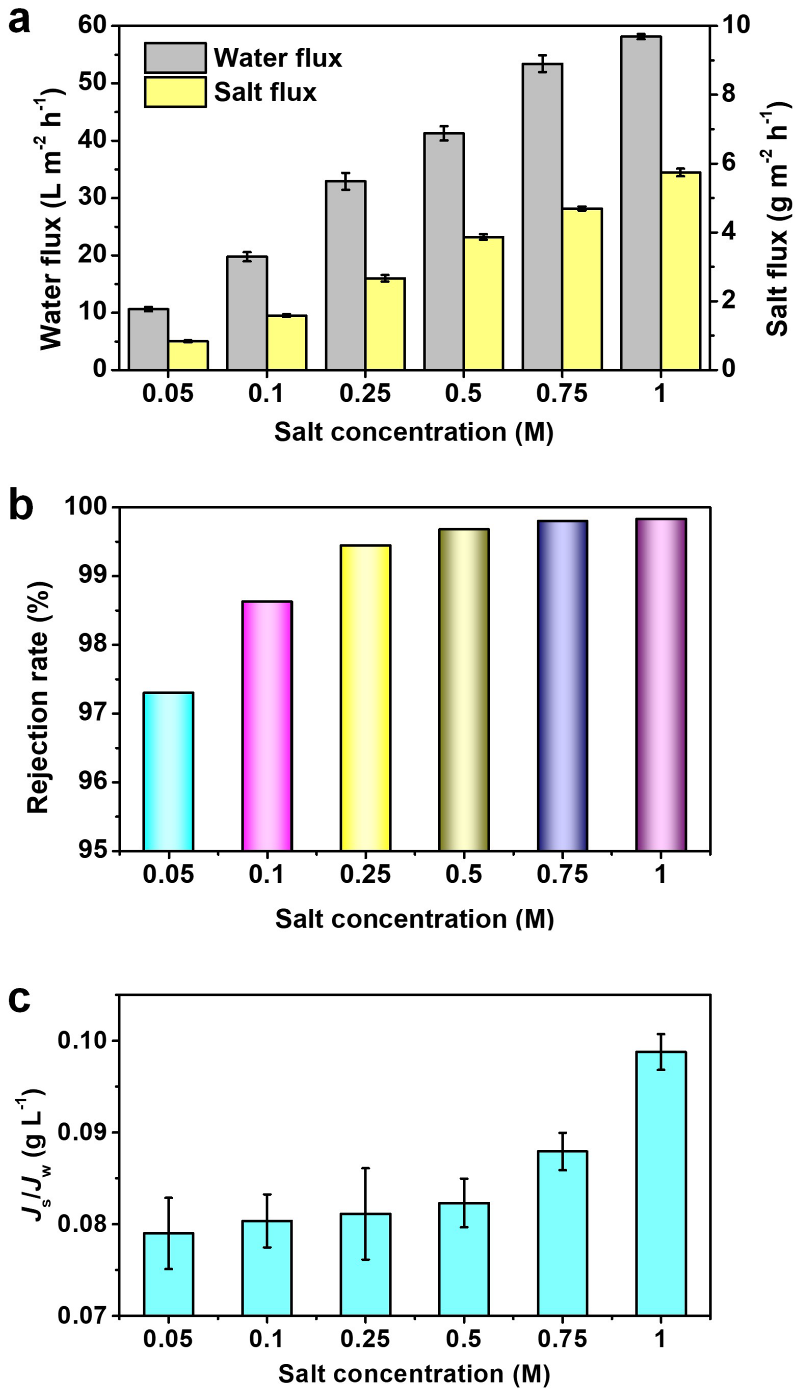

Supplementary Fig. 34. Osmosis-driven desalination performance of ArGO-PSSNa membrane for different salt concentrations. (**a**) Water flux and NaCl permeation flux, (**b**) salt rejection rate and (**c**) salt-water ratio (J_s/J_w) of ArGO-PSSNa membrane for different salt concentrations of 0.05, 0.1, 0.25, 0.5, 0.75 and 1 M. Error bars represent the standard deviation of three replicate measurements.

Supplementary Table 6. The performances of osmosis-driven ArGO-PSSNa membrane and some reported FO membranes.

Membranes	Feed/draw	J_v (LMH)	J_s (gMH)	J_s/J_v (g/L)	Ref.
pDA-rGO membrane	DI water/ 0.6 M NaCl	20.1	2.34	0.116	49
rGO	DI water/ 0.5 M NaCl	18	1.1	0.061	50
RGO/CNT	DI water/ 0.5 M NaCl	22.5	2.3	0.07	51
GO-OCNTs-LbL membrane	DI water/ 1.0 M NaCl	5.9	0.5	0.085	52
MWCNT/GO-PA Membrane	DI water/ 0.5 M NaCl	9.14	2.34	0.256	53
GO-incorporated TFC membranes	DI water/ 2.0 M NaCl	35.4	12	0.339	54
TFN-MMGO/Fe_3O_4-200 membrane	10 mM NaCl/ 1.0 M NaCl	45	4	0.0888	55
GO-FO membranes	DI water/ 0.5 M NaCl	6	1.02	0.17	56
PVP-GO	DI water/ 0.5 M NaCl	6.66	0.53	0.08	56
M-T/CNT-PA membranes	DI water/ 0.5 M NaCl	16	2.5	0.1625	57
TFC-GQD/MnO_2-2	DI water/ 0.5 M NaCl	17.89	4.259	0.238	58
PES-PVA-PA (500)	DI water/ 1.0 M NaCl	17.20	3.69	0.21	59
PA-GO/PAN	DI water/ 1.0 M NaCl	23	7.0	0.30	54
GO-embedded TFN 0.1	DI water/ 1.0 M NaCl	14.5	2.6	0.17	60
GO-modified PA TFN	DI water/ 0.5 M NaCl	24.72	5.2	0.21	61
PSf-PDA/GO-0.5g/L	DI water/ 1.0 M NaCl	24.3	3.818	0.157	62
MGO-8 TFN	DI water/ 0.5 M NaCl	24.72	5.2	0.21	61
TFC–PDA/GO-0.5	DI water/ 1.0 M NaCl	24.3	3.9	0.16	62

MXene interlayered FO membranes	DI water/ 0.5 M NaCl	15.6	1.1	0.07	63
Charcoal-CNM-TFC membrane	DI water/ 1.0 M NaCl	12.08	2.97	0.24	64
TFC-HClZNR membranes	DI water/ 1.0 M NaCl	28	8	0.285	65
SKL-modified TFCs	DI water/ 2.0 M NaCl	14	2.8	0.2	66
M/C-TFNi membrane	DI water/ 1.0 M NaCl	30	4.3	0.143	67
PVC/MOF@PDA(0.25) TFN-2	DI water/ 1.0 M NaCl	29.73	4.72	0.16	68
PES/UiO66-(F)4	DI water/ 1.0 M NaCl	38.7	11.6	0.30	69
PSf/UiO66	DI water/ 1.0 M NaCl	20.7	4.3	0.21	70
TFC-PEG-diamine-0.2 membrane	DI water/ 1.0 M NaCl	23.3	1.6	0.07	71
CTM-8/PA TFN	DI water/ 1.0 M NaCl	24	8	0.333	72
PA-PDA/PEI/UiO-66(0.1) membranes	DI water/ 1.0 M NaCl	40	3.28	0.082	73
PPBES-TFC	DI water/ 0.5 M NaCl	45.3	6.8	0.15	74
ACN membranes	0.1 M NaCl/ 2 M sucrose	3	3.15	1.17	75
400-layer stacked MoS_2 membrane	DI water/ 1.0 M NaCl	5.4	0.585	0.108	76
ArGO-PSSNa membrane	DI water/ 0.5 M NaCl	47.0	3.8	0.08	This work
	DI water/ 1 M NaCl	58.1	5.7	0.098	

Supplementary Note 12.

<u>Transmembrane energy barriers for salt permeation and individual ion transports</u>

The transmembrane energy barriers of salt permeation through the GO, rGO, ArGO and ArGO-PSSNa membranes in osmosis-driven process were determined by the concentration gradient-driven salt permeation with 0.5 M NaCl solution (feed side) and deionized water (permeate side). Based on the salt permeation flux−temperature relationship, the transmembrane energy barrier of salt permeation was determined according to the Arrhenius-type equation.

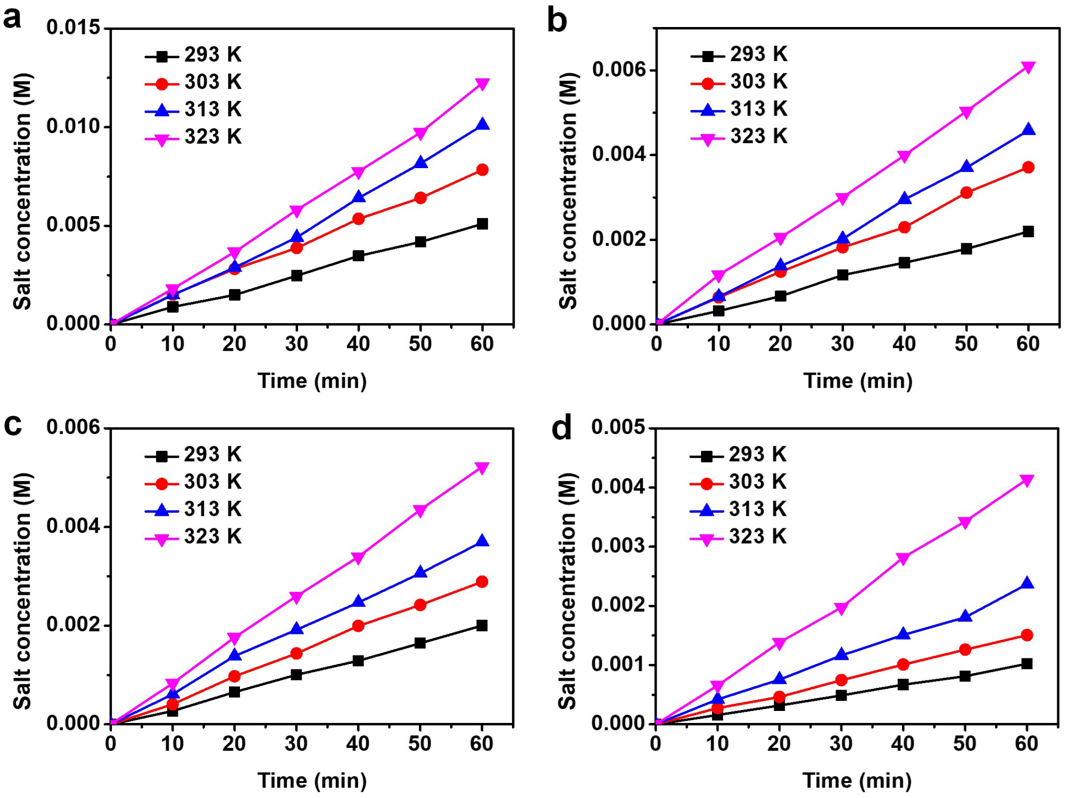

Supplementary Fig. 35. Salt ion permeation through membranes at different temperatures. Time variations of salt concentrations permeated through (**a**) GO, (**b**) rGO, (**c**) ArGO and (**d**) ArGO-PSSNa membranes at different temperatures of 293, 303, 313 and 323 K. The lines between symbols are guides to the eye. The salt permeation tests were conducted in a two-compartment cell filled with a 0.5 M NaCl solution and deionized water, and the permeated salt concentration was measured via electrical conductivity.

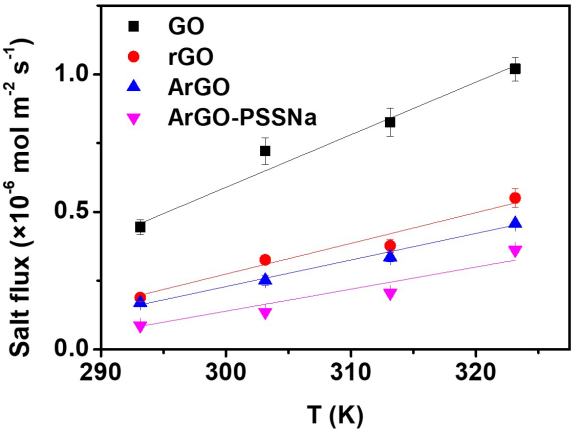

Supplementary Fig. 36. Variation of salt flux of membranes over temperature. Salt fluxes of GO, rGO, ArGO and ArGO-PSSNa membranes for 0.5 M NaCl solution as a function of the temperature. The lines denote the linear fits made with the numerical model.

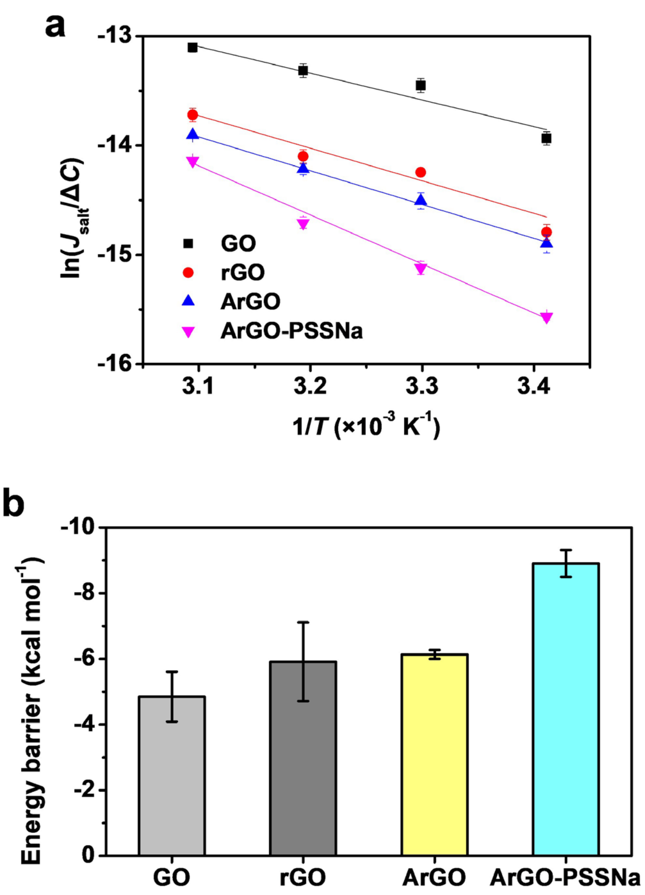

Supplementary Fig. 37. Salt ion transmembrane energy barriers of membranes. a, Arrhenius plots for salt (0.5 M NaCl solution) transport through GO, rGO, ArGO and ArGO-PSSNa membranes. Natural logarithm of the salt flux divided by salt concentration difference of two cells ($J_{salt}/\Delta C$) is plotted as a function of $1/T$. The lines denote the linear fits made with the numerical model. **b**, Energy barriers for salt (5 mM NaCl solution) during the transmembrane process through GO, rGO, ArGO and ArGO-PSSNa membranes. Error bars represent the standard deviation of three replicate measurements.

To study the transmembrane energy barriers of individual cations and anions, an electrochemical method was used to decouple the transports of anions and cations[36,37]. The electrochemical tests were performed in the home-made two-compartment diffusion cell. The membrane was fixed between the two compartments. By applying an external voltage cross the membrane, the electric field force drove the transports of anions and cations in opposite directions, which could distinguish the decoupled transports of individual anions and cations, obtaining the transmembrane energy barriers of individual ions. The energy barrier can be calculated by the linearized form of an Arrhenius-type equation:

$$\ln(Gt_{\pm}T) = \ln(B_{\pm}) - \frac{E_{\pm}}{R} \cdot \frac{1}{T} \tag{7}$$

where G is the ion conductance through the membrane, $t_{\pm}$ is the transport number for cations or anions, which satisfies $t_{+}+t_{-}=1$, $B_{\pm}$ is the pre-exponential factor for cations or anions, and $E_{\pm}$ is the transmembrane energy barrier for individual cations or anions.

In order to calculate the transmembrane energy barrier $E_{\pm}$, it is necessary to first determine the G and $t_{\pm}$, which are temperature-dependent parameters. The G can be obtained by testing the I−V curves at different temperatures. In the two-compartment cell, 0.5 M NaCl solution was put into the two chambers, and two Ag/AgCl electrodes served as the working electrode and counter electrode. An electrochemical workstation was used to test the I−V curves at various temperatures of 293, 303, 313 and 323 K. Based on the slope of the I−V curve, the temperature-dependent ion conductance through the membrane can be obtained.

The transport number $t_{\pm}$ can be calculated by the following formula:

$$\Delta \Phi_{\mathrm{m}} = (2t_{+} - 1)\frac{RT}{F}\ln\left(\frac{C_{\mathrm{h}}\alpha_{\mathrm{h}}}{C_{\mathrm{l}}\alpha_{\mathrm{l}}}\right) \tag{8}$$

where $\Delta\Phi_{\mathrm{m}}$ is the membrane potential, t_{+} is the transport number for cations, F is the Faraday constant (96485 C mol^{-1}), C_{h} and C_{l} are the high and low salt concentrations in the two chambers, respectively, and α_{h} and α_{l} are the activity coefficients of the salt with high and low concentrations. Accordingly, t_{+} can be obtained from the slope of the

Formula (8) when the membrane potential $\Delta\Phi_m$ is measured. Thus, it is necessary to determine the membrane potential $\Delta\Phi_m$. Specifically, a NaCl solution with a high concentration (C_h) of 0.5 M was placed in one chamber of the two-compartment cell, while another NaCl solution with a low concentration (C_l) of 0.05, 0.10, 0.25 or 0.5 M was added in the other chamber, forming the C_h/C_l ratio of 10, 5, 2 or 1, respectively. An electrochemical workstation and two Ag/AgCl electrodes were used to test the linear sweep voltammetry (LSV) curves at various temperatures of 293, 303, 313 and 323 K. The membrane potential ($\Delta\Phi_m$) was determined as the intersection between the LSV curve and x-axis at each C_h/C_l ratio.

With the calculated G and t_+ at different temperatures, the transmembrane energy barrier E_+ of cations (Na^+) was calculated by the linearized form of an Arrhenius-type equation (7). Besides, the transmembrane energy barrier E_- of anions (Cl^-) was calculated using the parameter t_-, where $t_-=1-t_+$.

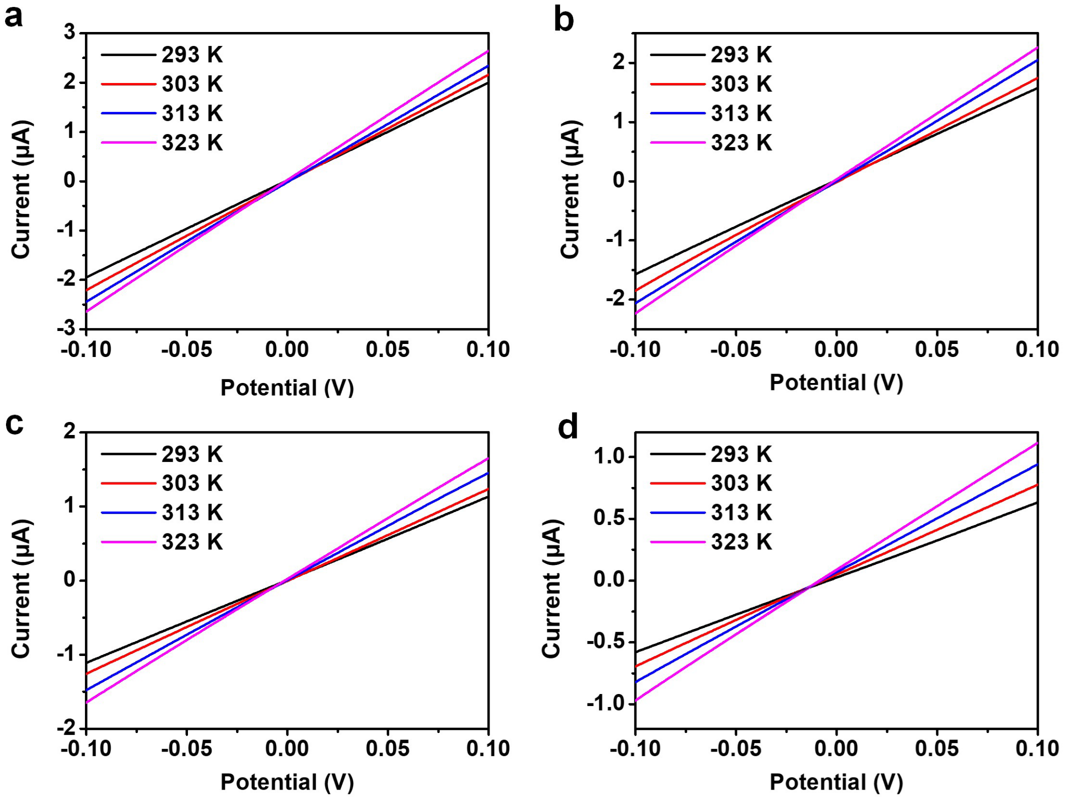

Supplementary Fig. 38. Electrochemical *I*−*V* curves of membranes. *I*−*V* curves of (**a**) GO, (**b**) rGO, (**c**) ArGO and (**d**) ArGO-PSSNa membranes under 0.5 M NaCl at different temperatures of 293, 303, 313 and 323 K.

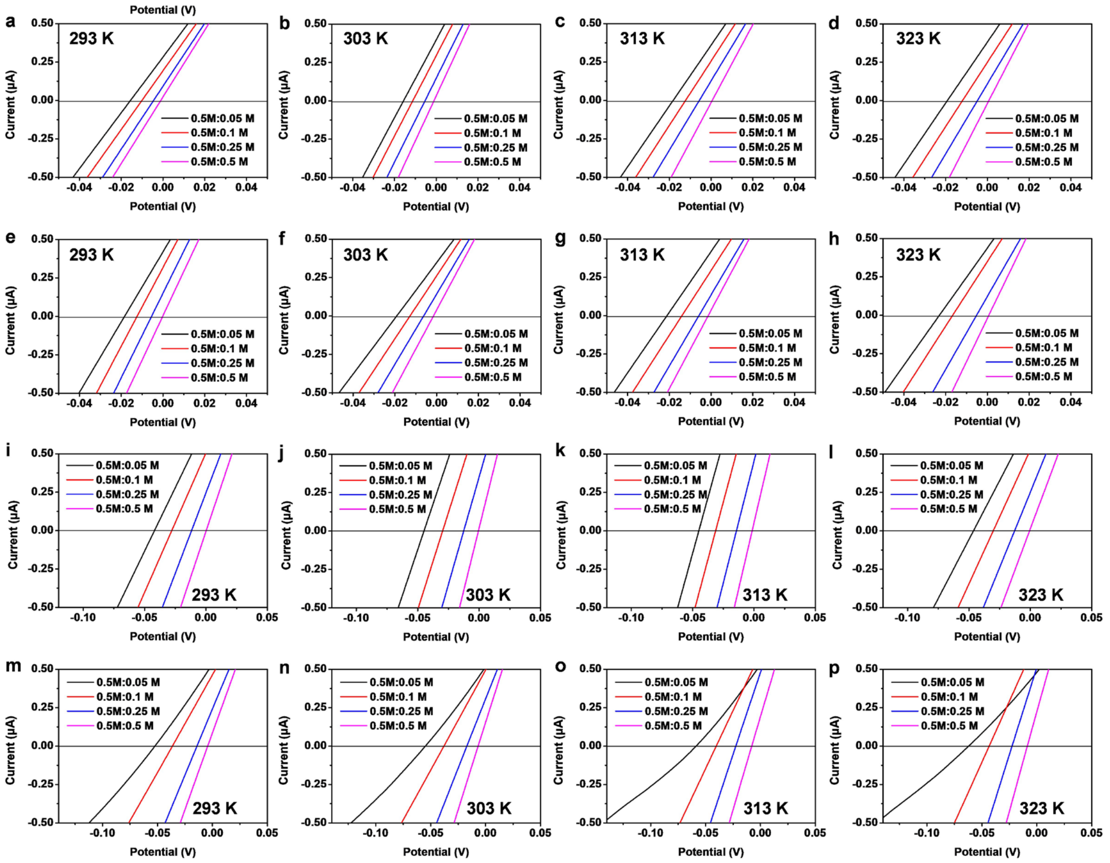

Supplementary Fig. 39. Electrochemical LSV curves of membranes for membrane potential measurements. Membrane potentials across (**a-d**) GO, (**e-h**) rGO, (**i-l**) ArGO and (**m-p**) ArGO-PSSNa membranes at different temperatures of 293, 303, 313 and 323 K using electrochemical LSV measurements at a scan rate of 2 mV s^{-1}. The intersection between the LSV curve and *x*-axis is expressed as the membrane potential. The tests were conducted in a two-compartment cell. one chamber contains 0.5 M NaCl and the other chamber was filled with a NaCl solution with the concentration varying from 0.05, 0.1, 0.25 and 0.5 M, obtaining the concentration ratios of 10:1, 5: 1, 2:1 and 1:1, respectively, for two chambers.

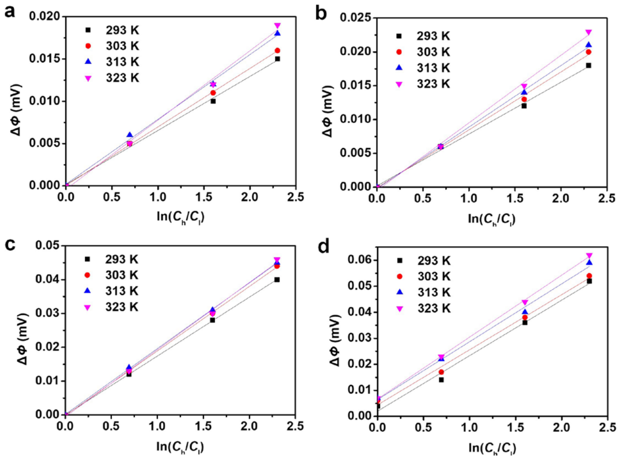

Supplementary Fig. 40. Membrane potential as functions as the concentration ratio. Membrane potentials across (**a**) GO, (**b**) rGO, (**c**) ArGO and (**d**) ArGO-PSSNa membranes at different temperatures of 293, 303, 313 and 323 K as functions as the concentration ratio between the two chambers. The lines denote the linear fits made with the numerical model.

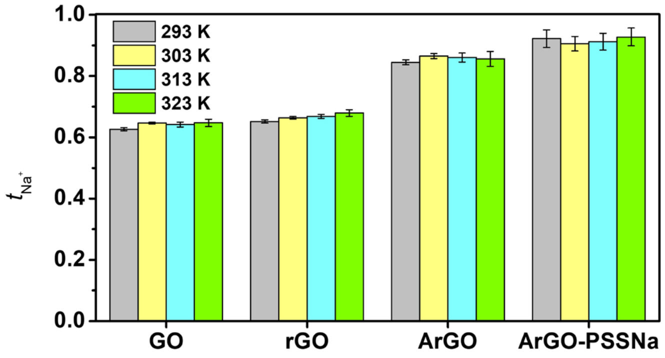

Supplementary Fig. 41. Na$^+$ transport number. Transport number of Na$^+$ (t_{Na^+}) with GO, rGO, ArGO and ArGO-PSSNa membranes at different temperatures of 293, 303, 313 and 323 K. Error bars represent the standard deviation of three replicate measurements.

Supplementary Note 13.

Molecular dynamics (MD) simulations of osmosis-driven desalination

MD simulations were conducted to investigate the osmosis-driven desalination processes of rGO and ArGO-PSSNa membranes. We constructed a simulated two-compartment system configuration with the size of 202×19×86 $Å^3$, containing a region of salt solution (left) and a region of DI water (right) (Supplementary Fig. 42). The salt solution region included 3186 water molecules, 58 Na^+ ions and 58 Cl^- ions, and the DI water region possesses 3374 water molecules. Two graphene nanosheets (pistons) were placed at the left and right ends of the system. The salt solution region and DI water region were separated by two nanochannels. The rGO nanochannels were modeled using three rGO nanosheets with the area of 35×19 $Å^2$ and the interlayer width of 10 Å (Supplementary Fig. 42a). The ArGO-PSSNa nanochannels were constituted by imbedding a monomer molecule of sodium polystyrenesulfonate (PSSNa) into each rGO nanochannel with the interlayer width of 13 Å (Supplementary Fig. 42b). Generally, the mass transfer of osmosis-driven process is slower than that of pressure-driven process, thus we use a shorter nanochannel length of 35 Å for rGO and ArGO-PSSNa nanochannels to inspecting the osmosis-driven desalination over a relatively short time period.

The MD simulations were performed using the LAMMPS package[77-79], which is similar to the simulation of pressure-driven desalination process in this work. During the MD simulations, two horizontal forces with opposite directions were imposed on two graphene nanosheets (pistons) for providing an atmosphere pressure (0.1 MPa) to compress the salt solution and DI water. All the simulations were conducted in the NVT ensemble at 300 K. The energy minimization was first carried out, and then a 100 ps equilibration was run with the time step of 1 fs, and a 50 ns simulation was performed for data collection. The postprocessing was carried out using Visual Molecular Dynamics (VMD)[40].

Supplementary Fig. 43 shows the number of water molecules, Na^+ and Cl^- ions in the salt solution region (feed side (F)) and DI water region (permeate side (P)). Water

molecules transported from the DI water side to the salt solution side, while Na^+ and Cl^- ions transferred from the salt solution side to the DI water side. There is 2 Na^+ and 2 Cl^- ions passing through the rGO nanochannels in anion–cation pairs-like formation due to the electroneutrality principle, but no ions traverses through the ArGO-PSSNa nanochannels. By comparing the number of Na^+ and Cl^- ions in rGO and ArGO-PSSNa nanochannels, it is found that there is one Na^+ and one Cl^- in the rGO nanochannels throughout the simulation period (Supplementary Fig. 44a), implying no ion partitioning. Whereas, in the ArGO-PSSNa nanochannels, the number of Na^+ (5 Na^+) is significantly greater than the number of Cl^- (1 Cl^-) (Supplementary Fig. 44b), demonstrating the occurrence of ion partitioning. This is consistent with the simulation results of pressure-driven desalination process, confirming that the ArGO-PSSNa membrane nanochannels can strengthen the ion partitioning to break the dynamical correlations between the free (mobile) anion–cation pairs and thereby improve the salt rejection.

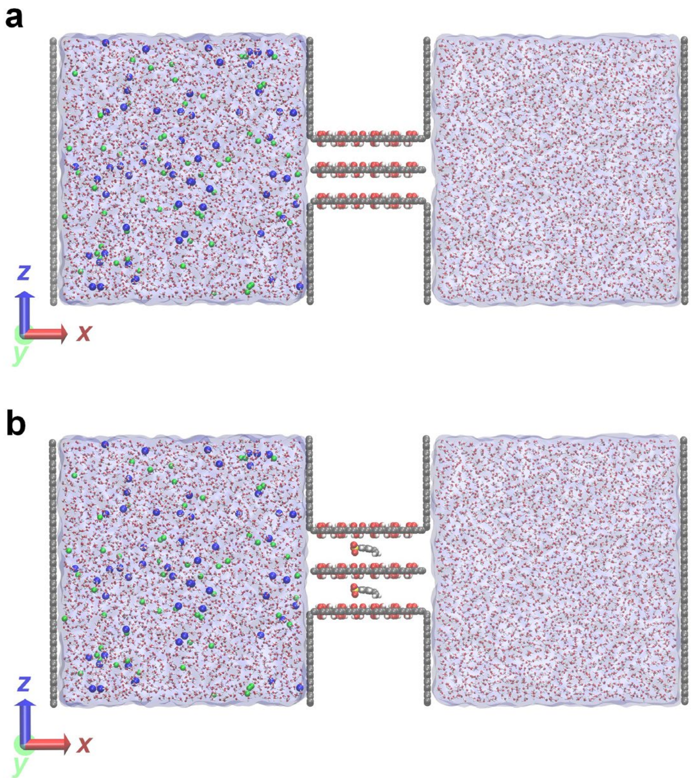

Supplementary Fig. 42. Configurations of rGO and ArGO-PSSNa nanochannels for MD simulation. **a**, Simulation configuration for water desalination through the rGO nanochannels. **b**, Simulation configuration for water desalination through the ArGO-PSSNa nanochannels. The simulation box is 202×19×86 Å^3. The length of the rGO and ArGO-PSSNa nanochannels is 35 Å,

and the widths are 10 Å for rGO nanochannels and 13 Å for ArGO-PSSNa nanochannels. There were 3186 water molecules, 58 Na^+ ions and 58 Cl^- ions in the simulated salt solution (feed side), and 3374 water molecules are in the simulated DI water (permeate side). The C, O, H and S atoms are shown in grey, red, white and yellow spheres, separately. Blue and green spheres represent Na^+ and Cl^- ions.

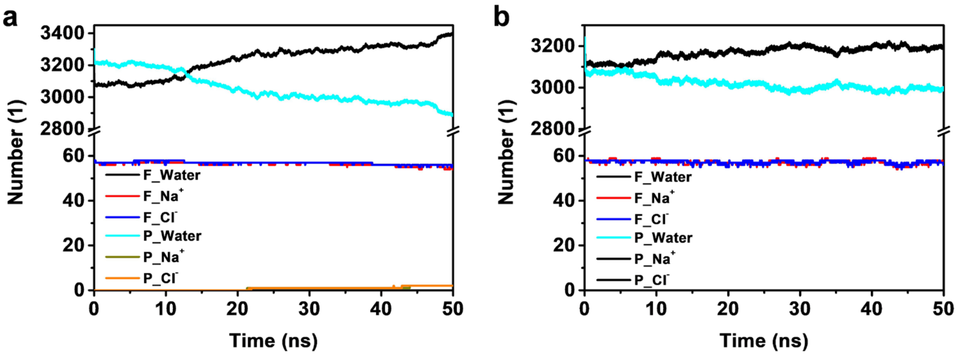

Supplementary Fig. 43. Simulated water molecule and ion numbers through the rGO and ArGO-PSSNa nanochannels. Time variations of the number of water molecules, Na^+ and Cl^- ions in the feed side (F) and permeate side (P) for the simulations of (**a**) rGO and (**b**) ArGO-PSSNa nanochannels.

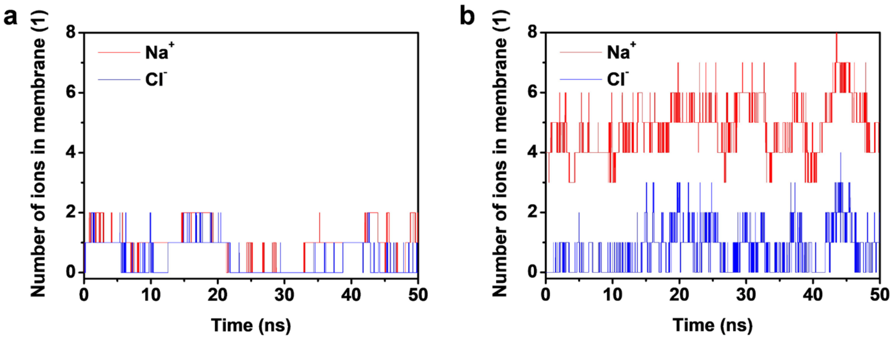

Supplementary Fig. 44. Simulated ion numbers in rGO and ArGO-PSSNa nanochannels. Time evolutions of the number of Na^+ and Cl^- ions in the (**a**) rGO and (**b**) ArGO-PSSNa nanochannels.

The radial distribution functions (RDF) and coordination numbers for oxygen and hydrogen of water molecules around the Na^+ ions were calculated from the MD simulation, as shown in Supplementary Fig. 45. The hydration number of Na^+ ions in the salt solution side is 5.5, indicating that the Na^+ ions are fully hydrated (Supplementary Fig. 45a,c). After entering the nanochannels, the Na^+ ions are dehydrated because of the nanoconfinement of the nanochannels. Notably, the Na^+ ions in the rGO nanochannels show an average hydration number of 2.4 (Supplementary Fig. 45b), whereas the average hydration number of Na^+ in the ArGO-PSSNa nanochannels is 4.4 (Supplementary Fig. 45d). Although the rGO nanochannels possess stronger ion-

dehydration ability, they exhibit a relatively low salt rejection performance, compared with the rGO-PSSNa nanochannels. This suggests that sole effect of nanoconfined ion dehydration cannot result in improved salt rejection. The specific explanation is that, the rGO nanochannels induce a strong dehydration effect of Na^+ ions, but the electrostatic attraction interactions between the rGO nanochannels and Na^+ ions are too weak to confine the dehydrated Na^+ ions in the nanochannels (Supplementary Fig. 46a). As a result, the Na^+ and accompanying Cl^- are co-transported through the nanochannels in the form of free mobile cation/anion pairs (governed by the electrical neutrality principle). In contrast, the ArGO-PSSNa nanochannels have strong electrostatic attraction interactions with the Na^+ ions, inducing the dehydration of Na^+ ions and forcing the dehydrated Na^+ ions to be strongly confined in the nanochannels, which leads to strong intra-nanochannel anion/cation partitioning (Supplementary Fig. 46b). This can break the dynamically correlated anion–cation pairs, suppressing the transmembrane co-transport of free mobile anions and cations, resulting in high salt rejection.

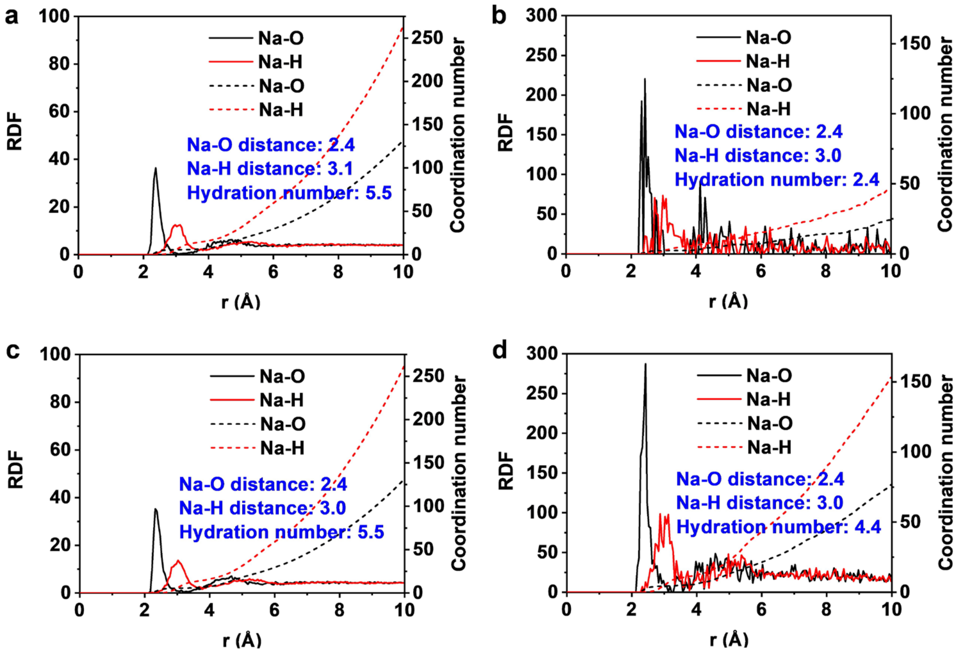

Supplementary Fig. 45. Simulated radial distribution functions (RDF) and coordination numbers. a,**b**, RDF and coordination numbers for oxygen and hydrogen in water molecules around the Na^+ ions in the (a) salt solution side and (b) rGO nanochannels. **c**,**d**, RDF and coordination numbers for oxygen and hydrogen in water molecules around the Na^+ ions in the (c) salt solution side and (d) ArGO-PSSNa nanochannels. The solid line represents the RDF, and the dashed line denotes the coordination number.

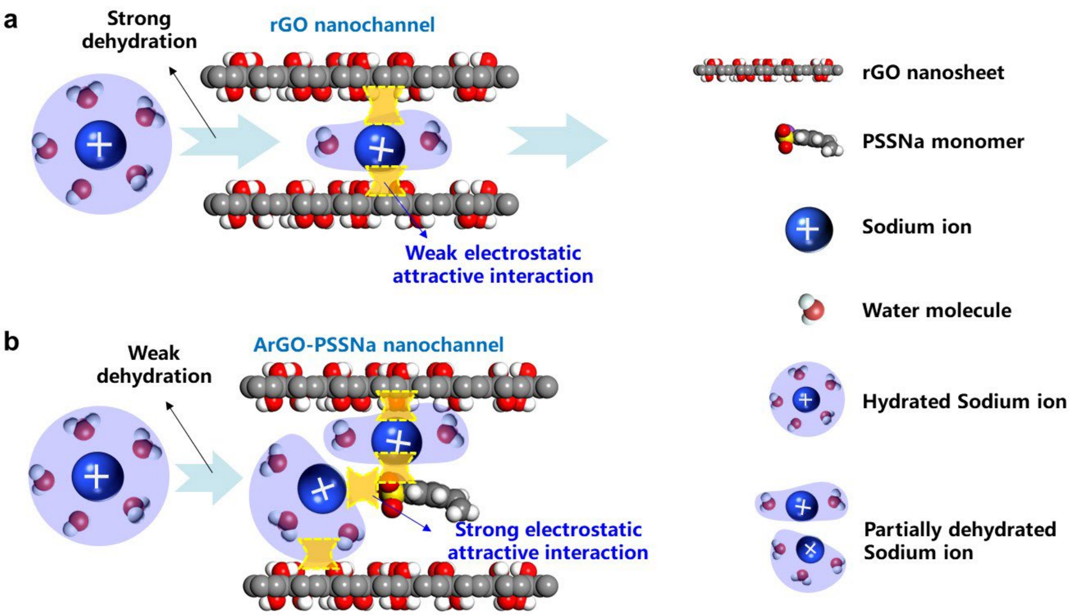

Supplementary Fig. 46. Schematics of ion dehydration effect and electrostatic attraction interactions. a, Strong dehydrated ions can transport through rGO nanochannel due to the lack of strong electrostatic attractive interaction between the nanochannel and ion. **b**, Weak dehydrated ions cannot transport through ArGO-PSSNa nanochannel due to the strong electrostatic attractive interaction between the nanochannel and ion.

Supplementary Note 14.

<u>DFT calculation of ion-membrane ineractions</u>

The electrostatic attraction interactions between Na^+, rGO and PSS were investigated by density functional theory (DFT) calculations utilizing the DMol3[80] module implemented in Materials Studio software for theoretically illuminating the membrane-Na^+ interaction mechanism. A monomer of sodium polystyrenesulfonate (PSSNa) and a rGO nanosheet (constituted by a graphene nanosheet with a hydroxyl group) whose edge was terminated with H atoms were constructed. Electronic exchange and correlation effects were described by the generalized gradient approximation (GGA)-Perdew-Burke-Ernzerhof (PBE) method. In addition, the core treatment was set as DFT semi-core pseudopots and the basis set was double-numerical plus polarization (DNP)[81]. The global cutoff of 4.9 Å was set to ensure high computational quality for both geometry optimization and electric structure calculations. Structure relaxation proceeded until the energy was < 2×10^{-5} Ha and the maximum force was < 0.004 Ha $Å^{-1}$. The adsorption energy (E_{ads}) was calculated with the following equations:

$$\Delta E_{ads,rGO\text{-}Na^+}=E_{total}-E_{rGO}-E_{Na^+} \quad (9)$$

$$\Delta E_{ads,rGO\text{-}PSS}=E_{total}-E_{rGO}-E_{PSS} \quad (10)$$

$$\Delta E_{ads,PSS\text{-}Na^+}=E_{total}-E_{PSS}-E_{Na^+} \quad (11)$$

$$\Delta E_{ads,rGO\text{-}PSS\text{-}Na^+}=E_{total}-E_{rGO\text{-}PSS}-E_{Na^+} \quad (12)$$

where E_{total}, E_{rGO}, E_{PSS}, $E_{rGO\text{-}PSS}$ and E_{Na^+} are the total energies of the calculation system, the rGO nanosheet (including the water molecules), rGO-PSS system (including the water molecules), and an isolated Na^+, respectively.

Supplementary Table 7. Calculated energies (Ha) for the systems of rGO-Na, rGO-PSS, PSS-Na and rGO-PSS-Na.

Systems	E_{total} (Ha)	$E_{\text{rGO or/and PSS}}$ (Ha)	$E_{\text{Na}^{+}\text{ or PSS}}$ (Ha)	ΔE_{ads} (Ha)
rGO-Na^+	−2153.4618487	−1991.182778	−162.160498	−0.1185727
rGO-PSS	−2923.5015569	−1991.182778	−932.2560018	−0.0627771
PSS-Na^+	−1094.5752866	−932.2560018	−162.160498	−0.1587868
rGO-PSS-Na^+	−3085.8211227	−2923.5015569	−162.160498	−0.1590678

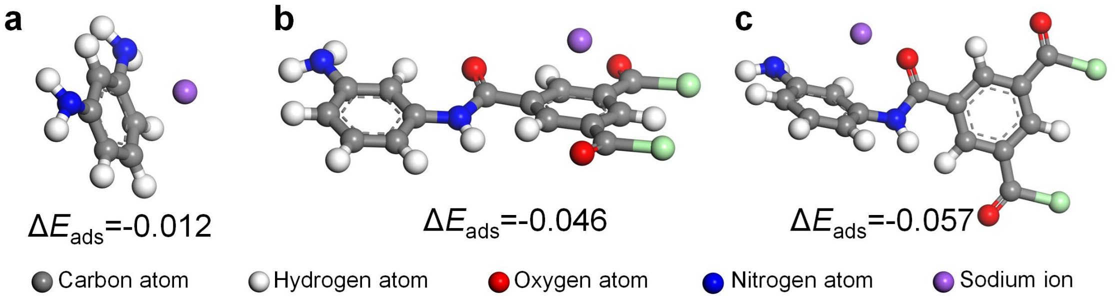

Supplementary Fig. 47. DFT-calculated adsorption configuration of Na^+ on phenylenediamine monomer and its polymer with trimesoyl chloride. a, Optimized adsorption sites and adsorption energies of Na^+ on phenylenediamine monomer. **b**,**c**, Optimized adsorption sites and adsorption energies of Na^+ on phenylenediamine polymer with trimesoyl chloride.

Supplementary Table 8. Calculated energies (Ha) for the systems of monomer-Na^+ and polymer-Na^+.

Systems	E_{total} (Ha)	$E_{rGO\text{-}PSS}$ (Ha)	E_{ion} (Ha)	ΔE_{ads} (Ha)
monomer-Na^+	−504.789	−342.617	−162.160	−0.012
polymer-Na^+	−1994.350	−1832.144	−162.160	−0.046
polymer-Na^+	−1994.361	−1832.144	−162.160	−0.057

Supplementary Note 15.

XPS etching spectra and element contents

In order to determine the electrostatic attractive interactions between rGO, PSS and Na^+, we fabricated three membranes of ArGO-Na, ArGO-PSS and ArGO-PSSNa for analyzing their XPS etching spectra. The fabrication processes of ArGO-Na and ArGO-PSS membranes were similar to that of ArGO-PSSNa membrane. Briefly, for preparing the ArGO-Na membrane, 4 mL rGO dispersion was mixed with 5 mL PSSNa (1.0 wt.% in 1 M NaCl solution) and 5 mL m-phenylenediamine (2.0 wt.%), which was then diluted to a 20 mL mixture with water. After ultrasonication for 10 min, the mixture was filtrated on a PVDF membrane substrate with vacuum assistance. After removing the excess solution, the resulting membrane was soaked in 0.1 w/v% trimesoyl chloride solution in n-hexane for 60 s. The excess solution on the membrane was drained off. Finally, the membrane was cured at 80 °C for 10 min in an oven and rinsed using deionized water to obtain the ArGO-Na membrane.

For fabricating the ArGO-PSS membrane, 4 mL rGO dispersion was mixed with 5 mL polystyrenesulfonate solution (1.0 wt.%) and 5 mL m-phenylenediamine (2.0 wt.%), which was then diluted to a 20 mL mixture with water. After ultrasonication for 10 min, the mixture was filtrated on a PVDF membrane substrate with vacuum assistance. After removing the excess solution, the resulting membrane was soaked in 0.1 w/v% trimesoyl chloride solution in n-hexane for 60 s. The excess solution on the membrane was drained off. Finally, the membrane was cured at 80 °C for 10 min in an oven and rinsed using deionized water to obtain the ArGO-PSS membrane.

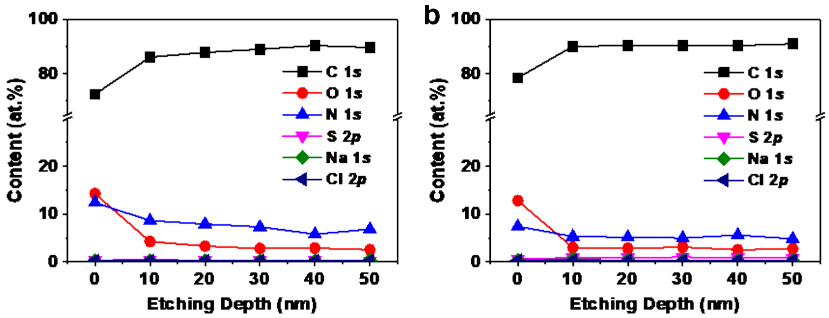

Supplementary Fig. 48. XPS etching spectra and element contents. a, Variation of element content over XPS etching depth in ArGO-Na membrane. **b**, Variation of element content over XPS etching depth in ArGO-PSS membrane. The lines between symbols are guides to the eye.

Supplementary Note 16.

Ion distributions in the nanochannels

Supplementary Fig. 49 shows the schematic diagrams of the ion distribution in rGO and ArGO-PSSNa nanochannels. For the rGO nanochannel, the relatively weak rGO-Na^+ attraction interaction force allows Na^+ and Cl^- to co-transport through the nanochannel (Supplementary Fig. 49a). Whereas, for the ArGO-PSSNa nanochannel, the strong rGO-PSS-Na^+ synergistic electrostatic attraction interaction force can confine more Na^+ in the nanochannel while the Cl^- is excluded from the nanochannel, making the free mobile anion-cation pairs broken (Supplementary Fig. 49b). In the nanochannel, the Na^+ ions are indeed much more than the Cl^- ions, meaning that the solution inside the nanochannel has excess positive charges. Whereas, the excess positive charges (Na^+) can be balanced by the negatively charged ArGO-PSSNa nanochannel, i.e., cation charges = anion charges + membrane charges, allowing the electrical neutrality to be maintained in the nanochannels.

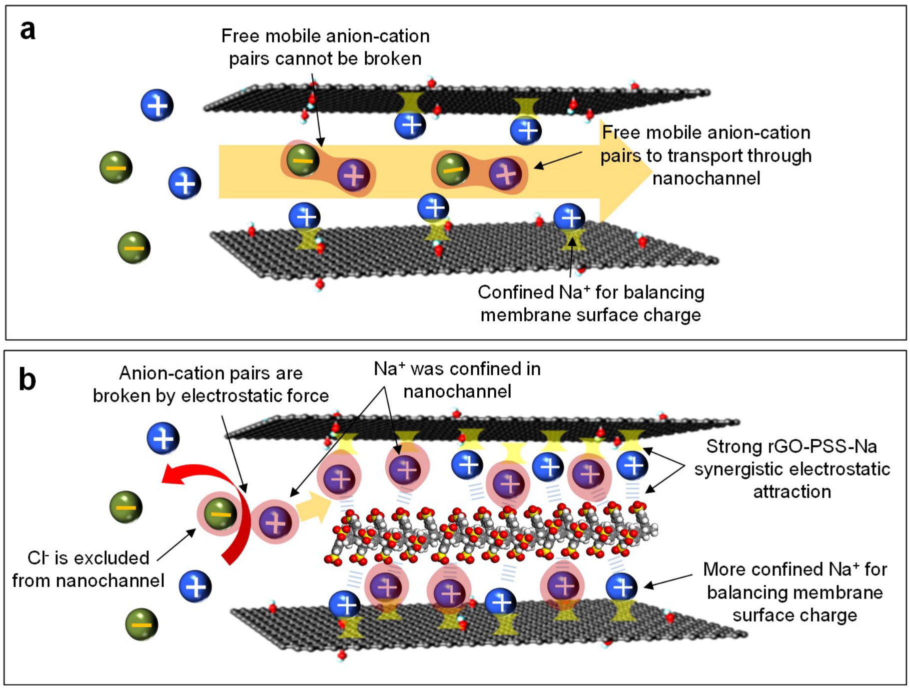

Supplementary Fig. 49. Ion distributions in rGO and ArGO-PSSNa nanochannels. a, Schematic diagram of the ion distribution in rGO nanochannel. **b**, Schematic diagram of the ion distribution in ArGO-PSSNa nanochannel.

Pressure-driven membrane module and setup

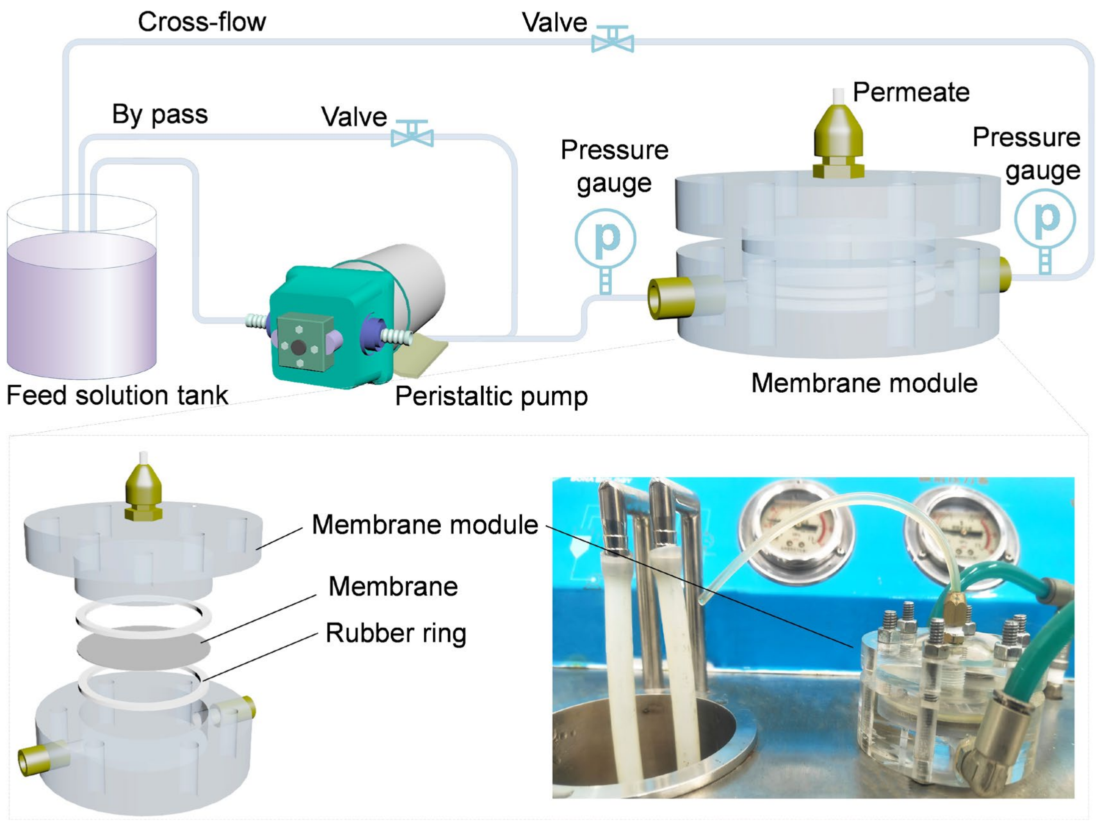

Supplementary Fig. 50. Membrane module and setup. Diagram of the pressure-driven cross-flow membrane module and filtration setup.

Time variation of NaCl rejection rate

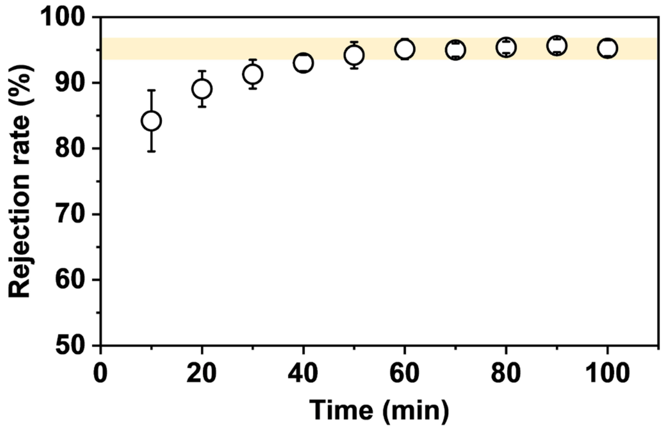

Supplementary Fig. 51. NaCl rejection rate as a function of filtration time. Time variation of the NaCl rejection rate of ArGO-PSSNa membrane for filtering 5 mM NaCl feed solution. Error bars represent the standard deviation of three replicate measurements, and the light orange region means that the rejection rate is constant at 95.3±1.5%.

Osmosis-driven membrane module and setup

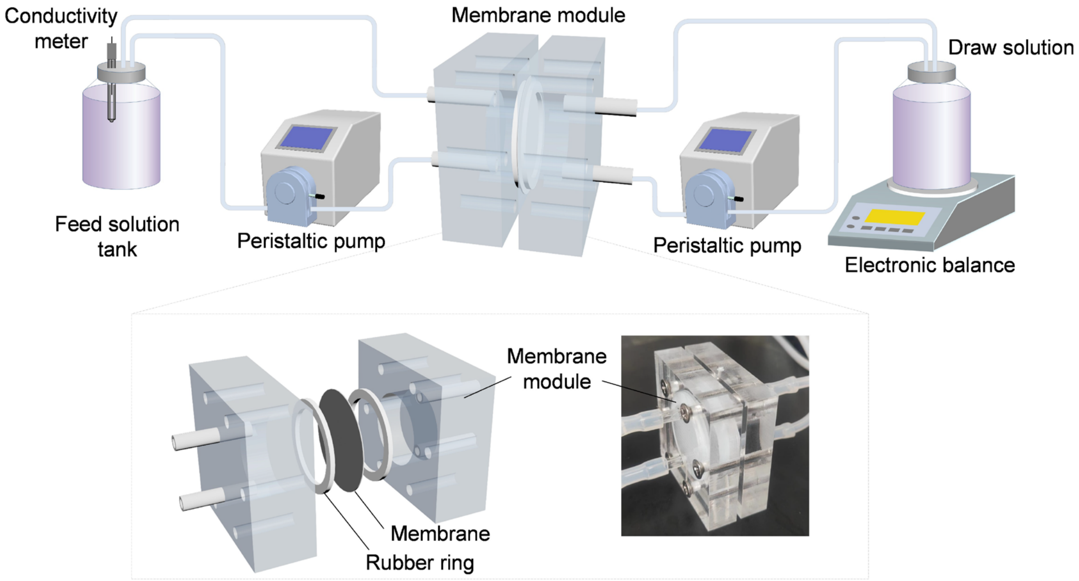

Supplementary Fig. 52. Membrane module and setup. Diagram of the osmosis-driven two-compartment membrane module and filtration setup.

Supplementary references

1 Zhao, H., Wu, L., Zhou, Z., Zhang, L. & Chen, H. Improving the antifouling property of polysulfone ultrafiltration membrane by incorporation of isocyanate-treated graphene oxide. *Phys. Chem. Chem. Phys.* **15**, 9084-9092 (2013).

2 Nair, R. R., Wu, H. A., Jayaram, P. N., Grigorieva, I. V. & Geim, A. K. Unimpeded permeation of water through helium-leak–tight graphene-based membranes. *Science* **335**, 442-444 (2012).

3 Owens, D. K. & Wendt, R. C. Estimation of the surface free energy of polymers. *J. Appl. Polym. Sci.* **13**, 1741-1747 (1969).

4 Kaelble, D. H. Dispersion-polar surface tension properties of organic solids. *J. Adhesion* **2**, 66-81 (1970).

5 Rojewska, M., Skrzypiec, M. & Prochaska, K. The wetting properties of langmuir–blodgett and langmuir–schaefer films formed by DPPC and POSS compounds. *Chem. Phys. Lipids* **221**, 158-166 (2019).

6 Hu, M. & Mi, B. Enabling graphene oxide nanosheets as water separation membranes. *Environ. Sci. Technol.* **47**, 3715-3723 (2013).

7 Yang, Q. *et al.* Ultrathin graphene-based membrane with precise molecular sieving and ultrafast solvent permeation. *Nat. Mater.* **16**, 1198-1202 (2017).

8 Morelos-Gomez, A. *et al.* Effective NaCl and dye rejection of hybrid graphene oxide/graphene layered membranes. *Nat. Nanotech.* **12**, 1083-1088 (2017).

9 Zhao, G., Hu, R., Zhao, X., He, Y. & Zhu, H. High flux nanofiltration membranes prepared with a graphene oxide homo-structure. *J. Membr. Sci.* **585**, 29-37 (2019).

10 Li, W., Wu, W. & Li, Z. Controlling interlayer spacing of graphene oxide membranes by external pressure regulation. *ACS Nano* **12**, 9309-9317 (2018).

11 Kang, K. M. *et al.* Selective molecular separation on $Ti_3C_2T_x$–graphene oxide membranes during pressure-driven filtration: Comparison with graphene oxide and MXenes. *ACS Appl. Mater. Interfaces* **9**, 44687-44694 (2017).

12 Akbari, A. *et al.* Large-area graphene-based nanofiltration membranes by shear alignment of discotic nematic liquid crystals of graphene oxide. *Nat. Commun.* **7**, 10891 (2016).

13 Han, Y., Jiang, Y. & Gao, C. High-flux graphene oxide nanofiltration membrane intercalated by carbon nanotubes. *ACS Appl. Mater. Interfaces* **7**, 8147-8155 (2015).

14 Lim, M.-Y. *et al.* Cross-linked graphene oxide membrane having high ion selectivity and antibacterial activity prepared using tannic acid-functionalized graphene oxide and polyethyleneimine. *J. Membr. Sci.* **521**, 1-9 (2017).

15 Li, Y. *et al.* Thermally reduced nanoporous graphene oxide membrane for desalination. *Environ. Sci. Technol.* **53**, 8314-8323 (2019).

16 Chen, L. *et al.* Ion sieving in graphene oxide membranes via cationic control of interlayer spacing. *Nature* **550**, 380-383 (2017).

17 Nan, Q., Li, P. & Cao, B. Fabrication of positively charged nanofiltration membrane via the layer-by-layer assembly of graphene oxide and polyethylenimine for desalination. *Appl. Surf. Sci.* **387**, 521-528 (2016).

18 Xu, X.-L. *et al.* Graphene oxide nanofiltration membranes stabilized by cationic porphyrin for high salt rejection. *ACS Appl. Mater. Interfaces* **8**, 12588-12593 (2016).

19 Kim, D. W., Choi, J., Kim, D. & Jung, H.-T. Enhanced water permeation based on nanoporous

multilayer graphene membranes: the role of pore size and density. *J. Mater. Chem. A* **4**, 17773-17781 (2016).

20 Chen, X., Qiu, M., Ding, H., Fu, K. & Fan, Y. A reduced graphene oxide nanofiltration membrane intercalated by well-dispersed carbon nanotubes for drinking water purification. *Nanoscale* **8**, 5696-5705 (2016).

21 Yuan, S. *et al.* Minimizing non-selective nanowrinkles of reduced graphene oxide laminar membranes for enhanced NaCl rejection. *Environ. Sci. Technol. Lett.* **7**, 273-279 (2020).

22 Guan, K. *et al.* Nanochannel-confined charge repulsion of ions in a reduced graphene oxide membrane. *J. Mater. Chem. A* **8**, 25880-25889 (2020).

23 Lee, C. S., Kim, I., Jang, J. W., Yoon, D. s. & Lee, Y. J. Aquaporin-incorporated graphene-oxide membrane for pressurized desalination with superior integrity enabled by molecular recognition. *Adv. Sci.* **8**, 2101882 (2021).

24 Yuan, B. *et al.* Cross-linked graphene oxide framework membranes with robust nano-channels for enhanced sieving ability. *Environ. Sci. Technol.* **54**, 15442-15453 (2020).

25 Wang, Z. *et al.* Graphene oxide nanofiltration membranes for desalination under realistic conditions. *Nat. Sustain.* **4**, 402-408 (2021).

26 Xing, J. *et al.* Improving the performance of the lamellar reduced graphene oxide/molybdenum sulfide nanofiltration membrane through accelerated water-transport channels and capacitively enhanced charge density. *Environ. Sci. Technol.* **57**, 615-625 (2023).

27 Shen, J. *et al.* Fast water transport and molecular sieving through ultrathin ordered conjugated-polymer-framework membranes. *Nat. Mater.* **21**, 1183-1190 (2022).

28 Guan, K. *et al.* Deformation constraints of graphene oxide nanochannels under reverse osmosis. *Nat. Commun.* **14**, 1016 (2023).

29 Al-Gamal, A. Q., Saleh, T. A. & Alghunaimi, F. I. Nanofiltration membrane with high flux and oil rejection using graphene oxide/*β*-cyclodextrin for produced water reuse. *Mater. Today Commun.* **31**, 103438 (2022).

30 Han, Y., Xu, Z. & Gao, C. Ultrathin graphene nanofiltration membrane for water purification. *Adv. Funct. Mater.* **23**, 3693-3700 (2013).

31 Wei, Y. *et al.* Declining flux and narrowing nanochannels under wrinkles of compacted graphene oxide nanofiltration membranes. *Carbon* **108**, 568-575 (2016).

32 Hung, W.-S. *et al.* Graphene-induced tuning of the *d*-spacing of graphene oxide composite nanofiltration membranes for frictionless capillary action-induced enhancement of water permeability. *J. Mater. Chem. A* **6**, 19445-19454 (2018).

33 Zhang, Z. *et al.* Interfacial force-assisted in-situ fabrication of graphene oxide membrane for desalination. *ACS Appl. Mater. Interfaces* **10**, 27205-27214 (2018).

34 Rajesh, S. & Bose, A. B. Development of graphene oxide framework membranes via the “from” and “to” cross-linking approach for ion-selective separations. *ACS Appl. Mater. Interfaces* **11**, 27706-27716 (2019).

35 Chen, L. *et al.* A large-area free-standing graphene oxide multilayer membrane with high stability for nanofiltration applications. *Chem. Eng. J.* **345**, 536-544 (2018).

36 Zhou, X. *et al.* Intrapore energy barriers govern ion transport and selectivity of desalination membranes. *Sci. Adv.* **6**, eabd9045 (2020).

37 Zhai, X., Wang, Y.-L., Dai, R., Li, X. & Wang, Z. Roles of anion–cation coupling transport and dehydration-induced ion–membrane interaction in precise separation of ions by nanofiltration

membranes. *Environ. Sci. Technol.* **56**, 14069-14079 (2022).
38 Gao, J. *et al.* High-performance ionic diode membrane for salinity gradient power generation. *J. Am. Chem. Soc.* **136**, 12265-12272 (2014).
39 Zhang, H., Quan, X., Chen, S., Yu, H. & Niu, J. Electrokinetic enhancement of water flux and ion rejection through graphene oxide/carbon nanotube membrane. *Environ. Sci. Technol.* **54**, 15433-15441 (2020).
40 Chen, B., Jiang, H., Liu, X. & Hu, X. Observation and analysis of water transport through graphene oxide interlamination. *J. Phys. Chem. C* **121**, 1321-1328 (2017).
41 Dai, H., Xu, Z. & Yang, X. Water permeation and ion rejection in layer-by-layer stacked graphene oxide nanochannels: A molecular dynamics simulation. *J. Phys. Chem. C* **120**, 22585-22596 (2016).
42 Wei, N., Peng, X. & Xu, Z. Understanding water permeation in graphene oxide membranes. *ACS Appl. Mater. Interfaces* **6**, 5877-5883 (2014).
43 Wander, M. C. F. & Shuford, K. L. Electrolyte effects in a model system for mesoporous carbon electrodes. *J. Phys. Chem. C* **115**, 4904-4908 (2011).
44 Willcox, J. A. L. & Kim, H. J. Molecular dynamics study of water flow across multiple layers of pristine, oxidized, and mixed regions of graphene oxide. *ACS Nano* **11**, 2187-2193 (2017).
45 Wei, N., Peng, X. & Xu, Z. Breakdown of fast water transport in graphene oxides. *Phys. Rev. E* **89**, 012113 (2014).
46 Li, W. *et al.* Molecular dynamics simulations of CO_2/N_2 separation through two-dimensional graphene oxide membranes. *J. Phys. Chem. C* **120**, 26061-26066 (2016).
47 Vácha, R., Jungwirth, P., Chen, J. & Valsaraj, K. Adsorption of polycyclic aromatic hydrocarbons at the air–water interface: Molecular dynamics simulations and experimental atmospheric observations. *Phys. Chem. Chem. Phys.* **8**, 4461-4467 (2006).
48 Fu, Z. *et al.* Adsorption of nitrobenzene on the surface of ice: A grand canonical monte carlo simulation study. *J. Phys. Chem. C* **121**, 15746-15755 (2017).
49 Yang, E. *et al.* Enhanced desalination performance of forward osmosis membranes based on reduced graphene oxide laminates coated with hydrophilic polydopamine. *Carbon* **117**, 293-300 (2017).
50 Liu, H., Wang, H. & Zhang, X. Facile fabrication of freestanding ultrathin reduced graphene oxide membranes for water purification. *Adv. Mater.* **27**, 249-254 (2015).
51 Fan, X., Liu, Y. & Quan, X. A novel reduced graphene oxide/carbon nanotube hollow fiber membrane with high forward osmosis performance. *Desalination* **451**, 117-124 (2019).
52 Kang, H. *et al.* Interlamination restrictive effect of carbon nanotubes for graphene oxide forward osmosis membrane via layer by layer assembly. *Appl. Surf. Sci.* **465**, 1103-1106 (2019).
53 Zhao, W. *et al.* Thin-film nanocomposite forward-osmosis membranes on hydrophilic microfiltration support with an intermediate layer of graphene oxide and multiwall carbon nanotube. *ACS Appl. Mater. Interfaces* **10**, 34464-34474 (2018).
54 Shen, L., Xiong, S. & Wang, Y. Graphene oxide incorporated thin-film composite membranes for forward osmosis applications. *Chem. Eng. Sci.* **143**, 194-205 (2016).
55 Rastgar, M., Shakeri, A., Bozorg, A., Salehi, H. & Saadattalab, V. Highly-efficient forward osmosis membrane tailored by magnetically responsive graphene oxide/Fe_3O_4 nanohybrid. *Appl. Surf. Sci.* **441**, 923-935 (2018).
56 Wu, X., Field, R. W., Wu, J. J. & Zhang, K. Polyvinylpyrrolidone modified graphene oxide as a modifier for thin film composite forward osmosis membranes. *J. Membr. Sci.* **540**, 251-260 (2017).
57 Zhang, M. *et al.* Engineering a nanocomposite interlayer for a novel ceramic-based forward osmosis

membrane with enhanced performance. *Environ. Sci. Technol.* **54**, 7715-7724 (2020).

58 Jain, H. *et al.* Fabrication and characterization of high-performance forward-osmosis membrane by introducing manganese oxide incited graphene quantum dots. *J. Environ. Manage.* **305**, 114335 (2022).

59 Li, P. *et al.* Synergistic effect of polyvinyl alcohol sub-layer and graphene oxide condiment from active layer on desalination behavior of forward osmosis membrane. *J. Taiwan Inst. Chem. E.* **112**, 366-376 (2020).

60 Shokrgozar Eslah, S., Shokrollahzadeh, S., Moini Jazani, O. & Samimi, A. Forward osmosis water desalination: Fabrication of graphene oxide-polyamide/polysulfone thin-film nanocomposite membrane with high water flux and low reverse salt diffusion. *Sep. Sci. Technol.* **53**, 573-583 (2018).

61 Akther, N. *et al.* Influence of graphene oxide lateral size on the properties and performances of forward osmosis membrane. *Desalination* **484**, 114421 (2020).

62 Choi, H.-g., Shah, A. A., Nam, S.-E., Park, Y.-I. & Park, H. Thin-film composite membranes comprising ultrathin hydrophilic polydopamine interlayer with graphene oxide for forward osmosis. *Desalination* **449**, 41-49 (2019).

63 Wu, X. *et al.* Scalable $Ti_3C_2T_x$ MXene interlayered forward osmosis membranes for enhanced water purification and organic solvent recovery. *ACS Nano* **14**, 9125-9135 (2020).

64 Hadadpour, S. *et al.* Synthesis and characterization of novel thin film composite forward osmosis membrane using charcoal-based carbon nanomaterials for desalination application. *J. Environ. Chem. Eng.* **9**, 104880 (2021).

65 Rastgar, M., Bozorg, A. & Shakeri, A. Novel dimensionally controlled nanopore forming template in forward osmosis membranes. *Environ. Sci. Technol.* **52**, 2704-2716 (2018).

66 Shamaei, L., Karami, P., Khorshidi, B., Farnood, R. & Sadrzadeh, M. Novel lignin-modified forward osmosis membranes: Waste materials for wastewater treatment. *ACS Sustain. Chem. Eng.* **9**, 15768-15779 (2021).

67 Sun, P.-F. *et al.* Interlayered forward osmosis membranes with $Ti_3C_2T_x$ MXene and carbon nanotubes for enhanced municipal wastewater concentration. *Environ. Sci. Technol.* **55**, 13219-13230 (2021).

68 Shabani, Z., Mohammadi, T., Kasiri, N. & Sahebi, S. Thin-film nanocomposite forward osmosis membranes prepared on PVC substrates with polydopamine functionalized Zr-based metal organic frameworks. *Ind. Eng. Chem. Res.* **61**, 7067-7079 (2022).

69 Bagherzadeh, M., Bayrami, A. & Amini, M. Thin-film nanocomposite forward osmosis membranes modified with Zr-based metal–organic framework to improve desalination performance. *Appl. Organomet. Chem.* **34**, e5339 (2020).

70 Ma, D., Peh, S. B., Han, G. & Chen, S. B. Thin-film nanocomposite (TFN) membranes incorporated with super-hydrophilic metal–organic framework (MOF) UiO-66: Toward enhancement of water flux and salt rejection. *ACS Appl. Mater. Interfaces* **9**, 7523-7534 (2017).

71 Hakimi, G., Shakeri, A., Yassari, M. & Salehi, H. Enhancing the permeability and fouling resistance of thin-film composite forward osmosis membranes via poly(ethylene glycol) diamine as a comonomer. *ACS Appl. Polym. Mater.* **5**, 567-575 (2023).

72 Soyekwo, F., Wen, H., Dan, L. & Liu, C. Crumpled globule-heterotextured polyamide membrane interlayered with protein–polyphenol nanoaggregates for enhanced forward osmosis performance. *ACS Appl. Mater. Interfaces* **14**, 24806-24819 (2022).

73 Zhao, W. *et al.* Polyamide membrane tailored by codeposition of a UiO-66 metal–organic framework nanostructure-based interlayer for forward osmosis. *ACS Appl. Nano Mater.* **6**, 6901-6910 (2023).

74 Liu, P., Zhang, S., Han, R., Bu, X. & Jian, X. Effect of additives on the performance of PPBES composite forward osmosis hollow fiber membranes. *ACS Omega* **5**, 23148-23156 (2020).

75 Wang, Y., Lian, T., Tarakina, N. V., Yuan, J. & Antonietti, M. Lamellar carbon nitride membrane for enhanced ion sieving and water desalination. *Nat. Commun.* **13**, 7339 (2022).

76 Wang, W. *et al.* High-surface-area functionalized nanolaminated membranes for energy-efficient nanofiltration and desalination in forward osmosis. *Nat. Water* **1**, 187-197 (2023).

77 Dahanayaka, M. *et al.* Graphene membranes with nanoslits for seawater desalination via forward osmosis. *Phys. Chem. Chem. Phys.* **19**, 30551-30561 (2017).

78 Liu, B., Law, A. W.-K. & Zhou, K. Strained single-layer C_2N membrane for efficient seawater desalination via forward osmosis: A molecular dynamics study. *J. Membr. Sci.* **550**, 554-562 (2018).

79 Gogoi, A., Anki Reddy, K., Senthilmurugan, S. & Kumar Mondal, P. Dehydration of acetic acid using layered graphene oxide (GO) membrane through forward osmosis (FO) process: a molecular dynamics study. *Mol. Simulat.* **46**, 1500-1508 (2020).

80 Delley, B. From molecules to solids with the $DMol^3$ approach. *J. Chem. Phys.* **113**, 7756-7764 (2000).

81 Meng, R. *et al.* First principles investigation of small molecules adsorption on antimonene. *IEEE Electr. Device L.* **38**, 134-137 (2017).
