# Supplementary material for: Electrostatic-induced ion-confined partitioning in graphene nanolaminate membrane for breaking anion–cation co-transport to enhance desalination
Source: Nat Commun. 2024 May 21;15:4324. doi: 10.1038/s41467-024-48681-8 (PMC11109394; doi:10.1038/s41467-024-48681-8)
Supplement: Supplementary file 2 — Peer Review File [file 41467_2024_48681_MOESM2_ESM.pdf]

nature portfolio

Peer Review File

Electrostatic-Induced Ion Confined Partitioning in Graphene Nanolaminate Membrane for Breaking Anion-Cation Co-Transport to Enhance Desalination

Open Access This file is licensed under a Creative Commons Attribution 4.0 International License, which permits use, sharing, adaptation, distribution and reproduction in any medium or format, as long as you give appropriate credit to the original author(s) and the source, provide a link to the Creative Commons license, and indicate if changes were made. In the cases where the authors are anonymous, such as is the case for the reports of anonymous peer reviewers, author attribution should be to 'Anonymous Referee' followed by a clear attribution to the source work. The images or other third party material in this file are included in the article's Creative Commons license, unless indicated otherwise in a credit line to the material. If material is not included in the article's Creative Commons license and your intended use is not permitted by statutory regulation or exceeds the permitted use, you will need to obtain permission directly from the copyright holder. To view a copy of this license, visit http://creativecommons.org/licenses/by/4.0/.

Editorial Note: This manuscript has been previously reviewed at another journal that is not operating a transparent peer review scheme. This document only contains reviewer comments and rebuttal letters for versions considered at *Nature Communications*.
